# Supplementary material for: Cases and distribution of visceral leishmaniasis in western São Paulo: A neglected disease in this region of Brazil
Source: PLoS Negl Trop Dis. 2021 Jun 15;15(6):e0009411. doi: 10.1371/journal.pntd.0009411 (PMC8232419; doi:10.1371/journal.pntd.0009411)

PLOS Neglected Tropical Diseases
The burden of visceral leishmaniasis in western São Paulo: a neglected disease in this region of Brazil
--Manuscript Draft--

**Manuscript Number:**	PNTD-D-20-01367
**Full Title:**	The burden of visceral leishmaniasis in western São Paulo: a neglected disease in this region of Brazil
**Short Title:**	Visceral leishmaniais in western São Paulo
**Article Type:**	Research Article
**Keywords:**	Entomological survey; Entomologial survey; Canine visceral leishmaniasis; Environmental risk factors; socioeconomic risk factors, Spatiotemporal epidemiology
**Abstract:**	Visceral leishmaniasis (VL) is one of the most prevalent parasitic diseases worldwide. In 2018, 97% of the total number of cases in Latin America were reported in Brazil. In São Paulo, the most populous state, currently 16.9% of the municipalities have recorded human visceral leishmaniasis (HVL), with 17.6% of infected individuals from the western region. Worldwide, VL is linked to environmental and socioeconomic conditions that increase vulnerability to the disease. We determined the burden of VL in municipalities of the Regional Network for Health Assistance (RNHA11), western São Paulo, in a multiscale study that focuses on the city of Teodoro Sampaio, part of the Pontal of Paranapanema, to study this neglected disease in a neglected region of São Paulo, Brazil. Environmental databases obtained from public records (archival databases) were used for this regional study of HVL and the Human Development Index. For the local study in Teodoro Sampaio, an entomological survey from 2009 to 2015, a canine visceral leishmaniasis (CVL) survey from 2010 to 2018, and a human serum survey in 2018 were carried out. Maps showing the spatiotemporal epidemiology of CVL were constructed. From 2005 to 2018, Lutzomyia longipalpis was found in 77.8%, CVL in 68%, and HVL in 42.2% of the municipalities, and 537 individuals were infected with HVL. Dispersion occurred from the epicenter in the north and followed the central and radial highways reaching Teodoro Sampaio, in the south where Lu. longipalpis and CVL were found in 2010 and HVL in 2018 with an interval lag of 8 years. Critical hotspots of CVL were found in the periphery. In RNHA11, a complex array of socioeconomic and environmental factors may be fueling the epidemic and sustaining endemic transmission of VL, adding to the study of a neglected disease in a neglected region of São Paulo, Brazil.
**Additional Information:**	
**Question**	**Response**
**Financial Disclosure**<br><br>Enter a financial disclosure statement that describes the sources of funding for the work included in this submission. Review the submission guidelines for detailed requirements. View published research articles from *PLOS NTDs* for specific examples.<br><br>This statement is required for submission and **will appear in the published article** if the submission is accepted. Please make sure it is accurate.	The author(s) received no specific funding for this work.

Powered by Editorial Manager® and ProduXion Manager® from Aries Systems Corporation

**Unfunded studies**<br><br>Enter: *The author(s) received no specific funding for this work.*<br><br>**Funded studies**<br><br>Enter a statement with the following details:<br>• Initials of the authors who received each award<br>• Grant numbers awarded to each author<br>• The full name of each funder<br>• URL of each funder website<br>• Did the sponsors or funders play any role in the study design, data collection and analysis, decision to publish, or preparation of the manuscript?<br>• **NO** - Include this sentence at the end of your statement: *The funders had no role in study design, data collection and analysis, decision to publish, or preparation of the manuscript.*<br>• **YES** - Specify the role(s) played.<br><br>* typeset	
**Competing Interests**<br><br>Use the instructions below to enter a competing interest statement for this submission. On behalf of all authors, disclose any competing interests that could be perceived to bias this work—acknowledging all financial support and any other relevant financial or non-financial competing interests.<br><br>This statement **will appear in the published article** if the submission is accepted. Please make sure it is accurate. View published research articles from *PLOS NTDs* for specific examples.	The authors have declared that no competing interests exist.

Powered by Editorial Manager® and ProduXion Manager® from Aries Systems Corporation

**NO authors have competing interests**<br><br>Enter: *The authors have declared that no competing interests exist.*<br><br>**Authors with competing interests**<br><br>Enter competing interest details beginning with this statement:<br><br>*I have read the journal's policy and the authors of this manuscript have the following competing interests: [insert competing interests here]*<br><br>* typeset	
**Data Availability**<br><br>Authors are required to make all data underlying the findings described fully available, without restriction, and from the time of publication. PLOS allows rare exceptions to address legal and ethical concerns. See the PLOS Data Policy and FAQ for detailed information.<br><br>A Data Availability Statement describing where the data can be found is required at submission. Your answers to this question constitute the Data Availability Statement and **will be published in the article**, if accepted.	No - some restrictions will apply

Powered by Editorial Manager® and ProduXion Manager® from Aries Systems Corporation

**Important:** Stating 'data available on request from the author' is not sufficient. If your data are only available upon request, select 'No' for the first question and explain your exceptional situation in the text box.<br><br>Do the authors confirm that all data underlying the findings described in their manuscript are fully available without restriction?	
**Describe where the data may be found in full sentences. If you are copying our sample text, replace any instances of XXX with the appropriate details.**<br><br>• If the data are **held or will be held in a public repository**, include URLs, accession numbers or DOIs. If this information will only be available after acceptance, indicate this by ticking the box below. For example: *All XXX files are available from the XXX database (accession number(s) XXX, XXX.*).<br>• If the data are all contained **within the manuscript and/or Supporting Information files**, enter the following: *All relevant data are within the manuscript and its Supporting Information files.*<br>• If neither of these applies but you are able to provide **details of access elsewhere**, with or without limitations, please do so. For example:<br><br>*Data cannot be shared publicly because of [XXX]. Data are available from the XXX Institutional Data Access / Ethics Committee (contact via XXX) for researchers who meet the criteria for access to confidential data.*<br><br>*The data underlying the results presented in the study are available*	Some data cannot be shared publicly because they are not available on public sites but they can be obtained from the corresponding author under request.

from (include the name of the third party and contact information or URL).<br>• This text is appropriate if the data are owned by a third party and authors do not have permission to share the data.<br><br>* typeset	
Additional data availability information:	Tick here if the URLs/accession numbers/DOIs will be available only after acceptance of the manuscript for publication so that we can ensure their inclusion before publication.

Manuscript

Click here to access/download;Manuscript;MANUSCRIPT.docx

The burden of visceral leishmaniasis in western São Paulo: a neglected disease in this region of Brazil

Regiane Soares Santana[1], Karina Briguenti Souza[1], Fernanda Lussari[1], Elivelton Silva Fonseca[1], Cristiane Oliveira Andrade[2], Marcia Kaihara Meidas[3], Lourdes Aparecida Zampieri D'Andrea[4], Francisco Assis Silva[1], Edilson Ferreira Flores[5], Ivete Rocha Anjolete[6], Luiz Euribel Prestes-Carneiro[1]*

[1]Department of Pós-Graduation, Environment and Regional Development Program, Oeste Paulista University, Presidente Prudente, São Paulo, Brazil

[2]Department of Control of Vectors, Teodoro Sampaio Municipality, Teodoro Sampaio, São Paulo, Brazil

[3]Bioclinic Clinical Laboratory, Teodoro Sampaio, São Paulo, Brazil

[4]Center for Biomedical Sciences and Regional Laboratory, Adolfo Lutz Institute, Presidente Prudente, São Paulo, Brazil

[5]Statistics Department, School of Sciences and Technology, São Paulo State University, Presidente Prudente Campus, Presidente Prudente, São Paulo, Brazil

[6]Supervision in Control of Endemics, Presidente Prudente, São Paulo, Brazil

*Corresponding author

luiz@unoeste.br (LEP-C)

The manuscript was edited by a native speaker: authorserv@gmail.com; Invoice no: 200103

Abstract

Visceral leishmaniasis (VL) is one of the most prevalent parasitic diseases worldwide. In 2018, 97% of the total numbers of cases in Latin America were reported in Brazil. In São Paulo, the most populous state, currently 16.9% of the municipalities have recorded human visceral leishmaniasis (HVL), with 17.6% of infected individuals from the western region. Worldwide, VL is linked to environmental and socioeconomic conditions that increase vulnerability to the disease. We determined ~~the burden~~ of VL in municipalities of the Regional Network for Health Assistance (RNHA11), western São Paulo, in a multiscale study that focuses on the city of Teodoro Sampaio, part of the Pontal of Paranapanema, to study this neglected disease ~~in a neglected~~ region of São Paulo, Brazil. Environmental databases obtained from public records ~~(archival databases)~~ were used for this regional study of HVL and the Human Development Index. For the local study in Teodoro Sampaio, an entomological survey from 2009 to 2015, a canine visceral leishmaniasis (CVL) survey from 2010 to 2018, and a human serum survey in 2018 were carried out. Maps showing the spatiotemporal epidemiology of CVL were constructed. From 2005 to 2018, *Lutzomyia longipalpis* was found in 77.8%, CVL in 68%, and HVL in 42.2% of the municipalities, and 537 individuals were infected with HVL. Dispersion occurred from the epicenter in the north ~~and followed the central and radial highways reaching~~ Teodoro Sampaio, in the south where *Lu. longipalpis* and CVL were found in 2010 and HVL in 2018 ~~with an interval lag of 8 years~~. Critical hotspots of CVL were found in the periphery. In RNHA11, a complex array of socioeconomic and environmental factors may be fueling the epidemic and sustaining endemic transmission of VL, adding to the study of a neglected disease in a ~~neglected~~ region of São Paulo, Brazil.

Author summary

Visceral leishmaniasis (VL) is considered the second most important disease caused by a protozoan worldwide. Currently, 97% of the cases in Latin America are in Brazil. In São Paulo state, since 1997, the disease has been found in an increasing number of municipalities, mainly in the western region. The reasons why VL is spreading in this region at alarming rates is not well understood, however, environmental and socioeconomic structures that increase vulnerability to the disease may be involved. From 2005 to 2018, vectors of *Lutzomyia longipalpis* were found in 77.8%, canine VL in 68%, and human VL in 44.4% of the 45 municipalities of the western region. Dispersion occurred from the epicenter in the north and followed central and radial highways, reaching Teodoro Sampaio in the south. Environment factors (artificial lakes and deforestation) and social factors (low Human Development Index and lack of a Center for Zoonosis Control for canine VL) are among the risk factors favoring the spread of the disease. Considered the last frontier and one of the poorest regions of São Paulo, our findings are relevant for planning interventions aimed at reducing the ~~burden~~ of VL in the region.

Introduction

Visceral leishmaniasis (VL) is one of the most prevalent parasitic diseases worldwide. In 2017, 94% of new cases occurred in seven countries: Brazil, Ethiopia, India, Kenya, Somalia, South Sudan, and Sudan [1]. In 2018, 97% of the total numbers of cases in Latin America were reported by Brazil [2]. Concentrated in rural areas of the northeast, the disease reached the periphery of some capital cities and in recent decades has spread to other regions. Currently, 25 of 27 Brazilian states notified autochthonous

cases of canine visceral leishmaniasis (CVL) and 23 notified cases of human visceral leishmaniasis (HVL) [3].

In São Paulo, the richest and most populous state, *Lutzomyia longipalpis* sandflies were found in 1997, infected dogs in 1998, and infected humans in 1999. In 2019, 106 of 645 (16.4%) municipalities registered cases of HVL [4]. Geographically, the state is divided into 15 mesoregions with 18 Regional Network for Health Assistance (RNHA; Rede Regional de Assistência a Saúde) for administrative health care. The western region, with 45 municipalities and an estimated population of 753,344 in 2018, according to the Brazilian Institute of Geography and Statistics (IBGE), is covered by RNHA11, mesoregion 8. VL reached São Paulo state through the western region coming from the bordering state of Mato Grosso do Sul (MS) [5]. In 2019, 3042 cases were notified and 537 (17.6%) were from the western region. Among the 45 municipalities of the western region, 32 (often called the Pontal of Paranapema, Paranapanema River Basin) are considered to be the poorest and asymmetric regions of São Paulo state [4].

Similar to other cities in this regional division, Teodoro Sampaio, considered the capital of Pontal of Paranapanema, has environmental and socioeconomic characteristics that may contribute to the dispersion of VL. Leishmaniasis is a poverty-related parasitic disease linked to socioeconomic disparities leading to malnutrition, illiteracy, poor housing, and lack of sanitation. Leishmaniasis is also linked to the impact of humans on the environment such as deforestation, enlarged artificial lakes, and construction of dams, and migration of people infected with VL from endemic to non-endemic areas [6].

In epidemiologic studies, geospatial analysis has been used successfully for vector-borne diseases as a tool for spatiotemporal analysis and to predict the influence of

endemic areas in the dispersion of the disease to other areas [7]. We determined the burden of VL in municipalities in RNHA11, western São Paulo, in a multiscale study that focuses on the city of Teodoro Sampaio, as part of the Pontal of Paranapanema, combining the study of a neglected disease in a ~~neglected~~ region of São Paulo, Brazil.

Methods

Study design and setting

In 2019, São Paulo, the richest and most populous state of Brazil, with an estimated population of 45,919,049, accounts for 21.8% of the entire population of Brazil, estimated to be 210,147,125 inhabitants according to IBGE (Fig 1). The state comprises 645 municipalities, bordering Minas Gerais (north and northeast), Rio de Janeiro (northeast), Paraná (south), MS (west), and the Atlantic Ocean (east) (Fig 1A). It is divided geographically into 15 mesoregions and 18 RNHAs for administrative health care. The western region, with 45 municipalities and an estimated population of 753,344 inhabitants (2018) is administered by RNHA11, located in Presidente Prudente mesoregion 8. The region is characterized by municipalities with a population <20,000 [8]. Among the 45 municipalities that are part of RHNA11 are the 23 municipalities, often called Pontal of Paranapema region, that covers an area of 18,392.16 km^2 and has an estimated population of 620,953 (IBGE 2018) (Fig 1A). Teodoro Sampaio (~~coordinates:~~ latitude 22º31′57″S and longitude 52º10′03″W) at an altitude of 321 m above sea level has an estimated population of 23,019 (IBGE 2018). It is a small-sized urban center located 651 km from the state capital~~, São Paulo~~. The municipality covers an area of 1556 km^2, currently the 8th largest municipality in São Paulo (IBGE) (Fig 1B and Ca). The muncipality has sandy soil and a typical tropical climate with dry winters and wet summers, with an average annual temperature of 29°C. The canine population

was estimated to be 5754 animals, with a ratio of 1 dog per 4 inhabitants as previously determined for the region [9,10]. Devil's Hill State Park (Parque Estadual Morro do Diabo) is 6.3 km from the urban area of Teodoro Sampaio. Covering an area of 338.5 km^2, it is one of the largest stretches of Tropical Forest in São Paulo state (Fig 1 and Cb). In the 1950s, the whole region of RNHA11 was covered by a Tropical Forest that was almost completely destroyed. The Tropical Forest in Pontal of Paranapanema was extensively destroyed, especially in the middle of the 20th century. More than 3000 km^2 were reduced to 370 km^2 in less than 20 years (12.3%), including the Devil's Hill State Park and other small fragments. Flowing along the border of the city, the Paranapanema River is one of the biggest and most important rivers in the southeastern region of Brazil, and is a natural border between São Paulo and Paraná states (Fig 1Cc). It is 930 km long and since the 1980s, it includes 11 large artificial lakes and an extensive flooded area, supporting hydroelectric plants and bridges. These bridges link dozens of small towns and villages connected by a large number of highways (Fig 1A and B).

Fig 1. Road network, rivers and artificial lakes, and location of Teodoro Sampaio in RNHA11 of São Paulo state. (A) The highway network between the municipalities of RNHA11 and Teodoro Sampaio. Artificial lakes and bridges on the Paraná River linking RNHA11 municipalities to well-known endemic regions in Mato Grosso do Sul state, and bridges over artificial lakes in the Paranapanema river linking regions with VL to VL-free areas in Paraná state. (B) The municipality of Teodoro Sampaio is bordered by the artificial lake of Primavera Dam in the Paraná River and by Rosana Dam in the Paranapanema River. (C) A Google Earth image of the municipality of Teodoro Sampaio: (a) the urban area of Teodoro Sampaio, (b) the Devil's Hill State Park, 6.3 km from the urban area, (c) the artificial lake and islands of the Paranapanema River, 0.9 km from the urban area,

Archival databases were obtained from Brazilian public health agencies and some are available to download upon formal request: Supervision in Control of Epidemic (SUCEN), http://www.saude.sp.gov.br/sucen-superintendencia-de-controle-de-

endemias/homepage/downloads/arquivos-leishmaniose-visceral; Center of Regional Laboratory of the Adolfo Lutz Institute of Presidente Prudente V (CRL-CBSALI-PPV), http://www.ial.sp.gov.br/ial/o-ial/quem-e-quem/centro-de-laboratorio-regionais; National System of Diseases Notification (SINAN), http://portalsinan.saude.gov.br/; Epidemiological Surveillance Center (CVE-SP), http://www.saude.sp.gov.br/cve-centro-de-vigilancia-epidemiologica-prof.-alexandre-vranjac/areas-de-vigilancia/doencas-de-transmissao-por-vetores-e-zoonoses/.

Human visceral leishmaniasis in municipalities in RNHA11 mesoregion and in the city of Teodoro Sampaio

Data on human leishmaniasis were obtained from public agencies, including the Epidemiological Surveillance Center of São Paulo, which organizes and publishes data on recorded infectious diseases in São Paulo state annually. The sole case of HVL in Teodoro Sampaio was referred to the Regional Hospital of Presidente Prudente, a tertiary, university, public, and reference center for treatment of HVL in RNHA11 mesoregion. Confidentiality was assured. The case was confirmed in 2018 by clinical/epidemiologic criteria and laboratory diagnostics according to the Manual of Surveillance and Control of Visceral Leishmaniasis of São Paulo state (VLCPSP) [11].

The entomologic survey in Teodoro Sampaio

Teodoro Sampaio covers two areas with regard to operational disease control programs (e.g. dengue fever surveillance and control, CVL and HVL surveillance and control). The two areas are divided into four sectors and the sectors are divided into blocks (Fig 2A and B). Entomologic surveys are overseen by the VLCPSP, aiming to monitor the distribution of *Lu. longipalpis* in non-vulnerable, non-receptive silent municipalities, as defined by the absence of confirmed autochthonous cases of HVL and

CVL, without the presence of the vector. In these municipalities, an entomologic survey is recommended for the detection of the presence of *Lu. longipalpis* and to provide information on its distribution, allowing identification of the risk areas where control measures should be intensified [3,7]. In the urban area of Teodoro Sampaio, the entomologic survey began in 2009 during the most favorable periods for detecting the presence of the vector, from October to January and from February to April, and extended through May as needed. To standardize collections, the number (ranging from 1 to 40) and frequency (ranging from 1 to 39) of CDC light traps (Horst Ltd, São Paulo, Brazil) installed varied according to the density and infestation of sand flies. The traps were placed approximately 1 m from the ground in places susceptible to the presence of phlebotomies and/or in the shelters of domestic animals, with an average temperature above 20°C and relative humidity >70%. In each sector of the different areas, four areas with risk factors for the presence of phlebotomies were selected, including a large peri-domicile, and areas with abundant vegetation, accumulation of organic matter in the soil, and the presence of domestic animals from which the female sand fly can potentially obtain an infected blood meal. The traps were operated between 17:00 and 07:00 h. Four traps were installed for three nights in each sector. After capture, nylon-mesh cages were placed in plastic bags, tagged, and refrigerated (1°C–7°C) until identification was performed by the Regional Entomology Laboratories of SUCEN, Coordination of Disease Control, Secretary of State of Health, located in Presidente Prudente, São Paulo state. The sand flies were processed and identified using the taxonomic key of Galati [12]. In 2009 when the survey started in the urban area of Teodoro Sampaio, the traps were installed in four areas concomitantly. In 2010, the entomology survey ended after the vector was found in all areas. Data on the presence of *Lu. longipalpis* in the municipalities of RNHA11 were obtained from SUCEN.

Fig 2. Kernel of spatiotemporal distribution and density estimation of CVL seropositive euthanized dogs in Teodoro Sampaio, 2010–2018. Kernel of the spatiotemporal distribution of CVL diagnosed and euthanized dogs (A). The hotspots represent areas where a higher density of seropositive dogs was observed, from 2010 to 2018 (B). In the map legend, the lighter colors represent the low density of CVL cases and the darker colors represent the presence of hotspots.

Canine visceral leishmaniasis survey in Teodoro Sampaio and the presence of the Center of Zoonosis Control in RNHA11 mesoregion

The canine survey took place in four sectors of the urban area of Teodoro Sampaio (Fig 2A and B), from January 2010 to December 2018, by a passive or house-to-house survey conducted by the Center of Zoonosis Control (CZC). Briefly, an active house-to-house search was conducted when *Lu. longipalpis* was found in a particular area of the city or every time a dog was diagnosed with CVL; immediately, all households within a radius of 200 m were visited and the dogs were tested for CVL. A passive survey was conducted when a dog's owner found the symptoms described by the CZC as suspicious for CVL as previously published. We used recorded data of 3749 domiciled dogs of different ages, submitted to serum survey, using antibody tests to detect *Leishmania*, according to the Brazilian Ministry of Health and VLCPSP guidelines. In Teodoro Sampaio, the Center of Regional Laboratory of the Adolfo Lutz Institute of Presidente Prudente (CRL-CBSALI-PPV) is responsible for the diagnostic tests for CVL. The VLCSP supervises the main actions to reduce morbidity and mortality, aimed at early diagnosis and treatment of human cases, vector control, identification, and euthanasia of seropositive domestic dogs. In 2009, the Brazilian Ministry of Health began using enzyme-linked immunosorbent assays (ELISAs), produced by Bio-Manguinhos/Fiocruz, Brazilian Ministry of Health, Rio de Janeiro, Brazil, for screening and confirmatory tests in CVL serological surveys. For ELISA-positive samples, a

confirmatory test using IFAT, produced by Bio-Manguinhos/Fiocruz, was performed. From December 2011 to the present, the Brazilian Ministry of Health replaced the ELISA/IFAT tests with a new protocol using a dual-path platform CVL rapid test, produced by Bio-Manguinhos/Fiocruz, with ELISA-CVL as a confirmatory test. Animals with positive samples in the screening test (ELISA from January 2009 to November 2011 and the dual-path platform CVL rapid test from December 2011 until the present and a negative result in the confirmatory test (IFAT and ELISA, respectively) were re-sampled. Only concordant samples that were positive in the screening test and positive in the confirmatory test were considered seropositive [10]. Data on the presence and characteristics in each municipality under the CZC in the RNHA11 mesoregion were obtained from Adolfo Lutz Institute of Presidente Prudente [13].

Human serological survey and laboratory diagnosis

In July 2018, a serological survey was conducted on 159 individuals aged from 18 to 65 years from four different locations: a health care center, a Family Health Strategy Unit; the Social Assistance Reference Center; a State Technical School; and in individuals attending a private clinical laboratory. Blood was collected blood collections tubes under vacuum with and without EDTA as an anticoagulant. Serum was separated by centrifugation and stored at −200°C until use. The analysis was performed at the CRL-CBSALI-PPV using a qualitative test for the detection of antibodies against HVL by ELISA, according to the manufacturer’s instructions (LEISHMANIA ELISA IgG+IgM; VIRCELL, Santa Fe, Granada, Spain). The test uses recombinant antigens capable of fixing specific antibodies to *Leishmania chagasi* with a sensitivity of 97% and specificity of 99%. Positive tests were confirmed by indirect immunofluorescence (Bio-Manguinhos/Fiocruz, Ministry of Health, Rio de Janeiro, Brazil). Hemograms and

hepatic enzymes were determined because hepatic function may be affected and pancytopenia may be present in patients infected with VL. Hemograms were performed using a flow cytometer flux counter (Pentra 80, HoribaDiagnostics, Montpellier, France) and the differential leukocyte count was compared with direct microscopic observation of blood smears. Alanine aminotransferase (ALT), aspartate aminotransferase (AST), and albumin were assessed using automated systems according to the manufacturer’s instructions; the normal ranges were 7–55 U/L, 8–43 U/L, and 3.5–5 g/dL, respectively. A total of 16,636 individuals were considered to determine the sample size. These samples were processed by Laboratório Bioclínico, Teodoro Sampaio, SP, Brazil.

Kernel maps construction

Dogs that underwent the serological survey and those with positive serology were registered according to the location used by the municipal health surveillance service. The city was divided into four sectors, starting from the northeast of the urban area in a clockwise direction. These sectors, in turn, were divided into 432 blocks, arranged in a polygon shape, used to generate the Geographic Information System and served as a basis for indexing the attributes of the surveyed blocks. With the geometric points generated per block, it was possible to visualize the number of dogs with CVL in each block, the number of dogs in the census for each block, and the number of euthanized dogs per block. The number of positive dogs per block is not consistent with the number of euthanized dogs, because there were some cases in which euthanasia of dogs that tested positive was not performed. For the procedure that identified hot areas of CVL infection, we used the Kernel density maps, using the euthanized dogs as a population field to generate a continuous surface to the urban area of the Teodoro Sampaio. A raster surface was generated using the Gaussian equation, with a search ratio equivalent

to a block (100 m) and a pixel size equivalent to a household (10 m). The coordinate system used as reference was WGS1984. Regarding the parameters for reading the data for analysis, the urban area of Teodoro Sampaio measures 4 × 3 km^2. ArcGIS 10.7 and ArcGis Pro were used for data analysis and layout design.

Mapping the Human Development Index in the RNHA11 mesoregion

The Human Development Index (HDI) was used as an indicator for understanding the socioeconomic profile of the municipalities of São Paulo state. The HDI is a well-known and comparable indicator worldwide, reproducing studies in other states and regions countrywide. The HDI value of the 645 municipalities in the state of São Paulo was obtained in SEADE, from 1999 to 2016, coded and classified by the denominated quintile as a reference parameter. Thematic maps were created for the HDI using ArcGis software. The HDI was analyzed with support in a predictive method of interpolation surface generation: local polynomial interpolation (LPI). The Gaussian kernel method was used to generate the surface, classified according to the quintile. The gain in the LPI analysis in relation to the global polynomial interpolation presupposes it overlaps in several concentrations that a spatial representation may have, in our study, through a point in the centroid city of São Paulo state.

Ethical statement

The project was approved by the ethics committee of Unoeste (number 4030). Canine samples were collected with the written informed consent of owners. Ethical approval was obtained from the Adolfo Lutz/Pasteur Institute Ethics Committee on the Use of Animals (number 02/2016). The study is for scientific research purposes and is in compliance with Federal Law no. 11794 of October 8, 2008, Decree no. 6899 of July 15, 2009, and in accordance with regulations outlined by The National Council for

Animal Experimentation Control (Conselho Nacional para Controle da Experimentação Animal, CONCEA).

Statistical analysis

The results are shown as means standard error of the mean (SEM) (for normally distributed variables). Dichotomous and nominal variables are expressed as frequencies and percentages. Pearson's correlation coefficient was used to describe the functional relationship between the number of cases of HVL per 10,000 inhabitants and the HDI of each municipality. Statistical analysis was performed using GraphPad Software (San Diego, CA, USA) and the Sigma-Stat program (Systat Software, Richmond, CA, USA). Maps were constructed using a geographic information system.

Results

Potential dispersion routes of infection: RNHA11 mesoregion and Pontal of Paranapanema hub

RNHA11 is connected by two important radial highways: (1) the Marechal Candido Rondon (SP 300) linking two VL endemic regions: Três Lagoas, on the border of MS and Araçatuba and Bauru in the northeastern region of São Paulo; (2) the Euclides Figueiredo highway (SP 563) linking the VL endemic regions of Pereira Barreto, Ilha Solteira, and Andradina to Dracena, one of the most important cities of RNHA11, also most prevalent in VL. Secondary highways also link the RNHA11 from south to north. Two important radials highways link MS directly to the metropolitan area of São Paulo city, the capital of São Paulo state. Both Raposo Tavares (SP 270) and João Ribeiro de Barros (SP294) cross from the west to the east of RNHA11 mesoregion. In the Pontal of Paranapanema, several secondary highways link all the municipalities. Teodoro

Sampaio is connected by a network of radial and secondary highways. As previously pointed out, the Euclides Figueiredo highway (SP 563) connects the VL endemic regions of Pereira Barreto, Andradina, Dracena, Tupi Paulista, Ouro Verde, and Presidente Venceslau to Teodoro Sampaio. The Arlindo Bettio highway (SP 613) links Teodoro Sampaio to the municipality of Rosana, crossing the Paraná River, connecting the city of Nova Andradina in MS. From Teodoro Sampaio, the same highway crosses the Paranapanema River connecting to the city of Itaguagé, in the state of Paraná. The secondary vicinal highway (VSP-031; VSP-035) connects the endemic regions of Presidente Epitacio and Panorama, bordering the Paraná River to Teodoro Sampaio (Fig 3A).

Fig 3. Geographic distribution of human visceral leishmaniasis in the RNHA11 mesoregion between 2005 and 2018. The colors represent the cumulative number of cases (A). Flow and spatial dispersion of human visceral leishmaniasis from 2005 to 2018 in the RNHA11 mesoregion, and the year in which the first case was detected in each municipality (B).

The spread of human visceral leishmaniasis in RNHA11 mesoregion and Pontal of Paranapanema

Fig 3A shows that over 14 years of follow-up (2005–2018), 20 of 45 (44.4%) municipalities in RNHA11 mesoregion referred patients infected with VL. In the northern area, all the municipalities' notified cases, most of them at increased rates (17-157 cases), and Dracena (dark red), considered to be the epicenter, showed the highest rates (58–176 cases). On Pontal of Paranapanema, 8 of 32 (25.0%) municipalities registered HVL, and dispersion on the regional scale occurred in three different ways: irradiation, contiguity, and jumps (when municipalities far from the endemic region register cases of CVL or HVL). In municipalities located along the Paraná River, only one did not register cases (Fig 3A and B). Fig 3B shows that cases were found first in

Dracena in 2005, in subsequent years HVL spread to the neighboring municipalities and in ~~2009~~ reached the municipalities of Pontal of Paranapanema. Teodoro Sampaio, 133 km from the epicenter, was one of the last municipalities to register cases and HVL was detected in 2018. The first autochthonous case of HVL was identified in 2018 in the eastern area, area 1, on the outskirts of Teodoro Sampaio (Fig 2B), where a high number of cases of CVL were found throughout the period. The patient was a 48-year-old man who did not respond to treatment with meglumine antimoniate and was treated with liposomal amphotericin B (5 mg/kg/day) for 5 days with improvement in the symptoms and 50% reduction of splenomegaly.

Paraná and Paranapanema Rivers in RNHA11 and surrounding areas of Teodoro Sampaio

Fig 1A and B and Fig 3A and B show that the municipalities in the RNHA11 mesoregion are divided from MS on the western side by the Paraná River, which flows along the border of five cities. On the south side, the region is divided from Paraná state by the Paranapanema River, which flows along the border of ten municipalities. Together, the rivers support seven large lakes and a flooded area of 6175 km^2 and seven dams and bridges (Fig 1A). These bridges link the VL endemic regions of Três Lagoas in MS and Andradina in São Paulo state to the areas of the western region as well as the municipalities of Pontal of Paranapanema to the VL-free areas of Paraná state. The urban area (Fig 1Ca) is surrounded by the Devil’s Hill State Park (Fig 1Cb), 6.3 km away, and linked by a highway with the administrative offices, museums, laboratories, and environmental educational areas of the park. The municipality of Teodoro Sampaio is served by both the Paraná and Paranapanema Rivers, which surround the urban area of the city at a distance of 1 km (Fig 1 Cc).

Entomological survey of *Lu. longipalpis* in the urban area of Teodoro Sampaio

In São Paulo, from January 1997 to July 2019 sand flies, vectors of *Lu. longipalpis* was found in 203 of 645 (31.5%) of the municipalities. In RNHA11, the vector was first found in all the municipalities in the north and dispersed toward the south in the direction of the municipalities of Pontal of Paranapanema and Paraná state, and was found in 35 of 45 (77.8%) of the municipalities. In Teodoro Sampaio, they were initially found in July 2010 in area 1, sectors 2 and 4 in blocks 166 and 298, and in October 2010 in sector 1, block 75 (Table 1).

Table 1. Spatiotemporal distribution of *Lu. longipalpis* sandflies in the urban area of Teodoro Sampaio, São Paulo, Brazil.

**MUNICIPALITY OF TEODORO SAMPAIO**						
**AREA**	**SECTOR**	**BLOCK**	**DATE**	**No. OF TRAPS**	**No. OF TIMES**	**SPECIES/NUMBER**
1	2	166	22 July 2010	9	9	*Lu. longipalpis* 1M
1	4	298	27 July 2010	10	10	*Lu. longipalpis* 1M
1	1	75	25 October 2010	14	14	*Lu. longipalpis* 1M
		Total		33	33	3

Canine serological survey and spatial distribution of euthanized dogs in Teodoro Sampaio

From January 2010 to December 2018, 3749 dogs were selected for serology by the CZC and 298 animals (7.9%) were positive for CVL. Fig 2A shows the kernel spatiotemporal distribution of euthanized dogs. Of the 298 dogs that were positive, 110 (36.9%) were euthanized according to the criteria of the Brazilian Ministry of Health. In 2010 and 2011, infected dogs were identified and euthanized in sectors 1, 2, and 3 (three hotspots; dark red color). In 2012–2013, infected dogs were found in the areas surrounding the sectors where dogs positive for CVL were found in 2010–2011 (ochre color). From 2014 to 2017, positive cases reached the entire urban area, including sector 4 (apple green and green colors), and in 2018 no euthanized dogs were registered. Fig 2B shows the hotspots of infected dogs euthanized. The periphery of sector 1 showed a higher number, demonstrated by critical points hotspots, whereas sector 4 showed the lowest number. These data allow us to consider that a stronger force of infection occurs in sector 1, the main hotspot. The first case of HVL in the city was located in this sector. Several dogs were not euthanized for different reasons. Among the municipalities of RNHA11, 12 of 45 (26.7%) do not have CZC, 3 of 45 (6.7%) have a partial physical and human structure of CZC, and 30 of 45 (66.6%) have a structured CZC (Fig 4).

Fig 4. Spatial distribution of the HDI, the presence of CZCs, and populations >40,000 inhabitants in municipalities of RNHA11 mesoregion. Source: State System Data Analysis Foundation (SEADE, 2017). Base map: digital meshes of IBGE (2010). The municipalities are numbered as follows: 1, Paulicéia; 2, São João do Pau D'alho; 3, Monte Castelo; 4, Santa Mercedes; 5, Nova Guataporanga; 6, Junqueirópolis; 7, Irapuru; 8, Tupi Paulista; 9, Panorama; 10, Ouro Verde; 11, Dracena; 12, Presidente Epitácio; 13, Caiuá; 14, Presidente Venceslau; 15, Piquerobi; 16, Ribeirão dos Índios; 17, Emilianópolis;18, Flora Rica;19, Santo Expedito; 20, Alfredo Marcondes; 21, Marabá Paulista; 22, Santo Anastácio; 23, Presidente Bernardes; 24, Alvares Machado; 25, Presidente Prudente; 26, Caiabu; 27, Martinópolis; 28, Rancharia; 29, João Ramalho; 30, Quatá; 31, Rosana; 32, Euclides da Cunha Paulista; 33, Teodoro Sampaio; 34, Mirante do Paranapanema; 35, Sandovalina; 36, Tarabai; 37, Pirapozinho; 38, Estrela do Norte; 39, Narandiba; 40, Anhumas; 41, Regente Feijó; 42, Indiana; 43, Taciba; 44, Nantes; 45, Iepê.

Laboratory findings

Hematologic abnormalities are among the most common manifestations in patients infected with VL in whom pancytopenia may occur (Table 2). From 159 individuals analyzed, mild anemia occurred in 5 (3.1%) individuals, leukopenia occurred in 3 (1.9%), and thrombocytopenia occurred in 4 (2.5%). The mean values±SEM, with 95% confidence intervals (CI) were as follows: red blood cells, 4.81±0.03 million/ cells/mcL (95% CI, 4.50–4.62); hemoglobin (g/dL), 13.80±0.10 (95% CI, 13.59–14.00); hematocrit (%), 40.33±0.27 (95% CI, 39.78–40.88); mean corpuscular volume (fL), 88.49±0.33 (95% CI, 87.83–89.15); mean corpuscular hemoglobin (pg), 31.50±1.25 (95% CI, 29.02–33.99); mean corpuscular hemoglobin concentration (g/dL), 34.20±0.06 (95% CI, 34.07–34.34); platelets ($\times 10^3$/mcL), 266.359±4.740 (95% CI, 256.997–275.720). When the main changes in white blood cells were analyzed in the whole population, 17 (10.7%) showed mild leucopenia, 13 (8.1%) had mild leukocytosis, 25 (15.7.2%) had neutropenia, 41 (25.8%) had eosinophilia, 18 (11.3%) had

lymphocytosis, and 11 (6.9%) had monocytosis. Mild thrombocytopenia was present in 9 (5.6%) individuals.

Table 2. Hematologic and biochemical features recorded in 159 individuals included in the human serological survey in Teodoro Sampaio, São Paulo state, Brazil, in July 2018

PARAMETER	NUMBER (%)	MEAN±SD	95% CI	NR
Erythrocytes (million/µL)				
Men <4.5	3 (6.6)	4.207±0.1677	3.790–4.623	5.00±0.5
Women <3.8	3 (2.6)	3.537±0.1050	3.276–3.798	4.3±0.5
Hemoglobin (g/dL)				
Men <13	1 (2.2)	12.50		15.0±2.0
Women <12	9	11.48±0.4410	11.14–11.82	13.5±1.5
Hematocrit (%)				
Men <40	4 (8.8)	38.58±0.8261	37.26–39.89	45±5
Women <39	57 (50)	36.90±1.632	36.47–37.33	41±5
MCV <83 (fL)	11 (6.9)	78.84±2.599	77.09–80.58	92±9
MCH <27 (pg)	6 (3.7)	25.70±0.8809	24.78–26.62	29.5±2.5
MCHC (g/dL) <31.5	0	0		33±1.5
RDW <11.6 (fL)	20	11.30±0.2038	11.20–11.39	12.8±1.2
Platelets<150 (mm^3)	4 (2.5)	133.3±7.274	121.7–144.8	150–400
Leukocytes <4.0 (mm^3)	3 (1.8)	133.3±7.274	2.515–4.471	7.0±3.0
Neutrophils <2.0 (mm^3)	0	0		2.0–7.0
Hepatic enzymes				
AST (IU)				
Men >50	2 (4.4)	85.50±40.31	0	10–50
Women >35	8 (7.0)	50.63±6.968	44.80–56.45	7–35
ALT (IU)				
Men>50	3 (6.6)	59.33±6.807	42.42–76.24	10–50 U/L
Women >35	12 (10.5)	56.50±20.87	43.24–69.76	10–35 U/L

ALT, alanine aminotransferase; AST, aspartate aminotransferase; MCH, mean corpuscular hemoglobin; MCHC, mean corpuscular hemoglobin concentration; MCV, mean corpuscular volume; NA, not available; RDW, red cell distribution width; NR, normal range .

Epidemiologic characteristics of the study population in Teodoro Sampaio

Between January and December 2018, 159 individuals were screened. The mean age was 43.50±16.83 years (95% CI, 40.87–46.14; range, 18–89 years). The mean residence time in the city was 23.86±15.27 years (95% CI, 21.46–26.25; range, 1–68 years). There were a greater number of women 115 of 158 (72.3%) than men because there are more

women in the area where the research was carried. The age-related distribution was as follows: >18–20 years, 21(13.2%); 21–40 years, 52 (32.7%); 41–60 years, 55 (34.6%); and 61–80 years, 31 (19.5%).

The HDI in municipalities in RNHA11 mesoregion

In the municipalities in this study, the HDI varied from very low in 4 municipalities and low in 15 municipalities to high only in Presidente Prudente, the most important city in RNHA11 mesoregion. Of the areas with very low and low HDIs, 18 (40%) of the municipalities are located in the Pontal of Paranapanema region. Ribeirão Preto, connecting the Paraíba Valley to the Metropolitan region of São Paulo city, and from Campinas to Piracicaba presents an axis of great development, with the HDI varying from 0.790 to 0.826. The regions with the lowest HDI values are the Ribeira Valley, located in the south of the state, and Pontal of Paranapanema (our study area). In Teodoro Sampaio, rates vary from 0.678 to 0.715. In São Paulo state, the HDI is distributed by regions, and maps are available in Fundação Sistema Estadual de Análise de dados (SEADE), Teodoro Sampaio is surrounded by very poor municipalities, Euclides da Cunha Paulista, Marabá Paulista, and Caiuá, with rates between 0.6770 and 0.704 (Fig 4).

When the functional relationship between the number of cases of HVL per 10,000 inhabitants and the HDI of each municipality was calculated, the correlation (adjusted R value) had a value less than 10% and the *P* value was not significant, however, when municipalities that did not have cases of VL were removed and the simple linear regression technique was applied, the regression line showed a trend for lower values of VL cases per 10,000 inhabitants as the HDI increased (Fig 5).

Fig 5. Correlation between HDI and VL infection in municipalities in RNHA11 mesoregion.

Discussion

In 2005, HVL reached the municipalities in RNHA11 mesoregion, western São Paulo, and in 2009 the municipalities of Pontal of Paranapanema, a poor and asymmetric region. CVL was found in Teodoro Sampaio, considered the capital of Pontal of Paranapanema, in 2010 and HVL in 2018. In 2019, 19 of 45 (42.2%) municipalities in RNHA11 recorded cases. A complex array of socioeconomic and environmental factors may be fueling the epidemic and sustaining endemic transmission of VL (Fig 6).

Fig 6. Environmental and socioeconomic structures related to the transmission of visceral leishmaniasis (VL) in the Regional Network for Health Assistance11 (RNHA11) mesoregion (Rede Regional de Assistência a Saúde), São Paulo, Brazil. In the last decades, social, economic, and environmental structures have been changing the landscape and the way of life of people in the region. Among the social and economic factors are (1) low HDI due to deficiencies in sanitation, education and low family income; (2) the extensive number of roads linking small- and middle-sized cities from endemic to non-endemic areas; (3) cities without a Center of Zoonosis Control or ineffective control of canine visceral leishmaniasis. Among the environmental changes are (1) the construction of several artificial lakes and dams in the Paraná and Paranapanema Rivers; (2) extensive areas of flooding and increasing temperature and humidity in surrounding lakes; (3) extensive deforestation in the Tropical Forest and reduction of the forested area to 12%. Taken together, these factors have certainly contributed to the increasing number of infected dogs, the proliferation of vectors, and the spread of human visceral leishmaniasis in the RNHA11 mesoregion of São Paulo.

In the last few years, the question from where and how VL reached the western region of São Paulo has been highlighted. Epidemiologic findings demonstrated that the disease crossed the border of Bolivia and was found in the cities of Corumbá and Ladário in MS, on the western side of the state, expanded to its capital, Campo Grande, crossed the entire state and reached Três Lagoas, on the border of São Paulo. The

dissemination followed the cities along with the construction of the Novoeste railway (1909–1952), a federal highway (BR-262) completed in 1980, and recently the construction of a pipeline (1998). These constructions linked the high VL endemic regions of Corumbá, Campo Grande, and Três Lagoas to the western region of São Paulo. During these construction projects (1952–1998), thousands of families, workers, infected dogs, and vectors migrated, spreading the disease. In 1998 and 1999, infected dogs and humans were first found in Araçatuba, a city harboring the railway, the highway, and the pipeline [5–14]. Recently, using a multi-locus microsatellite with input directly from clinical samples of infected dog tissues obtained in RNHA11 mesoregion, it was demonstrated that the genetic structure of *Leishmania infantum*, the parasitic agent of VL in the Americas, was similar to that in the dogs found in MS, reinforcing the origin by epidemiologic findings [15]. Once present in the western region, VL has been spreading widely in different directions and in different ways. The first phlebotomine outbreak was found in the microregion of Dracena, a city located in the north and considered the epicenter. One year later, vectors were found in Tupi Paulista and other adjacent municipalities, which suggested the existence of a possible route of dispersion by irradiation or by contiguity. However, vectors of CVL and HVL were registered in the region far from the epicenter, suggesting dispersion by jumps and toward the municipalities of the south, reaching the municipalities of Pontal of Paranapanema (Fig 3A and B). To date, the western region has harbored 537 of 3042 (17.6%) of individuals infected with VL in São Paulo [4].

It has been suggested that the presence of environmental risk factors in the region, including deforestation, big rivers, and artificial lakes, induces climate change. Socioeconomic risk factors, including the highways network linking endemic to non-endemic cities, small cities without CZC, and low HDI/education/sanitation/low family

income make it difficult to undertake surveillance and control of vectors and the correct handling of infected dogs. These factors may favor the biological cycle of the disease, increasing the presence of *L. infantum* parasites, *Lu. longipalpis* vectors, and infected *Canis familiaris*, which in turn infect humans, the definitive host. The role of the environment and urban ecosystems in the tracks of the spread of vectors of yellow fever virus in Brazil was demonstrated recently [16]. The RNHA11 mesoregion harbors an extensive highway network (2382 km), linking endemic regions to small- and middle-sized cities. We suggest that the main axis adding to VL dispersion is the Euclides Figueiredo highway (SP562). Starting in Ilha Solteira outside RNHA11, the highway goes down toward the cities of Andradina and Dracena and toward the municipalities of Pontal of Paranapanema, crossing Teodoro Sampaio at the border of Paraná state. Nevertheless, dissemination may also occur by a secondary axis. The role of SPV031 and SPV035, starting near Brasilândia, an endemic region in MS, and following the Paraná River through small-sized cities and ending in Teodoro Sampaio must also be considered. All the municipalities crossed by these highways became endemic areas of VL. Built on the edge of the Paraná River and along the highway, thousands of small leisure properties have been constructed. They support a daily flow of people, cars, animals, and goods. Furthermore, the proliferation of vectors could be aggravated by the extensive watershed flowing into the Paraná and Paranapanema Rivers, with nine large lakes and a flooded area of 6174.53 km^2, supporting hydroelectric plants and bridges. Porto Primavera dam in Paraná River has an artificial lake with a flooded area of 2.250 km^2 and divides the municipalities of RNHA11mesoregion and MS. The lake has three bridges linking the VL endemic region of MS to the municipalities of the western region. Dividing São Paulo and Paraná state, the Paranapanema River flows on the border of ten municipalities and the lakes support three dams with a flooded area of

2.906 km^2. Four bridges over the artificial lakes link municipalities in RNHA11mesoregion with increasing rates of VL to the VL-free areas of Paraná state. We suggest that municipalities of Paraná state are under pressure with regard to vectors and infected dogs that may spread the infection. As far as we know, there is no investigation on VL in these areas. The role of highways in carrying vectors, parasites, bacteria, and viruses in the spread of diseases is well known, particularly in the spread of VL from endemic to non-endemic areas [5,14,17-18]. Recently, the route of SARS-CoV-2, responsible for the transmission of COVID-19, from the epicenter, São Paulo, to the municipalities of RNHA11 mesoregion was demonstrated through extensive radial and transverse highways.

In Brazil, the construction of dams is a reality in hundreds of rivers, and artificial lakes are used for energy production. Artificial lakes modify the climate of their surrounding areas as well as the environment within which the lake interacts. In the region, in cities bordering the lakes, higher temperatures of 2°C increased humidity rates were found compared with municipalities far from the lakes. In RNHA11, the Tropical Forest was devastated with only 12.3% remaining, including the Devil's Hill State Park and small fragments [19]. Currently, the land is occupied by pastures, sugar cane monoculture, grain agriculture, and rural settlements [20]. For vector-borne diseases, deforestation increases the abundance of open habitats of some vectors and decreases host diversity and the prevalence of pathogens may increase. According to the 2018 United Nations Organization Biodiversity Report, deforestation is one of the main causes of transmission of diseases such as malaria, dengue, yellow fever, and Zika virus; cases of these diseases have doubled in the last three decades. Furthermore, climate change can lead to the emergence of new diseases and the return and spread of restricted diseases or even those already controlled [21]. In Pontal of Paranapanema, in

the 1950s, vectors of cutaneous leishmaniasis were restricted to the Devil Hill State Park. Currently, they are found in forest fragments and the disease is endemic throughout the region [22,23]. In traps located in the surrounding areas, 1638 sand flies involved in the transmission of leishmaniasis were captured in 8 hours [24]. In different rural settlements located in the municipality of Euclides da Cunha Paulista, alarming rates of Triatominae, the vector of Chagas disease, as well as infected individuals were found [25]. In addition to parasites, diseases transmitted by viruses and bacteria have been increasing in the region. In the 1980s, the presence of *Aedes aegypti*, the vector of dengue virus, was seasonal with higher levels of infected patients during the summer and very low levels in the winter. Now, the vector has no seasonality and in 2020 we are facing an outbreak of dengue in RNHA11 mesoregion. Furthermore, an increasing number of patients diagnosed with the harmful Brazilian spotted fever transmitted by ticks and caused by *Rickettsia rickettsii*, coming from areas surrounding artificial lakes were referred to the Regional Hospital of Presidente Prudente [26].

The burden of socioeconomic structures of people living in Teodoro Sampaio and surrounding areas imposes health care inequalities, poor housing conditions, lower income, less education, or lower occupational skill levels, and the population tend to be less healthy than those who experience higher levels of social conditions in other areas of São Paulo state. In contrast to the dynamic economic situation in the rest of the state, sustained growth of Pontal of Paranapanema is hampered by ongoing conflicts between landowners and the Landless Movement, organized to fight against inequalities in inland distribution, agrarian reform, and social changes in the country. Pontal of Paranapanema has a higher concentration of rural settlements in São Paulo state and one of the highest in Brazil [20,27]. In addition, the HDI of Teodoro Sampaio and surrounding municipalities, Marabá Paulista and Euclides da Cunha Paulista, are among

the lowest in the state. Supporting the data, demographic, environmental, and behavioral characteristics of inhabitants of rural settlements have been determined. In Teodoro Sampaio, only 7.1% had a high school education, 11.9% lacked elementary instruction, and 77.3% had completed primary school. The income varied from less than 100 US$ (91.6%) to about 170 US$ (8.4%) per month. Similar rates were found in a rural settlement of Mirante of Paranapanema [20,27]. In a recent study, increasing rates of congenital syphilis were found in municipalities of Pontal of Paranapanema [28]. Among the risk factors found in these studies that increase vulnerability to infectious diseases in the region are low family income, illiteracy, inadequate sanitation, and health care conditions [20,28-29]. Although a direct association between HDI and the presence of VL in municipalities of RNHA11 mesoregion was not found, linear regression showed a tendency of a lower number of infected individuals in municipalities with higher HDI. In addition, low income and lack of formal jobs, low industrialization, and sugarcane monoculture with increasing use of machines, severe unemployment and exodus of young people to bigger cities certainly contributed to the region becoming the new focus of VL in Brazil. Globally and countrywide, VL is considered a neglected disease that affects the poorest of the poor [29]. This phenomenon was pinpointed on the periphery of sector 1 of Teodoro Sampaio, a very poor area that had a higher number of infected dogs, demonstrated by critical point hotspots. The first human case of VL in the city was diagnosed in this sector.

From 2010 to 2018, 298 (7.9%) dogs were found to be positive for CVL and 110 (36.9%) were euthanized. They were first found in sectors 1, 2, and 3 on the periphery, and in subsequent years, the hot spots spread to all urban areas. However, seropositive dogs were found and euthanized in sector 4 only in 2015, and few cases of CVL were diagnosed. This is a middle- and upper-income residential area and in these settings,

dogs are confined to their owner’s house, with pet care services and good health care [7]. Most of the cities of Pontal of Paranapanema do not have CZC. In these settings, lack of sanitation, sites of irregular domestic waste disposal and increased numbers of stray dogs and cats are harmful risk factors for the dispersion of VL and other zoonotic diseases such as toxoplasmosis and toxocariasis [20]. It has been demonstrated that in RNHA11 mesoregion, the smaller municipalities face greater difficulties, both in the implementation of zoonosis services and surveillance control as well as their maintenance. Difficulties were directly associated with socioeconomic status [13]. In Teodoro Sampaio, low levels of infected dogs were euthanized (36.9%). Similar to other cities of Pontal of Paranapanema, Teodoro Sampaio is surrounded by a high number of rural settlements [20,27]. Throughout the region, it is anecdotal that owners of dogs infected with VL hide their animals in these settings. In RASS11 mesoregion, VL was characterized as being found in an urban area of the cities. However, dogs with VL that are hidden by their owners may be disseminating the disease to rural areas. Although controversial, euthanasia is one of the most important actions supported by Brazilian Visceral Leishmaniasis Surveillance and Control Programme in reducing the prevalence of *L. infantum* infection countrywide [30]. It has been shown that human epidemics of VL are usually preceded by or concomitant with CVL [7]. However, in Teodoro Sampaio, a diagnosis lag of 9 years between finding vectors, CVL, and HVL was found. Up to June 2020, only one individual had been registered. One possible explanation is that the city is located in the neighborhood of the Devil’s Hill State Park, an endemic region for cutaneous leishmaniasis (CL) vectors [23,24], and the municipality is considered endemic for CL. There is some evidence that vectors have been present in the urban area for many years and a superposition CL/VL is occurring and a cross-reaction may be on course, protecting people from VL infection [31].

Supporting these findings, a VL serological survey of 159 individuals living in the urban area was carried out and only 2 (1.26%) individuals were positive by ELISA, with a probable false positive and/or cross-reaction. In addition, the mean residence times in the city was 23.86±15.27 years, increasing the possibility of these individuals being immunized against CL. Although VL-infected individuals do not have hallmark signs and symptoms, severe pancytopenia (thrombocytopenia, leukopenia, and anemia) and increased levels of hepatic enzymes may be considered in the differential diagnosis in VL endemic areas. In the population analyzed in this study, no laboratory findings symptomatic of chronic VL were found.

The way this disease is related to a combination of social and economic factors and climate change can be generalized to other regions of the country and the world. In Santarém, a city in the Amazon region at the confluence of the Tapajós and Amazon Rivers, VL is considered endemic and its rates are increasing [32]. Daily, small and large boats and canoes arrive and leave the city, transporting people, goods, and animals to other small towns, villages, and islands. The situation is similar to the study region, but with rivers instead of roads serving as paths for the dissemination of VL and other vector-borne diseases from endemic to non-endemic areas. In Morocco, there has been progressive expansion of the range of distribution of VL with difficulties in control of *Phlebotomine* sand fly vectors, inefficient actions against the CVL population, and unfavorable socioeconomic factors [33].

Different shortcomings should be considered in this study. The mean age of the population screened for VL was 43.50 years, and 72.33% were women. It is well known that VL affects mainly children and old people. Thus, the population analyzed is not well representative of the disease [34]. Municipal surveillance control programs of VCL were discontinued and there were difficulties in obtaining current records with a more

accurate surveillance and response system to apply geospatial methods. A changing landscape is occurring in RAAS11 overwhelmingly in Pontal of Paranapanema, where pasture and familial agricultural areas are being replaced by sugar cane monoculture. We are not sure if this new scenario favors or discourage the spread of vectors and the dissemination of VL in the region.

Conclusion

In RNHA11 mesoregion, VL is spreading with a progressive extension of the range of distribution, from the north, the epicenter, to the south, from endemic to non-endemic areas. Environmental, social, and economic factors may make actions against CVL and geographic control of sand fly vectors difficult, fueling the epidemic and sustaining the endemic transmission of the disease. These findings further highlight the asymmetry of the western region compared with other regions of São Paulo state and add new knowledge on the spread of VL, contributing to the study of a neglected disease in a ~~neglected~~ region of São Paulo, Brazil.

Author Contributions

Conceptualization: Luiz Euribel Prestes-Carneiro

Data curation: Luiz Euribel Prestes-Carneiro

Formal analysis: Elivelton Silva Fonseca, Lourdes Aparecida Zampieri D'Andrea

Funding acquisition: Luiz Euribel Prestes-Carneiro

Investigation: Regiane Soares Santana, Karina Briguenti Souza, Cristiane Oliveira Andrade, Lourdes Aparecida Zampieri D'Andrea, Ivete Rocha Anjolete

Methodology: Lourdes Aparecida Zampieri D'Andrea, Ivete Rocha Anjolete, Elivelton Silva Fonseca, Francisco Assis Silva, Edilson Ferreira Flores, Marcia Kaihara Meidas

Project administration: Luiz Euribel Prestes-Carneiro[1]

Supervision: Regiane Soares Santana

Validation: Luiz Euribel Prestes-Carneiro[1]

Visualization: Elivelton Silva Fonseca, Francisco Assis Silva, Edilson Ferreira Flores, Fernanda Lussari

Writing – original draft: Luiz Euribel Prestes-Carneiro.

Writing – review & editing: , Luiz Euribel Prestes-Carneiro, Regiane Soares Santana, Karina Briguenti Souza, Fernanda Lussari, Elivelton Silva Fonseca, Cristiane Oliveira Andrade, Lourdes Aparecida Zampieri D'Andrea, Francisco Assis Silva, Edilson Ferreira Flores, Ivete Rocha Anjolete

References

1. World Health Organization. Epidemiological situation. Burden and distribution of leishmaniasis. Available from: https://www.who.int/leishmaniasis/burden/en/..
2. Pan American Health Organization. Leishmaniasis: Epidemiological Report in the Americas, March 2019. Available from: https://iris.paho.org/handle/10665.2/50505
3. Ministério da Saúde (BR). Secretaria de Vigilância em Saúde. Vigilância em saúde no Brasil 2003|2019: da criação da Secretaria de Vigilância em Saúde aos dias atuais. Bol Epidemiol. Available from: http://www.rets.epsjv.fiocruz.br/biblioteca/vigilancia-em-saude-no-brasil-20032019-da-criacao-da-secretaria-de-vigilancia-em-saude
4. Centro de Vigilância Epidemiológica "Prof. Alexandre Vranjac". Leishmaniose visceral. Available from: http://www.saude.sp.gov.br/cve-centro-de-vigilancia-epidemiologica-prof.-alexandre-vranjac/areas-de-vigilancia/doencas-de-transmissao-por-vetores-e-zoonoses/agravos/leishmaniose-visceral/
5. Cardim MF, Rodas LA, Dibo MR, Guirado MM, Oliveira AM et al. Introduction and expansion of human American visceral leishmaniasis in the state of Sao Paulo, Brazil, 1999-2011. Rev Saude Pública. 2013; 47(4): 691-700. doi:10.1590/S0034-8910.2013047004454.

6. World Health Organization. Leishmaniasis: Magnitude of the problem. Available from: https://www.who.int/leishmaniasis/burden/magnitude/burden_magnitude/en/

7. Prestes-Carneiro LE, Daniel LAF, Almeida LC, D'Andrea LZ, Vieira AG. Spatiotemporal analysis and environmental risk factors of visceral leishmaniasis in an urban setting in São Paulo State, Brazil. Parasit Vectors. 2019; 12(1): 251. doi:10.1186/s13071-019-3496-6. PMID: 31113445

8. Boton Pereira DH, Primo LS, Pelizari G, Flores E, Moraes-Vasconcelos D et al. Primary Immunodeficiencies in a Mesoregion of São Paulo, Brazil: Epidemiologic, Clinical, and Geospatial Approach. Front Immunol. 2020; 11: 862. doi:10.3389/fimmu.2020.00862. PMID: 32477349

9. Alves MCGP, Matos MR, Reichmann ML, Dominguez MH. Estimation of the dog and cat population in the State of São Paulo. Rev. Saúde Pública, 2005; 39(6): 891-897. DOI: *10.1590/s0034-89102005000600004*. PMID: 16341397

10. D'Andrea LA, Fonseca ES, Prestes-Carneiro LE, Guimarães RB, Yamashita RC et al. The shadows of a ghost: a survey of canine leishmaniasis in Presidente Prudente and its spatial dispersion in the western region of São Paulo state, an emerging focus of visceral leishmaniasis in Brazil. BMC Vet Res. 2015; 11: 273. doi:10.1186/s12917-015-0583-6. PMID: 26503368

11. Centro de Vigilância Epidemiológica (Manual de vigilância e controle da leishmaniose visceral Americana do Estado de São Paulo. 2006. Available from: https://pesquisa.bvsalud.org/portal/resource/pt/lis-LISBR1.1-22088

12. Galati EAB. Morfologia e taxonomia. In: Rangel EF, Lainson R, editors. Flebotomíneos do Brasil. Rio de Janeiro: Fundação Oswaldo Cruz; 2003. p. 23-52

13. Pereira de Souza C, Ferreira da Silva P, de Carvalho Moreno M, Aparecida Zampieri D' Andrea L. Serviços de zoonoses e o seu papel na vigilância em saúde para leishmaniose visceral. Colloquium Vitae. 2019; 11(1): 24-32.

14. Correa Antonialli SA, Torres TG, Paranhos Filho AC, Tolezano JE. Spatial analysis of American Visceral Leishmaniasis in Mato Grosso do Sul State, Central Brazil. J Infect. 2007; 54(5): 509-514. doi: 10.1016/j.jinf.2006.08.004. PMID: 16979241

15. Motoie G, Ferreira GE, Cupolillo E, Canavez F, Pereira-Chioccola VL. Spatial distribution and population genetics of Leishmania infantum genotypes in São Paulo State, Brazil, employing multilocus microsatellite typing directly in dog infected tissues. Infect Genet Evol. 2013; 18: 48-59. doi:10.1016/j.meegid.2013.04.031 PMID: 23665466

16. Possas C, Lourenço-de-Oliveira R, Tauil PL, Pinheiro FP, Pissinatti A et al. Yellow fever outbreak in Brazil: the puzzle of rapid viral spread and challenges for immunisation. Mem Inst Oswaldo Cruz. 2018; 113(10): e180278. doi:10.1590/0074-02760180278 PMID: 30427974.

17. Prestes-Carneiro LE, Spir PRN, Fontanesi M, Garcia KGP, Assis FA et al. Unusual manifestations of visceral leishmaniasis in children: a case series and its spatial dispersion in the western region of São Paulo state, Brazil. BMC Infect Dis. 2019; 19(1): 70. doi:10.1186/s12879-018-3652-1 PMID: 30658589

18. Pasquali AKS, Baggio RA, Boeger WA, Gonzáles-Britas N, Guedes DC, et al. Dispersion of Leishmania (Leishmania) infantum in central-southern Brazil: Evidence from an integrative approach. PLoS Negl Trop Dis. 2019; 13(8): e0007639. doi:10.1371/journal.pntd.0007639 PMID: 31465422

19. Ministério do Meio Ambiente, Secretaria de Biodiversidade e Florestas, Diretoria do Programa Nacional de Conservação da Biodiversidade. Cem Anos de Devastação Revisitada 30 Anos Depois. Available from: file:///D:/User/Downloads/cem_anos_de_devastacao_2005.pdf

20. Prestes-Carneiro LE, Rubinsky-Elefant G, Ferreira AW, Araujo PR, Troiani C et al. Seroprevalence of toxoplasmosis, toxocariasis and cysticercosis in a rural settlement, São Paulo State, Brazil. Pathog Glob Health. 2013; 107(2): 88-95. doi:10.1179/2047773213Y.0000000079 PMID: 23683335

21. Intergovernmental Science-Policy Platform on Biodiversity and Ecosystem Services. Regional Assessment Report on Biodiversity and Ecosystem Services for the Americas. Available at: https://ipbes.net/assessment-reports/americas

22. Cruz MF, Galati EA, Cruz CF. Ecological aspects of the sandfly fauna (Diptera, Psychodidae) in an American cutaneous leishmaniasis endemic area under the influence of hydroelectric plants in Paranapanema river, State of Paraná, Brazil. Rev Soc Bras Med Trop. 2012; 45(4): 430-436. doi:10.1590/s0037-86822012000400003 PMID: 22930040

23. Alessi CA, Galati EA, Alves JR, Corbett CE. American cutaneous leishmaniasis in the Pontal of Paranapanema - SP, Brazil: ecological and entomological aspects. Rev Inst Med Trop Sao Paulo. 2009; 51(5): 277-282. doi:10.1590/s0036-46652009000500008 PMID: 19893981

24. Condino ML, Sampaio SM, Henriques LF, Galati EA, Wanderley DM, Corrêa FM. American cutaneous leishmaniasis: sandflies from the transmission area in the town of Teodoro Sampaio, the southeastern region of São Paulo state, Brazil. Rev Soc

Bras Med Trop. 1998; 31(4): 355-360. doi:10.1590/s0037-86821998000400004 PMID: 9662962

25. Silva RA, Goldenberg P. Chagas' disease in Porto Letícia, São Paulo: a comparative study in the Pontal the Paranapanema. Rev Soc Bras Med Trop. 2008; 41(6): 621-627. doi:10.1590/s0037-86822008000600013 PMID: 19142442

26. Oliveira SC, Maziero LP, Oliveira MLV, Pinto LB. Assessment of credit restrictions to farmers in rural settlements´in the western region of the state of São Paulo, Brazil. G&DR. 2016; 12(2): 70-90.

27. Prestes-Carneiro LE, Santarém V, Zago SC, Miguel NA, Zambelli SF et al. Sero-epidemiology of toxocariasis in a rural settlement in São Paulo state, Brazil. Ann Trop Med Parasitol. 2008; 102(4): 347-356. doi:10.1179/136485908X278801 PMID: 18510815

28. Marinho de Souza J, Giuffrida R, Ramos APM, Morceli G, Coelho CH et al. Mother-to-child transmission and gestational syphilis: Spatial-temporal epidemiology and demographics in a Brazilian region. PLoS Negl Trop Dis. 2019; 13(2): e0007122. doi:10.1371/journal.pntd.0007122 PMID: 30789909

29. Alvar J, Yactayo S, Bern C. Leishmaniasis and poverty. Trends Parasitol. 2006; 22(12): 552-557. doi:10.1016/j.pt.2006.09.004 PMID: 17023215

30. da Rocha ICM, Dos Santos LHM, Coura-Vital W, Cunha GMR, Magalhães FC et al. Effectiveness of the Brazilian Visceral Leishmaniasis Surveillance and Control Programme in reducing the prevalence and incidence of Leishmania infantum

infection. Parasit Vectors. 2018; 11(1): 586. doi:10.1186/s13071-018-3166-0 PMID: 30419944

31. Fonseca ES, D'Andrea LA, Taniguchi HH, Hiramoto RM, Tolezano JE et al. Spatial epidemiology of American cutaneous leishmaniasis in a municipality of west São Paulo State, Brazil. J Vector Borne Dis. 2014; 51(4): 271-275. PMID: 25540957

32. Braga RR, Lainson R, Shaw JJ, Ryan L, Silveira FT. Leishmaniasis in Brazil. XXII: Characterization of Leishmania from man, dogs and the sandfly Lutzomyia longipalpis (Lutz & Neiva, 1912) isolated during an outbreak of visceral leishmaniasis in Santarém, Pará State. Trans R Soc Trop Med Hyg. 1986; 80(1): 143-145. doi:10.1016/0035-9203(86)90214-2

33. Mniouil M, Fellah H, Amarir F, Et-Touys, Bekhti K et al. Epidemiological characteristics of visceral leishmaniasis in Morocco (1990-2014): an update. Acta Trop. 2017; 170: 169-177 doi:10.1016/j.actatropica.2016.10.016 PMID: 27794489

34. Naufal Spir PR, Prestes-Carneiro LE, Fonseca ES, Dayse A, Giuffrida R et al. Clinical characteristics and spatial distribution of Visceral leishmaniasis in children in São Paulo state: an emerging focus of Visceral leishmaniasis in Brazil. Pathog Glob Health. 2017; 111(2): 91-97 doi:10.1080/20477724.2017.1289666 PMID: 28221822

The manuscript was edited by a native speaker: authorserv@gmail.com; Invoice no: 200103

Figure 1 Click here to access/download;Figure;Figure 1 Road Network.tif

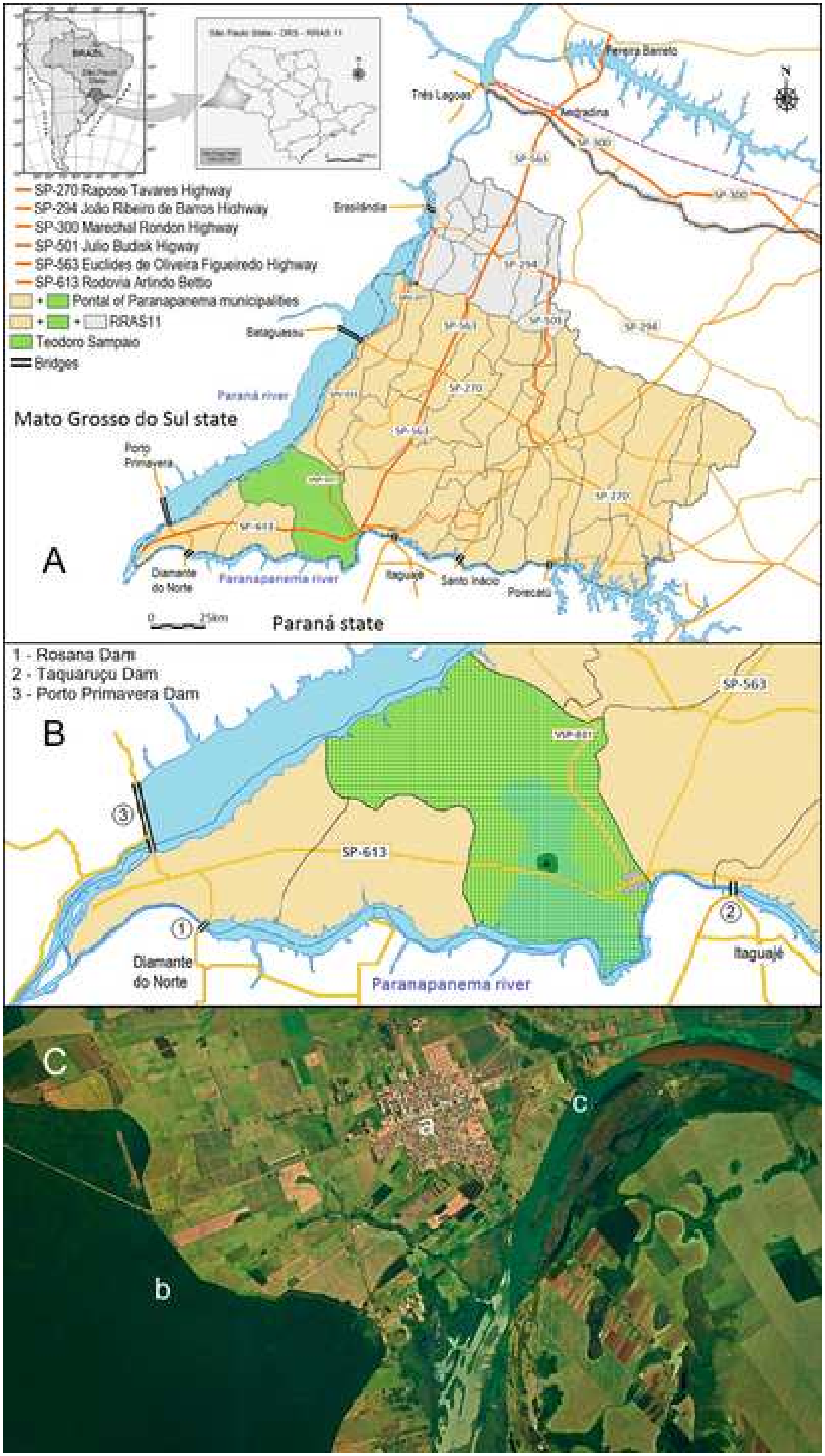

Figure 2

Click here to access/download;Figure;Figure 2 Kernel.tif

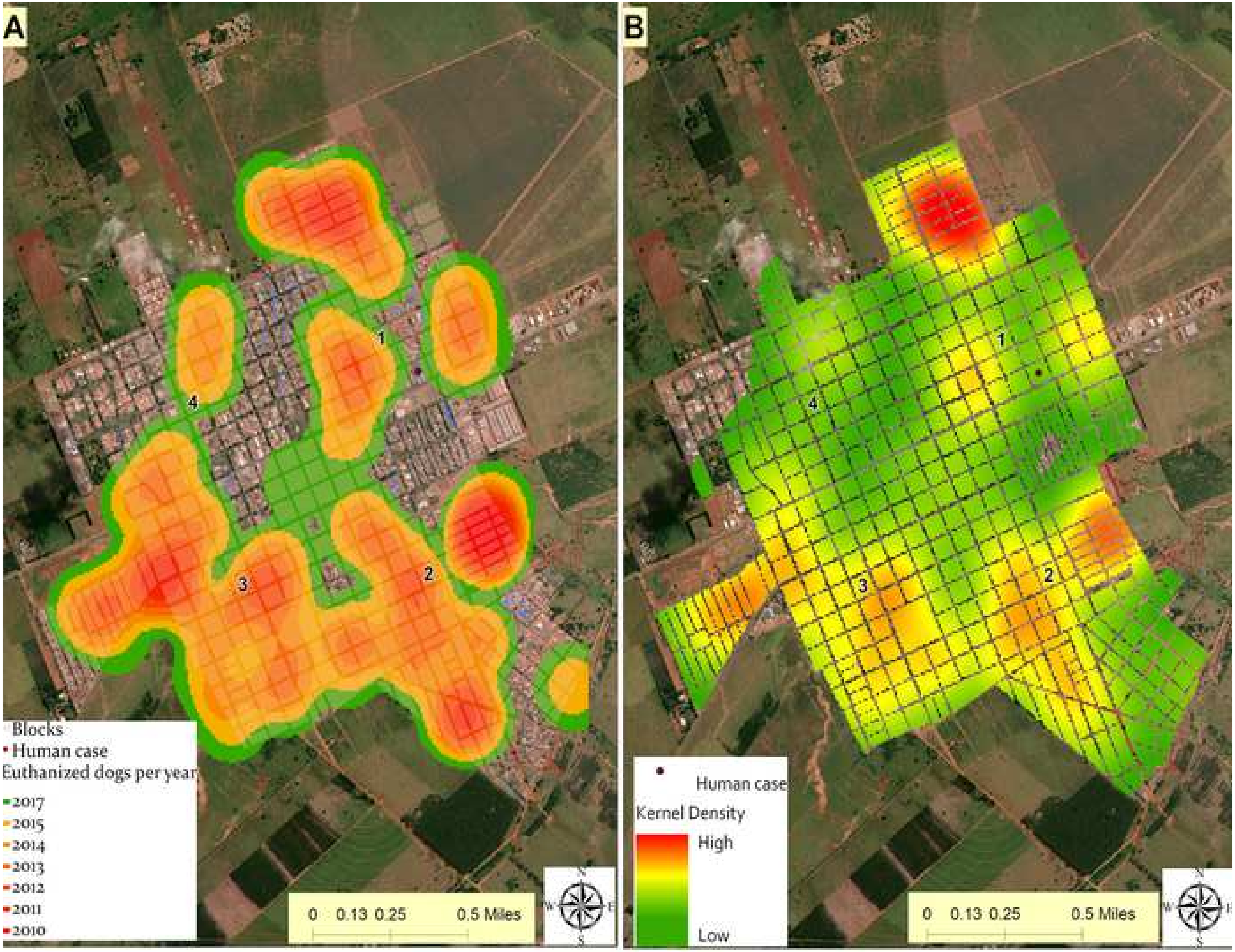

Figure 3 Flow of VL 10 MB

Click here to access/download;Figure;Figure 3 Flow of VL__300dpi 10 MB.tiff

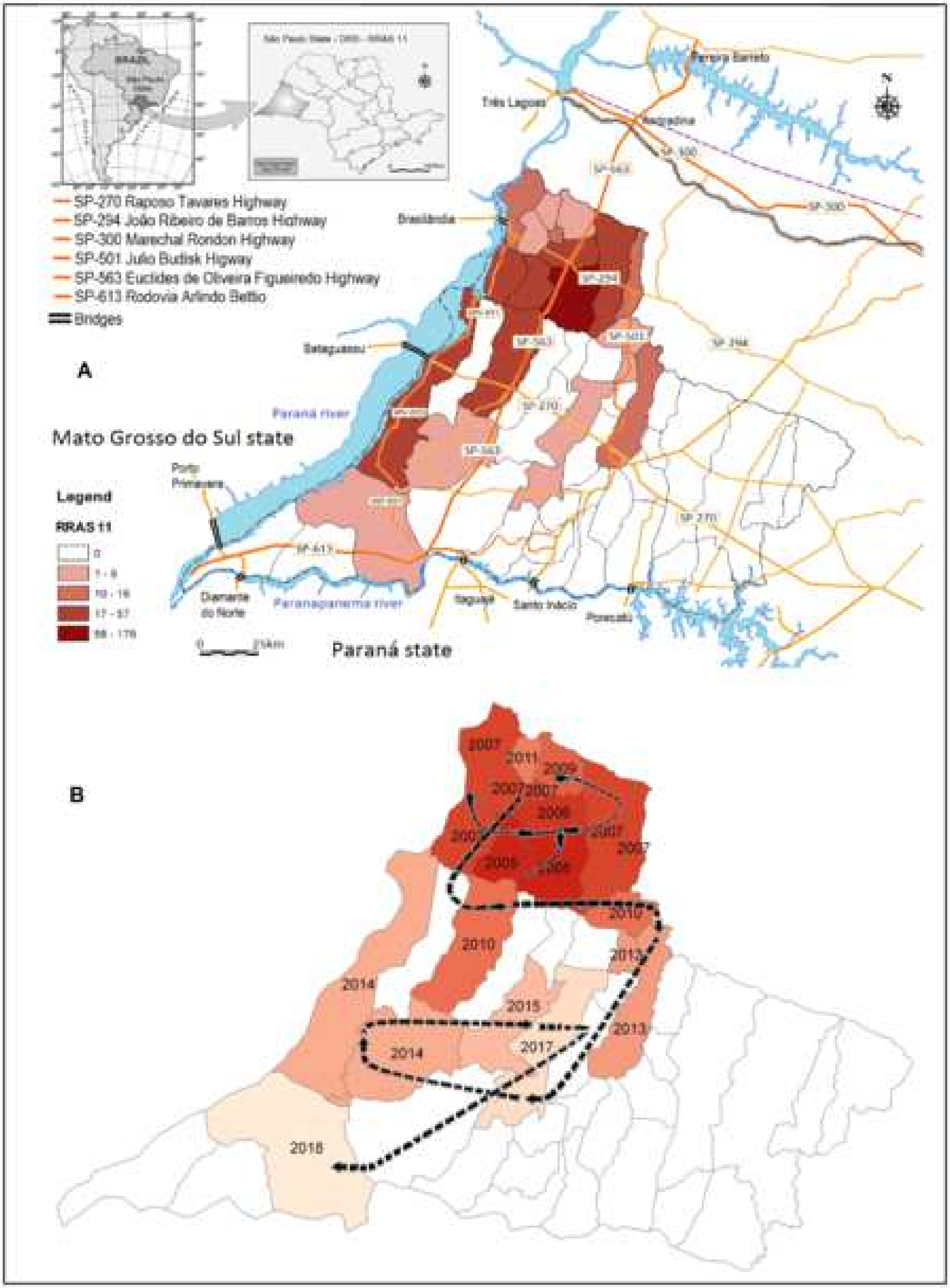

Figure 4

Click here to access/download;Figure;Figure 4 HDI.tif

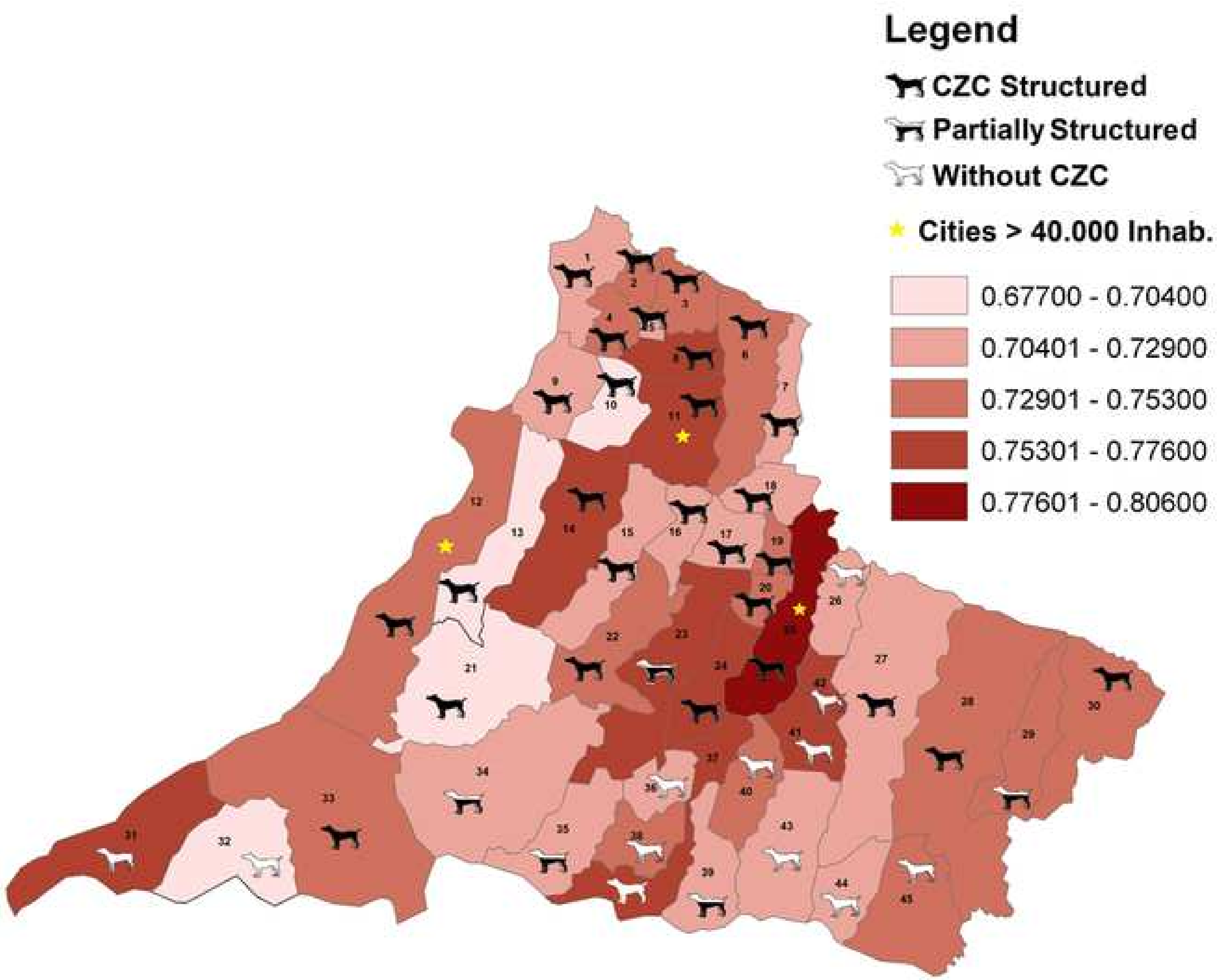

Click here to access/download;Figure;Figure 5 HDI vs VL.tif

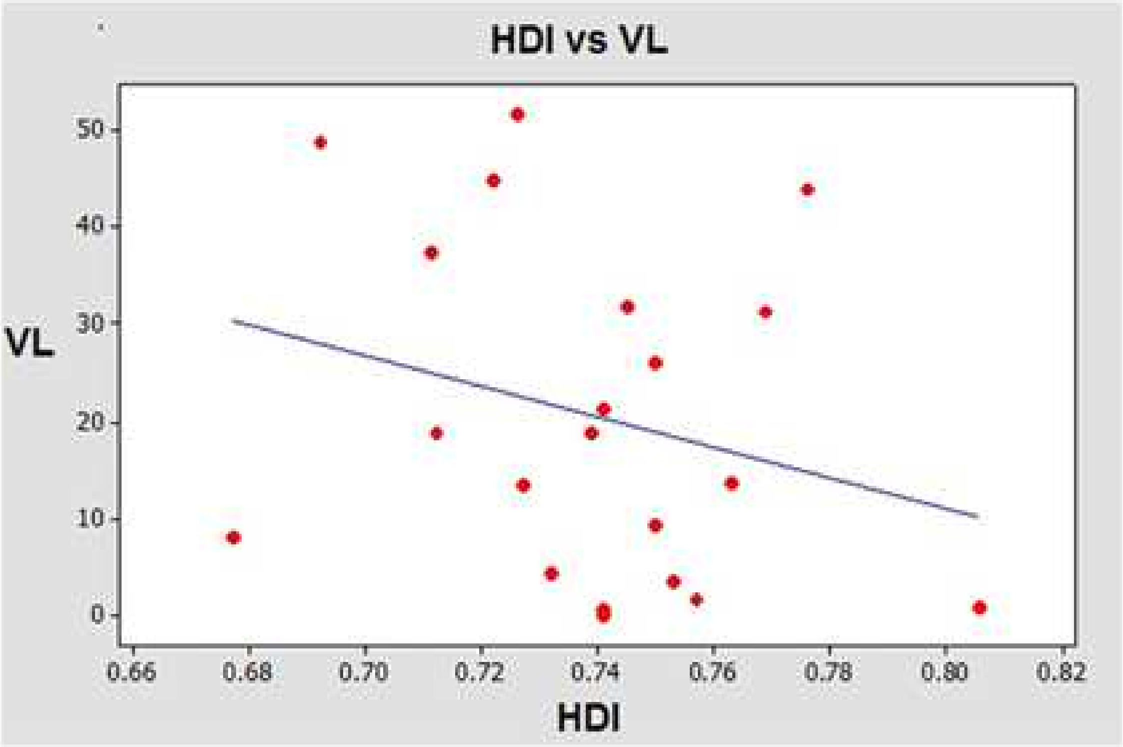

Figure 6 Environment 4.3 MB

Click here to access/download;Figure;Figure 6 Environment_300dpi 4.3 MB.tiff

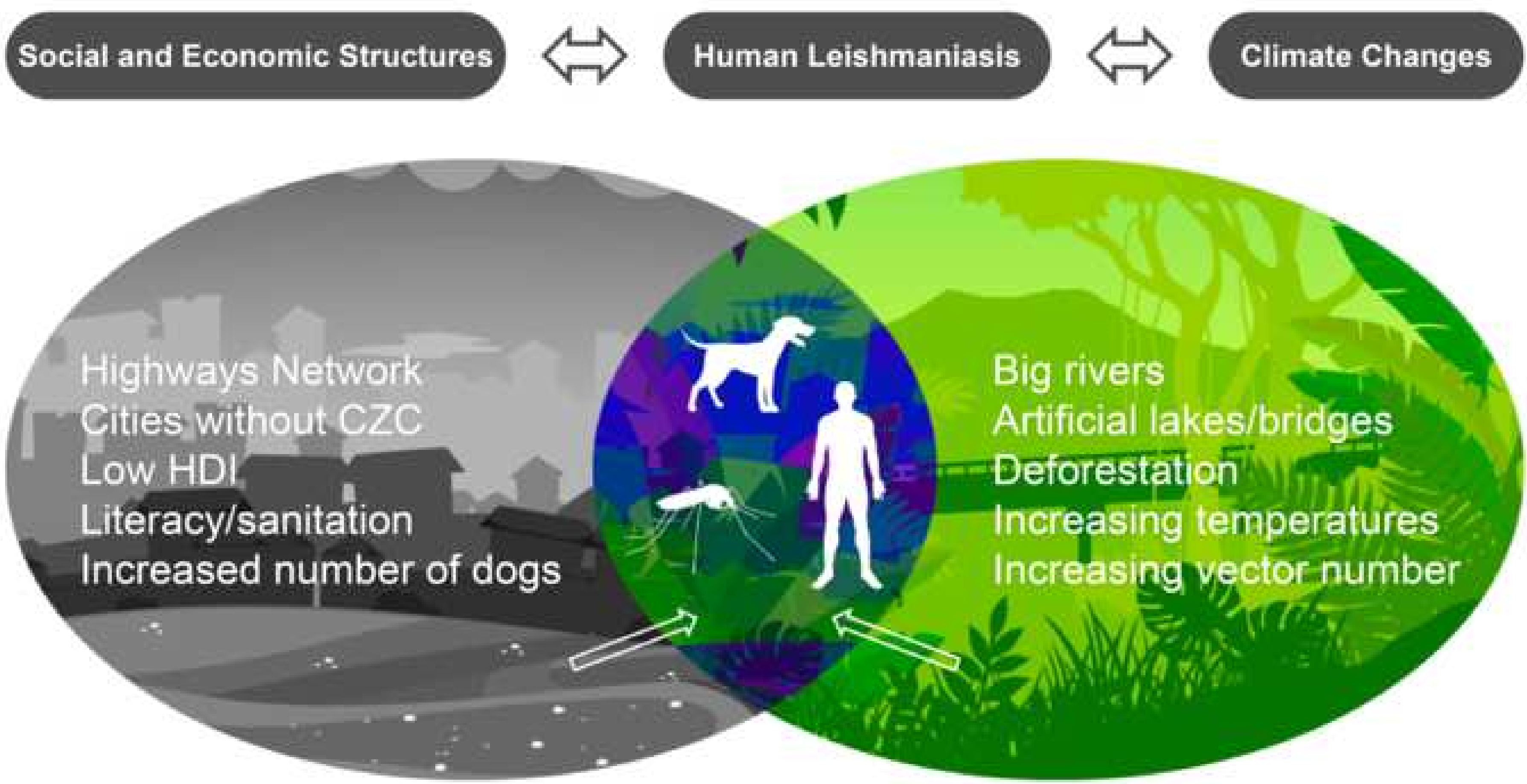

Supplement: Attachment — Submitted filename: PNTD-0- 20-01367_reviewer 12122020 11.pdf [file pntd.0009411.s002.pdf]
