# Supplementary material for: Cases and distribution of visceral leishmaniasis in western São Paulo: A neglected disease in this region of Brazil
Source: PLoS Negl Trop Dis. 2021 Jun 15;15(6):e0009411. doi: 10.1371/journal.pntd.0009411 (PMC8232419; doi:10.1371/journal.pntd.0009411)
Supplement: Attachment — Submitted filename: Revisado PNTD-D-20-01367_R12.pdf [file pntd.0009411.s004.pdf]

PLOS Neglected Tropical Diseases

Cases and distribution of visceral leishmaniasis in western São Paulo: a neglected disease in this region of Brazil

--Manuscript Draft--

**Manuscript Number:**	PNTD-D-20-01367R1
**Full Title:**	Cases and distribution of visceral leishmaniasis in western São Paulo: a neglected disease in this region of Brazil
**Short Title:**	Visceral leishmaniais in western São Paulo
**Article Type:**	Research Article
**Keywords:**	Entomological survey; Canine visceral leishmaniasis; Environmental risk factors; Socioeconomic risk factors, Spatiotemporal epidemiology
**Abstract:**	Visceral leishmaniasis (VL) is one of the most prevalent parasitic diseases worldwide. In 2019, 97% of the total numbers of cases in Latin America were reported in Brazil. In São Paulo state, currently 17.6% of infected individuals live in the western region. To study this neglected disease on a regional scale, we describe the spread of VL in 45 municipalities of the Regional Network for Health Assistance11(RNHA11). Environmental, human VL (HVL), and canine VL (CVL) cases, Human Development Index, and Lutzomyia longipalpis databases were obtained from public agencies. Global Moran I index and local indicators of spatial association (LISA) statistics were used to identify spatial autocorrelation and to generate maps for the identification of VL clusters. On a local scale, we determined the spread of VL in the city of Teodoro Sampaio, part of the Pontal of Paranapanema. In Teodoro Sampaio, monthly peri-domicile sand fly collection; ELISA, IFAT and Rapid Test serological CVL; and ELISA human HVL serum surveys were carried out. In RNHA11 from 2000 to 2018, Lu. longipalpis was found in 77.8%, CVL in 69%, and HVL in 42.2% of the 45 municipalities, and 537 individuals were notified with HVL. Dispersion occurred from the epicenter in the north to Teodoro Sampaio, in the south, where Lu. longipalpis and CVL were found in 2010, HVL in 2018, and critical hotspots of CVL were found in the periphery. Moran's Global Index showed a weak but statistically significant spatial autocorrelation related to cases of CVL (I = 0.2572), and 11 municipalities were identified as priority areas for implementing surveillance and control actions. In RNHA11, a complex array of socioeconomic and environmental factors may be fueling the epidemic and sustaining endemic transmission of VL, adding to the study of a neglected disease in a region of São Paulo, Brazil.
**Additional Information:**	
**Question**	**Response**
**Financial Disclosure**<br><br>Enter a financial disclosure statement that describes the sources of funding for the work included in this submission. Review the submission guidelines for detailed requirements. View published research articles from *PLOS NTDs* for specific examples.<br><br>This statement is required for submission and **will appear in the published article** if the submission is accepted. Please make sure it is accurate.	ESF<br>FAPESP, Grant Number 2014/12494-0.

Powered by Editorial Manager® and ProduXion Manager® from Aries Systems Corporation

**Unfunded studies**<br><br>Enter: *The author(s) received no specific funding for this work.*<br><br>**Funded studies**<br><br>Enter a statement with the following details:<br>• Initials of the authors who received each award<br>• Grant numbers awarded to each author<br>• The full name of each funder<br>• URL of each funder website<br>• Did the sponsors or funders play any role in the study design, data collection and analysis, decision to publish, or preparation of the manuscript?<br>• **NO** - Include this sentence at the end of your statement: *The funders had no role in study design, data collection and analysis, decision to publish, or preparation of the manuscript.*<br>• **YES** - Specify the role(s) played.<br><br>* typeset	
**Competing Interests**<br><br>Use the instructions below to enter a competing interest statement for this submission. On behalf of all authors, disclose any competing interests that could be perceived to bias this work—acknowledging all financial support and any other relevant financial or non-financial competing interests.<br><br>This statement **will appear in the published article** if the submission is accepted. Please make sure it is accurate. View published research articles from *PLOS NTDs* for specific examples.	The authors have declared that no competing interests exist.

**NO authors have competing interests**<br><br>Enter: *The authors have declared that no competing interests exist.*<br><br>**Authors with competing interests**<br><br>Enter competing interest details beginning with this statement:<br><br>*I have read the journal's policy and the authors of this manuscript have the following competing interests: [insert competing interests here]*<br><br>* typeset<br><br>This statement is required for submission and **will appear in the published article** if the submission is accepted. Please make sure it is accurate and that any funding sources listed in your Funding Information later in the submission form are also declared in your Financial Disclosure statement.	
**Data Availability**<br><br>Authors are required to make all data underlying the findings described fully available, without restriction, and from the time of publication. PLOS allows rare exceptions to address legal and ethical concerns. See the PLOS Data Policy and FAQ for detailed information.	No - some restrictions will apply

A Data Availability Statement describing where the data can be found is required at submission. Your answers to this question constitute the Data Availability Statement and **will be published in the article**, if accepted.<br><br>**Important:** Stating 'data available on request from the author' is not sufficient. If your data are only available upon request, select 'No' for the first question and explain your exceptional situation in the text box.<br><br>Do the authors confirm that all data underlying the findings described in their manuscript are fully available without restriction?	
**Describe where the data may be found in full sentences. If you are copying our sample text, replace any instances of XXX with the appropriate details.**<br><br>• If the data are **held or will be held in a public repository**, include URLs, accession numbers or DOIs. If this information will only be available after acceptance, indicate this by ticking the box below. For example: *All XXX files are available from the XXX database (accession number(s) XXX, XXX.*).<br>• If the data are all contained **within the manuscript and/or Supporting Information files**, enter the following: *All relevant data are within the manuscript and its Supporting Information files.*<br>• If neither of these applies but you are able to provide **details of access elsewhere**, with or without limitations, please do so. For example:<br><br>*Data cannot be shared publicly because*	Some data cannot be shared publicly because they are not available on public sites but they can be obtained from the corresponding author under request.

of [XXX]. Data are available from the XXX Institutional Data Access / Ethics Committee (contact via XXX) for researchers who meet the criteria for access to confidential data.<br><br>*The data underlying the results presented in the study are available from (include the name of the third party and contact information or URL).*<br>• This text is appropriate if the data are owned by a third party and authors do not have permission to share the data.<br><br>* typeset	
Additional data availability information:	Tick here if the URLs/accession numbers/DOIs will be available only after acceptance of the manuscript for publication so that we can ensure their inclusion before publication.

Powered by Editorial Manager® and ProduXion Manager® from Aries Systems Corporation

Manuscript
Click here to access/download;Manuscript;MANUSCRIPT FILE CLEAN VERSION 02_21_2021.docx

Cases and distribution of visceral leishmaniasis in western São Paulo: a neglected disease in this region of Brazil

Regiane Soares Santana[1], Karina Briguenti Souza[1], Fernanda Lussari[1], Elivelton Silva Fonseca[1], Cristiane Oliveira Andrade[2], Marcia Kaihara Meidas[3], Lourdes Aparecida Zampieri D'Andrea[4], Francisco Assis Silva[1], Edilson Ferreira Flores[5], Ivete Rocha Anjolete[6], Luiz Euribel Prestes-Carneiro[1]*

[1]Department of Pós-Graduation, Environment and Regional Development Program, Oeste Paulista University, Presidente Prudente, São Paulo, Brazil

[2]Department of Control of Vectors, Teodoro Sampaio Municipality, Teodoro Sampaio, São Paulo, Brazil

[3]Bioclinic Clinical Laboratory, Teodoro Sampaio, São Paulo, Brazil

[4]Center for Biomedical Sciences and Regional Laboratory, Adolfo Lutz Institute, Presidente Prudente, São Paulo, Brazil

[5]Statistics Department, School of Sciences and Technology, São Paulo State University, Presidente Prudente, São Paulo, Brazil

[6]Supervision and Control of Endemics, Presidente Prudente, São Paulo, Brazil

*Corresponding author

luiz@unoeste.br (LEP-C)

The manuscript was edited by a native speaker: authorserv@gmail.com; Invoice no: 200103

Abstract

Visceral leishmaniasis (VL) is one of the most prevalent parasitic diseases worldwide. In 2019, 97% of the total numbers of cases in Latin America were reported in Brazil. In São Paulo state, currently 17.6% of infected individuals live in the western region. To study this neglected disease on a regional scale, we describe the spread of VL in 45 municipalities of the Regional Network for Health Assistance11(RNHA11). Environmental, human VL (HVL), and canine VL (CVL) cases, Human Development Index, and *Lutzomyia longipalpis* databases were obtained from public agencies. Global Moran's I index and local indicators of spatial association (LISA) statistics were used to identify spatial autocorrelation and to generate maps for the identification of VL clusters. On a local scale, we determined the spread of VL in the city of Teodoro Sampaio, part of the Pontal of Paranapanema. In Teodoro Sampaio, monthly peri-domicile sand fly collection; ELISA, IFAT and Rapid Test serological CVL; and ELISA human HVL serum surveys were carried out. In RNHA11 from 2000 to 2018, *Lu. longipalpis* was found in 77.8%, CVL in 69%, and HVL in 42.2% of the 45 municipalities, and 537 individuals were notified with HVL. Dispersion occurred from the epicenter in the north to Teodoro Sampaio, in the south, where *Lu. longipalpis* and CVL were found in 2010, HVL in 2018, and critical hotspots of CVL were found in the periphery. Moran's Global Index showed a weak but statistically significant spatial autocorrelation related to cases of CVL (I = 0.2572), and 11 municipalities were identified as priority areas for implementing surveillance and control actions. In RNHA11, a complex array of socioeconomic and environmental factors may be fueling the epidemic and sustaining endemic transmission of VL, adding to the study of a neglected disease in a region of São Paulo, Brazil.

Author summary

Visceral leishmaniasis (VL) is considered the second most important disease caused by a protozoan worldwide. Currently, 97% of the cases in Latin America are in Brazil. In São Paulo state, since 1997, the disease has been found in an increasing number of municipalities, mainly in the western region. The reasons why VL is spreading in a crescent shape in this region is not well understood, however, socioeconomic and environmental risk factors that increase vulnerability to the disease may be involved. From 2000 to 2018, vectors of *Lutzomyia longipalpis* were found in 77.8%, canine VL in 69%, and human VL in 44.4% of the 45 municipalities of the western region. Dispersion occurred from the epicenter in the north and followed central and radial highways, reaching Teodoro Sampaio in the south. Among the 45 municipalities, 11 were identified as priority areas for implementing surveillance and control actions. Considered the last frontier and one of the poorest regions of São Paulo, our findings are relevant for planning interventions aimed at reducing the cases of VL in the region.

Introduction

Visceral leishmaniasis (VL) is one of the most prevalent parasitic diseases worldwide. In 2017, 94% of new cases occurred in seven countries: Brazil, Ethiopia, India, Kenya, Somalia, South Sudan, and Sudan [1]. In 2019, 97% of the total numbers of cases in Latin America were reported by Brazil [2]. Currently, 25 of 27 Brazilian states notified autochthonous cases of canine visceral leishmaniasis (CVL) and 23 notified cases of human visceral leishmaniasis (HVL) [3].

In São Paulo, the richest and most populous state, *Lutzomyia longipalpis* sand flies were found in 1997, infected dogs in 1998, and infected humans in 1999. In 2020, 106 of 645 (16.4%) municipalities registered cases of HVL [4].

In recent years, from where and how VL reached the western region of São Paulo has been raised. Epidemiological studies demonstrated that the disease crossed the Bolivian border with Brazil and was found in the cities of Corumbá and Ladário, on the western side of the state of Mato Grosso do Sul (MS), expanded to its capital, Campo Grande, crossed the entire state and reached Três Lagoas, located on the western border of São Paulo. From MS to SP, the first diffusion occurred through the cities along with the construction of the Novoeste railway (1909–1952), the (BR-300) highway, completed in 1980, and the construction of a pipeline (1998) [5]. In São Paulo, CVL and HVL were first found in Araçatuba, a city harboring the railway, the highway, and the pipeline [6]. However, following the Candido Rondon highway (BR300), the disease crossed the state from the western region to the east toward the capital [6]. In the 45 municipalities of RNHA11 mesoregion, in the western region of São Paulo, in the border of MS, the first phlebotomine systematic collection was found in Dracena, a city located in the north and considered the epicenter [7]. In 2020, the western region harbored 537 of 3042 (17.6%) individuals infected with VL in São Paulo [4].

It has been hypothesized that in the western region, the presence of socioeconomic and environmental risk factors may favor the biological cycle of the disease, increasing the presence of *Leishmania (Leishmania) infantum chagasi* parasites, *Lu. longipalpis* vectors, and infected *Canis familiaris*, which in turn infect humans. Also, no study has been conducted on the dispersion of VL in the western region of São Paulo using an integrative approach so far. Our study is innovative because it allows the construction of a framework on two geographical scales: the regional scale combines public databases of the 45 municipalities of RNHA11 mesoregion; the local scale combines databases of Teodoro Sampaio, a representative

city of RNHA11. To study the spread of this neglected disease in the 45 municipalities of RNHA11 on the regional scale, public databases on vectors, dogs, humans, environment, and socioeconomic risk factors were accessed. On a local scale, an entomological, canine, and human VL survey was conducted in Teodoro Sampaio, considered the capital of Pontal of Paranapanema.

Methods

Study design and setting

Regional characteristics

In 2020, São Paulo, the richest and most populous state of Brazil, with an estimated population of 44,639,899, accounts for 21.0% of the entire population of Brazil, estimated to be 213,930,425 inhabitants according to Brazilian Census (IBGE) (Fig 1). The state comprises 645 municipalities, bordering Minas Gerais (north and northeast), Rio de Janeiro (northeast), Paraná (south), Mato Grosso do Sul (MS) (west), and the Atlantic Ocean (east) (Fig 1A). It is divided geographically into 15 mesoregions and 18 Regional Networks for Health Assistance (RNHAs). The western region comprises 45 municipalities and an estimated population of 753,344 inhabitants (2018) and it is administered by RNHA11, located in Presidente Prudente, mesoregion 8. The region is characterized by municipalities with a population <20,000 [8]. Among the 45 municipalities that are part of RNHA11, a group of 23 municipalities is called Pontal of Paranapema region, because of its relationship with the Paranapanema river. The Pontal of Paranapanema covers an area of 18,392.16 km^2 and has an estimated population of

620,953 (IBGE 2018) (Fig 1A). In contrast to the dynamic economic situation in the rest of the state, it is considered one of the poorest regions of São Paulo state [9].

Local characteristics

Teodoro Sampaio (latitude 22º31′57″ S and longitude 52º10′03″ W) at an altitude of 321 meters above sea level has an estimated population of 23,019 (IBGE 2018). It is a small-sized urban town located 651 km from the state capital, and in the middle of a State Park and a high concentration of rural settlements. The municipality covers an area of 1,556 km^2, currently the 8th largest municipality in São Paulo (IBGE) (Fig 1A; Fig 1Ba). The municipality has sandy soil and a typical tropical climate with dry winters and wet summers, with an average annual temperature of 26°C varying from 16°C to 33°C.

The canine population of Teodoro Sampaio was estimated to be 5,754 animals, with a ratio of 1 dog per 4 inhabitants as previously determined for the region [10]. Devil's Hill State Park (Parque Estadual Morro do Diabo) is located 6.3 km from the urban area of Teodoro Sampaio. Covering an area of 338.5 km^2, it is one of the largest stretches of Tropical Forest in São Paulo state (Fig 1Bb). In the 1,950s, the whole region of RNHA11 was covered by a Tropical Forest that was almost completely destroyed. The Tropical Forest in Pontal of Paranapanema was extensively destroyed, especially in the middle of the 20th century, replaced by agricultural activities and urbanization. More than 3,000 km^2 of dense vegetation was reduced to 370 km^2 in less than 20 years (12.3%), including the Devil's Hill State Park and other small fragments. Flowing along the border of the city, the Paranapanema River is one of the biggest and most important rivers in the southeastern region of Brazil, and is a natural border between São Paulo and Paraná states (Fig 1Bc). It is 930 km long and since the 1,980s, it includes 11 large artificial lakes and an extensive flooded area, supporting hydroelectric plants and

bridges. These bridges link dozens of small towns and villages connected by a large number of highways (Fig 1A).

Potential dispersion routes of VL infection in RNHA11 mesoregion

RNHA11 is connected by two important radial highways: (1) the Marechal Candido Rondon (SP 300) linking two VL endemic regions: Três Lagoas, on the border of MS and Araçatuba-Bauru in the center-northeastern region of São Paulo; (2) the Euclides Figueiredo highway (SP 563) linking the VL endemic regions of Pereira Barreto, Ilha Solteira, and Andradina to Dracena, one of the most important cities of RNHA11, also with the highest prevalences in VL. Secondary highways also link the RNHA11 from north to south in direction to Paraná state, a region free of VL so far. Teodoro Sampaio is connected by a network of radial and secondary highways. One of the most important is the secondary vicinal highway (VSP-031; VSP-035) connecting the endemic regions of Presidente Epitácio and Panorama, bordering the Paraná River to Teodoro Sampaio (Fig1A)

Fig 1. Road network, rivers and artificial lakes, and location of Teodoro Sampaio in RNHA11 of São Paulo state. (A) The highway network between the municipalities of RNHA11 and Teodoro Sampaio. Artificial lakes and bridges on the Paraná River linking RNHA11 municipalities to well-known endemic regions in Mato Grosso do Sul state, and bridges over artificial lakes in the Paranapanema river linking regions with VL to VL-free areas in Paraná state. (B) A Google Earth image of the municipality of Teodoro Sampaio: (a) the urban area of Teodoro Sampaio, (b) the Devil's Hill State Park, 6.3 km from the urban area, (c) the artificial lake and islands of the Paranapanema River, 0.9 km from the urban area.

Data on *Lu. longipalpis*, canine visceral leishmaniasis (CVL) and HVL in the 45 municipalities of RHN11were obtained from archival databases from Brazilian public health agencies. *Lu longipalpis*: Supervision in Control of Epidemic (SUCEN),

http://www.saude.sp.gov.br/sucen-superintendencia-de-controle-de-endemias/homepage/downloads/arquivos-leishmaniose-visceral; Canine visceral leishmaniasis: Center of Regional Laboratory of the Adolfo Lutz Institute of Presidente Prudente V (CRL-ALI-PPV), http://www.ial.sp.gov.br/ial/o-ial/quem-e-quem/centro-de-laboratorio-regionais, under formal request; Human visceral leishmaniasis: Epidemiological Surveillance Center (CVE-SP), http://www.saude.sp.gov.br/cve-centro-de-vigilancia-epidemiologica-prof.-alexandre-vranjac/areas-de-vigilancia/doencas-de-transmissao-por-vetores-e-zoonoses/.

Data on the presence of Lu. longipalpis in the municipalities of RNHA11

In RNHA11, data on phlebotomine sand flies identified in entomological collections were obtained from SUCEN in the period from 2000 to 2019. Entomologic surveys are proposed by the Manual of Surveillance and Control of Visceral Leishmaniasis of São Paulo (VLCPSP) [11], aiming to monitor the distribution of *Lu. longipalpis* in silent non-receptive, non-vulnerable municipalities, and silent non-receptive vulnerable municipalities as defined by the absence of confirmed autochthonous cases of HVL and CVL, without the presence of the chosen vector or not by estimated values of the distance to characterize the vulnerability. In these municipalities, an entomologic survey is recommended for the detection of the presence of *Lu. longipalpis* and to provide information on its distribution, allowing identification of the risk areas where control measures should be intensified [11].

Canine and human visceral leishmaniasis and Center of Zoonosis Control in the municipalities of RNHA11mesoregion

According to VLCPSP guidelines [11], in São Paulo state, the municipalities are divided into two groups: silenced for VL, that is, without confirmation of autochthonous

human and/or canine cases; and with transmission, that is, with confirmation of autochthonous human and/or canine cases. The investigation of a case of CVL may occur by a passive or house-to-house survey, conducted by the Center of Zoonosis Control (CZC) or by the Municipal Zoonosis Service (MZS) of each municipality. An active house-to-house search is conducted when *Lu. Longipalpis* is found in a particular area of the city or every time a dog is diagnosed with CVL; sequentially, all households within a radius of 200 m are visited and the dogs are tested for CVL. A passive survey is conducted when a dog’s owner finds the symptoms described by the CZC or MZS as suspicious for CVL, as previously published [9]. Data on CVL, presence, and characteristics in each municipality under the CZC in the RNHA11 mesoregion were obtained from Adolfo Lutz Institute of Presidente Prudente [12]. A suspected case of HVL is confirmed if it meets one of the following criteria: laboratory criteria, that is, identification of *Leishmania (Leishmania) infantum chagasi*, from a culture and/or inoculation in hamsters and/or by molecular techniques; presence of *Leishmania* spp. in the direct parasitological examination; presence of anti- *Leishmania (Leishmania) infantum chagasi* antibodies in serological tests and clinical-epidemiological criteria, that is, a patient from the area of transmission of VL, with suggestive clinical signs and without laboratory confirmation, provided that other diagnostic hypotheses are ruled out, that presents a favorable response to therapeutic treatment. Data on patients infected with HVL in RNHA11 were obtained from the Epidemiological Surveillance Center of São Paulo, which organizes and publishes data on recorded infectious diseases in São Paulo state annually [4].

Mapping the cluster of identification of canine visceral leishmaniasis in RNHA11 mesoregion

In RNHA11 mesoregion, a Moran map was utilized to assess the formation of clusters of municipalities associated with the presence of CVL. Global spatial autocorrelation indicators, such as the Moran Index, present a single value as a measure of the spatial association for the data, which are presented by area, providing an index for the entire study region. However, it is often desirable to examine patterns on a more detailed scale to see if the process stationary hypothesis is verified locally, that is, if there is spatial dependence. Therefore, it is necessary to use spatial association indicators that can be associated with the different locations of a spatially distributed variable. This methodology uses the Local Moran Index (LISA) to find the spatial correlation of these areas. As it is a local indicator, there is a specific correlation value for each area, thus allowing the identification of clusters of areas and outliers [13,14]. Values vary from −1 to 1; values approaching zero indicate the absence of significant spatial autocorrelation of the values with their neighbors; values below 0.50 indicate a weak autocorrelation; values between 0.50 and 0.75 indicate medium autocorrelation; and values above 0.75 indicate strong autocorrelation. Positive values indicate a positive autocorrelation, where the value of what is being evaluated is similar to the values of its neighbors, and negative values indicate a negative autocorrelation [13-15].

Mapping the Human Development Index in the RNHA11 mesoregion

The Human Development Index (HDI) was used as an indicator for understanding the socioeconomic profile of the municipalities of São Paulo state. The HDI is a well-known and comparable indicator worldwide, reproducing studies in other states and regions countrywide [16]. The HDI value of the 45 municipalities in the RHN11 was

obtained in Fundação Sistema Estadual de Análises de Dados (SEADE), from 1999 to 2016, coded and classified by the denominated quintile as a reference parameter. Thematic maps were created for the HDI using ArcGis 10.7.1 software. The HDI was analyzed with support in a predictive method of interpolation surface generation: local polynomial interpolation (LPI). The Gaussian kernel method was used to generate the surface, classified according to the quintile. The gain in the LPI analysis in relation to the global polynomial interpolation presupposes it overlaps in several concentrations that a spatial representation may have, in our study, through a point in the centroid city of São Paulo state.

Entomological survey in Teodoro Sampaio

In the urban area of Teodoro Sampaio, the phlebotomine sand fly entomological survey began in 2000, monthly, from February to October during the most favourable periods for detecting the presence of the vector. The survey ended in October 2010 when *Lu. longipalpis* was found in all areas. To standardize collections, the number (ranging from 1 to 40) and frequency (ranging from 1 to 39) of CDC light traps (Horst Ltd, São Paulo, Brazil) installed varied according to the density and infestation of sand flies. The traps were placed approximately 1 m from the ground in places susceptible to the presence of phlebotomies and/or in the shelters of domestic animals, with an average temperature above 20°C and relative humidity >70%. In each sector of the different areas, four areas with risk factors for the presence of phlebotomies were selected, including a large peri-domicile, and areas with abundant vegetation, accumulation of organic matter in the soil, and the presence of domestic animals from which the female sand fly can potentially obtain an infected blood meal. The traps were operated between 17:00 and 07:00 h. Monthly, four traps were installed for three nights in each sector. After capture,

nylon-mesh cages were placed in plastic bags, tagged, and refrigerated (1°C–7°C) until identification was performed by the Regional Entomology Laboratories of SUCEN, Coordination of Disease Control, Secretary of State of Health, located in Presidente Prudente, São Paulo state. The sand flies were processed and identified using the taxonomic key of Galati [17].

Canine visceral leishmaniasis survey in Teodoro Sampaio

The canine survey took place in four sectors of the urban area of Teodoro Sampaio, from January 2010 to December 2018, by a passive or house-to-house survey conducted by the Center of Zoonosis Control (CZC). We used recorded data of 3749 domiciled dogs of different ages, submitted to serum survey, using antibody tests to detect *Leishmania*, according to VLCPSP guidelines [11]. In Teodoro Sampaio, the Center of Regional Laboratory of the Adolfo Lutz Institute of Presidente Prudente (CRL-ALI-PPV) is responsible for the diagnostic tests for CVL. The VLCSP supervises the main actions to reduce morbidity and mortality, aimed at early diagnosis and treatment of human cases, vector control, identification, and euthanasia of seropositive domestic dogs. In 2009, the Ministry of Health began using enzyme-linked immunosorbent assays (ELISAs), produced by Bio-Manguinhos/Fiocruz, Ministry of Health, Rio de Janeiro, Brazil, for screening and confirmatory tests in CVL serological surveys. For ELISA-positive samples, a confirmatory test using IFAT, produced by Bio-Manguinhos/Fiocruz, was performed. From April 2012 to the present, the Brazilian Ministry of Health replaced the ELISA/IFAT tests with a new protocol using a dual-path platform CVL rapid test, produced by Bio-Manguinhos/Fiocruz, with ELISA-CVL as a confirmatory test. Animals with positive samples in the screening test (ELISA from January 2009 to March 2012 and the dual-path platform CVL rapid test from April 2012 until the present and a negative result in the confirmatory test (IFAT and ELISA,

respectively) were re-sampled. Only concordant samples that were positive in the screening test and positive in the confirmatory test were considered seropositive [9]. Data on the presence and characteristics in each municipality under the CZC in the RNHA11 mesoregion were obtained from Adolfo Lutz Institute of Presidente Prudente [12].

Human visceral leishmaniasis in Teodoro Sampaio

Data on human leishmaniasis was obtained from the Epidemiological Surveillance Municipality of Teodoro Sampaio. The sole case of HVL in Teodoro Sampaio was referred to the Regional Hospital of Presidente Prudente, a tertiary, university, public, and reference center for treatment of HVL in RNHA11 mesoregion. Confidentiality was assured. The case was confirmed in 2018 by clinical/epidemiologic criteria and laboratory diagnostics according to the VLCPSP guidelines [11].

Human visceral leishmaniasis serological survey and laboratory diagnosis in Teodoro Sampaio

In July 2018, a serological survey was conducted on 159 individuals aged from 18 to 65 years from four different locations: a health care center, a Family Health Strategy Unit; the Social Assistance Reference Center; a State Technical School; and in individuals attending a private clinical laboratory. Blood was collected blood collections tubes under vacuum with and without EDTA as an anticoagulant. Serum was separated by centrifugation and stored at −200°C until use. The analysis was performed at the CRL-ALI-PPV using a qualitative test for the detection of antibodies against HVL by ELISA, according to the manufacturer’s instructions (LEISHMANIA ELISA IgG+IgM; VIRCELL, Santa Fe, Granada, Spain). The test uses recombinant antigens capable of fixing specific antibodies to *Leishmania (Leishmania) infantum chagasi* with a

sensitivity of 97% and specificity of 99%. Positive tests were confirmed by indirect immunofluorescence (Bio-Manguinhos/Fiocruz, Ministry of Health, Rio de Janeiro, Brazil). Hemograms and hepatic enzymes were determined because hepatic function may be affected and pancytopenia may be present in patients infected with VL. Hemograms were performed using a flow cytometer flux counter (Pentra 80, HoribaDiagnostics, Montpellier, France) and the differential leukocyte count was compared with direct microscopic observation of blood smears. Alanine aminotransferase (ALT), aspartate aminotransferase (AST), and albumin were assessed using automated systems according to the manufacturer’s instructions; the normal ranges were 7–55 U/L, 8–43 U/L, and 3.5–5 g/dL, respectively. A total of 16,636 individuals were considered to determine the sample size. These samples were processed by Laboratório Bioclínico, Teodoro Sampaio, SP, Brazil.

Kernel maps construction

Dogs that underwent the serological survey and those with positive serology were registered according to the location used by the municipal health surveillance service. The city was divided into four sectors, starting from the northeast of the urban area in a clockwise direction. These sectors, in turn, were divided into 432 blocks, arranged in a polygon shape, used to generate the Geographic Information System and served as a basis for indexing the attributes of the surveyed blocks. With the geometric points generated per block, it was possible to visualize the number of dogs with CVL in each block, the number of dogs in the census for each block, and the number of euthanized dogs per block. The number of positive dogs per block is not consistent with the number of euthanized dogs, because there were some cases in which euthanasia of dogs that tested positive was not performed. For the procedure that identified hot areas of CVL infection, we used the Kernel density maps, using the euthanized dogs as a population

field to generate a continuous surface to the urban area of the Teodoro Sampaio. A raster surface was generated using the Gaussian equation, with a search ratio equivalent to a block (100 m) and a pixel size equivalent to a household (10 m). The coordinate system used as reference was WGS1984. Regarding the parameters for reading the data for analysis, the urban area of Teodoro Sampaio measures 4 × 3 km^2.

Ethical statement

The project was approved by the ethics committee of Unoeste (number 4030). Canine samples were collected with the written informed consent of owners. Ethical approval was obtained from the Adolfo Lutz/Pasteur Institute Ethics Committee on the Use of Animals (number 02/2016). The study is for scientific research purposes and is in compliance with Federal Law no. 11794 of October 8, 2008, Decree no. 6899 of July 15, 2009, and in accordance with regulations outlined by The National Council for Animal Experimentation Control (Conselho Nacional para Controle da Experimentação Animal, CONCEA).

Statistical analysis

The results are shown as means standard error of the mean (SEM) (for normally distributed variables). Dichotomous and nominal variables are expressed as frequencies and percentages. Pearson's correlation coefficient was used to describe the functional relationship between the number of cases of HVL per 10,000 inhabitants and the HDI of each municipality. Statistical analysis was performed using GraphPad Software (San Diego, CA, USA) and the Sigma-Stat program (Systat Software, Richmond, CA, USA). ArcGIS 10.7 and ArcGis Pro were used for data analysis and layout design.

Results

The presence of Lu. longipalpis in the municipalities of RNHA11 mesoregion

In São Paulo, *Lu. longipalpis* sand flies were found for the first time in Araçatuba, an area surrounding the western region in January 1997. From 1997 to July 2019, the vector was found in 203 of 645 (31.5%) of the municipalities. In RNHA11 mesoregion, *Lu. longipalpis* was detected for the first time in Dracena in 2003 in the north; the sand flies dispersed toward the south in the direction of the municipalities of Pontal of Paranapanema and Paraná state. In July 2019, *Lu. longipalpis* was found in 35 of 45 (77.8%) of the municipalities of RNHA11 mesoregion.

Canine visceral leishmaniasis cases and the presence of the Center of Zoonosis Control in municipalities of RNHA11mesoregion

In RNHA11, CVL was described for the first time in Dracena in 2005. In May 2018, CVL transmission was present in 29 of 45 (64.4%) of municipalities of RNHA11. Among the municipalities, 13 of 45 (28.9%) do not have CZC, 4 of 45 (8.9%) have a partial physical and human structure of CZC, and 28 of 45 (62.2%) have a structured CZC (Fig 2).

Fig 2. Spatial distribution of the HDI, the presence of CZCs, and populations >40,000 inhabitants in municipalities of RNHA11 mesoregion. Source: State System Data Analysis Foundation (SEADE, 2017). Base map: digital meshes of IBGE (2010). The municipalities are numbered as follows: 1, Paulicéia; 2, São João do Pau D'alho; 3, Monte Castelo; 4, Santa Mercedes; 5, Nova Guataporanga; 6, Junqueirópolis; 7, Irapuru; 8, Tupi Paulista; 9, Panorama; 10, Ouro Verde; 11, Dracena; 12, Presidente Epitácio; 13, Caiuá; 14, Presidente Venceslau; 15, Piquerobi; 16, Ribeirão dos Índios; 17, Emilianópolis;18, Flora Rica;19, Santo Expedito; 20, Alfredo

Marcondes; 21, Marabá Paulista; 22, Santo Anastácio; 23, Presidente Bernardes; 24, Alvares Machado; 25, Presidente Prudente; 26, Caiabu; 27, Martinópolis; 28, Rancharia; 29, João Ramalho; 30, Quatá; 31, Rosana; 32, Euclides da Cunha Paulista; 33, Teodoro Sampaio; 34, Mirante do Paranapanema; 35, Sandovalina; 36, Tarabai; 37, Pirapozinho; 38, Estrela do Norte; 39, Narandiba; 40, Anhumas; 41, Regente Feijó; 42, Indiana; 43, Taciba; 44, Nantes; 45, Iepê.

Spatial cluster analysis of CVL cases in RNHA11 mesoregion

The cases of CVL in the 45 municipalities of RNHA11 mesoregion during the period from 1999 to 2019 was totalled by the municipality. The Global Moran Index was applied and presented a weak spatial autocorrelation, but statistically significant (I = 0.2572). Applying the Local Moran Index Cluster Map (LISA) revealed that most municipalities (34 of 45; 75.5%) were non-significant, however, 11 of 45 (24.5%) showed significant spatial autocorrelation, distributed in the following quadrants: high-high with 5 municipalities Q (+/+), Presidente Venceslau, Ouro Verde, Dracena, Tupi Paulista, and Junqueirópolis; low-low with 3 municipalities Q (−/−), Martinópolis, Taciba, and Presidente Bernardes; low-high with 2 municipalities Q (−/+), Piquerobi and Ribeirão dos Índios; and in the high-low quadrant, only one municipality Q (+/−), Presidente Prudente. In the LISA map, we can see the p value of significant municipalities (Fig3).

Fig 3. Spatial distribution of high-risk clusters for CVL transmission, 2005-2018. Among the 45 municipalities of RNHA11, 11 are classified as high-risk clusters. They are concentrated mainly in the north region, considered the epicenter of the disease. (A) Univariate LISA and (B) LISA significance.

Human visceral leishmaniasis in RNHA11 mesoregion

Figure 4A shows that over 14 years of follow-up (2005–2018), 19 of 45 (42.2%) municipalities in RNHA11 mesoregion referred patients infected with VL. In the

northern area, all the municipalities' notified cases, most of them at increased rates (17-157 cases), and Dracena (dark red), considered to be the epicenter, showed the highest rates (58–176 cases). On Pontal of Paranapanema, 8 of 32 (25.0%) municipalities registered HVL, and dispersion on the regional scale occurred in three different ways: irradiation, contiguity, and jumps (when municipalities far from the endemic region register cases of CVL or HVL). In municipalities located along the Paraná River, only one did not register cases (Fig 4A). Figure 4B shows that cases were found first in Dracena in 2005, in subsequent years HVL spread to the neighboring municipalities and in 2010 reached the municipalities of Pontal of Paranapanema. Teodoro Sampaio, 133 km from the epicenter, was one of the last municipalities to register cases and HVL was detected in 2018.

Fig 4. Geographic distribution of human visceral leishmaniasis in the RNHA11 mesoregion between 2005 and 2018. The colors represent the cumulative number of cases (A). Flow and spatial dispersion of human visceral leishmaniasis from 2005 to 2018 in the RNHA11 mesoregion, and the year in which the first case was detected in each municipality (B).

Paraná and Paranapanema Rivers, dams and artificial lakes in RNHA11 mesoregion and surrounding areas of Teodoro Sampaio

Figure 1A and Figure 4A show that the municipalities in the RNHA11 mesoregion are divided from MS on the western side by the Paraná River, which flows along the border of five cities. On the south side, the region is divided from Paraná state by the Paranapanema River, which flows along the border of ten municipalities. Together, the rivers support seven large lakes and a flooded area of 6175 km^2 and seven dams and bridges (Fig 1A). These bridges link the VL endemic regions of Três Lagoas in MS and Andradina in São Paulo state to the areas of the western region as well as the municipalities of Pontal of Paranapanema to the VL-free areas of Paraná state. The

urban area of Teodoro Sampaio (Fig 1Ba) is surrounded by the Devil's Hill State Park (Fig 1Bb), 6.3 km away, and linked by a highway with the administrative offices, museums, laboratories, and environmental educational areas of the park. The municipality of Teodoro Sampaio is served by both the Paraná and Paranapanema Rivers, which surround the urban area of the city at a distance of 1 km (Fig 1 Bc). In the region, the average annual precipitation varies between 1200 and 1500 mm, and the average annual temperature remains slightly above 22°C.

The Human Development Index in the RNHA11mesoregion

In the municipalities in this study, the HDI varied from very low in 4 municipalities and low in 15 municipalities to high only in Presidente Prudente, the most important city in RNHA11 mesoregion. Of the areas with very low and low HDIs, 18 (40%) of the municipalities are located in the Pontal of Paranapanema region. Ribeirão Preto, connecting the Paraíba Valley to the Metropolitan region of São Paulo city, and from Campinas to Piracicaba presents an axis of great development, with the HDI varying from 0.790 to 0.826. The regions with the lowest HDI values are the Ribeira Valley, located in the south of the state, and Pontal of Paranapanema (our study area). In Teodoro Sampaio, rates vary from 0.678 to 0.715. In São Paulo state, the HDI is distributed by regions, and maps are available in Fundação Sistema Estadual de Análise de dados (SEADE), Teodoro Sampaio is surrounded by very poor municipalities, Euclides da Cunha Paulista, Marabá Paulista, and Caiuá, with rates between 0.6770 and 0.704 (Fig 2).

When the functional relationship between the number of cases of HVL per 10,000 inhabitants and the HDI of each municipality was calculated, the correlation (adjusted R value) had a value less than 10% and the *P* value was not significant, however, when

municipalities that did not have cases of VL were removed and the simple linear regression technique was applied, the regression line showed a trend for lower values of VL cases per 10,000 inhabitants as the HDI increased (Fig 5).

Fig 5. Correlation between HDI and VL infection in municipalities in RNHA11 mesoregion.

Entomological survey in the urban area of Teodoro Sampaio

Teodoro Sampaio covers two areas with regard to operational disease control programs (e.g. dengue fever surveillance and control, CVL and HVL surveillance and control). The two areas are divided into four sectors and the sectors are divided into blocks (Fig 6A and B). *Lu. longipalpis* were initially found in July 2010 in area 1, sectors 2 and 4 in blocks 146 and 298, and in October 2010 in area 1, sector 1, and block 75 (Table 1).

Table 1. Spatiotemporal distribution of *Lu. longipalpis* sand flies in the urban area of Teodoro Sampaio, São Paulo, Brazil.

**MUNICIPALITY OF TEODORO SAMPAIO**						
**AREA**	**SECTOR**	**BLOCK**	**DATE**	**No. OF TRAPS**	**No. OF TIMES**	**SPECIES/NUMBER**
1	2	146	22 July 2010	9	9	*Lu. longipalpis* 1 male
1	4	298	27 July 2010	10	10	*Lu. longipalpis* 1 male
1	1	75	25 October 2010	14	14	*Lu. longipalpis* 1 male
		Total		33	33	3 males

Canine serological survey and spatial distribution of euthanized dogs in Teodoro Sampaio

In 2010, CVL was identified for the first time in the urban area of Teodoro Sampaio. From January 2010 to December 2018, 3749 dogs were selected for serology by the CZC and 298 animals (7.9%) were positive for CVL. Fig 6A shows the kernel

spatiotemporal distribution of euthanized dogs. Of the 298 dogs that were positive, 110 (36.9%) were euthanized according to the criteria of the Brazilian Ministry of Health. In 2010 and 2011, infected dogs were identified and euthanized in sectors 1, 2, and 3 (three hotspots; dark red color). In 2012–2013, infected dogs were found in the areas surrounding the sectors where dogs positive for CVL were found in 2010–2011 (ochre color). From 2014 to 2017, positive cases reached the entire urban area, including sector 4 (apple green and green colors), and in 2018 no euthanized dogs were registered. Fig 6B shows the hotspots of infected dogs euthanized. The periphery of sector 1 showed a higher number, demonstrated by critical points hotspots, whereas sector 4 showed the lowest number. These data allow us to consider that a stronger force of infection occurs in sector 1, the main hotspot. The first case of HVL in the city was located in this sector. Several dogs were not euthanized for different reasons.

Fig 6. Kernel of spatiotemporal distribution and density estimation of CVL seropositive euthanized dogs in Teodoro Sampaio, 2010–2018. Kernel of the spatiotemporal distribution of CVL diagnosed and euthanized dogs (A). The hotspots represent areas where a higher density of seropositive dogs was observed, from 2010 to 2018 (B). In the map legend, the lighter colors represent the low density of CVL cases and the darker colors represent the presence of hotspots.

Cases of human visceral leishmaniasis in Teodoro Sampaio

Teodoro Sampaio, 133 km from the epicenter, was one of the last municipalities to register cases and HVL was detected in 2018. The first autochthonous case of HVL was identified in 2018 in the eastern area, area 1, on the outskirts of Teodoro Sampaio (Fig 6B), where a high number of cases of CVL were found throughout the period. The patient was a 48-year-old man who did not respond to treatment with meglumine antimoniate and was treated with liposomal amphotericin B (5 mg/kg/day) for 5 days with improvement in the symptoms and 50% reduction of splenomegaly.

Human visceral leishmaniasis serological survey, laboratory findings and epidemiologic characteristics of the study population in Teodoro Sampaio

Between January and December 2018, 159 individuals living in Teodoro Sampaio were enrolled in the human visceral serological survey, and 2 of 159 (1.3%) resulted positive; 4 of 159 (2.5%) resulted indeterminate and 153/159 (96.2%) resulted negative. Hematologic abnormalities are among the most common manifestations in patients infected with VL in whom pancytopenia may occur (Table 2). From 159 individuals analyzed, mild anemia occurred in 5 of 159 (3.1%) individuals, leukopenia occurred in 3 of 159 (1.9%), and thrombocytopenia occurred in 4 of 159 (2.5%). The mean values±SEM, with 95% confidence intervals (CI) were as follows: red blood cells, 4.81±0.03 million/ cells/mcL (95% CI, 4.50–4.62); hemoglobin (g/dL), 13.80±0.10 (95% CI, 13.59–14.00); hematocrit (%), 40.33±0.27 (95% CI, 39.78–40.88); mean corpuscular volume (fL), 88.49±0.33 (95% CI, 87.83–89.15); mean corpuscular hemoglobin (pg), 31.50±1.25 (95% CI, 29.02–33.99); mean corpuscular hemoglobin concentration (g/dL), 34.20±0.06 (95% CI, 34.07–34.34); platelets ($\times 10^3$/mcL), 266.359±4.740 (95% CI, 256.997–275.720). When the main changes in white blood cells were analyzed in the whole population, 17 of 159 (10.7%) showed mild leucopenia, 13 of 159 (8.1%) had mild leukocytosis, 25 of 159 (15.7.2%) had neutropenia, 41 of 159 (25.8%) had eosinophilia, 18 of 159 (11.3%) had lymphocytosis, and 11 of 159 (6.9%) had monocytosis. Mild thrombocytopenia was present in 9 of 159 (5.6%) individuals.

Table 2. Hematologic and biochemical features recorded in 159 individuals included in the human serological survey in Teodoro Sampaio, São Paulo state, Brazil, in July 2018

PARAMETER	NUMBER (%)	MEAN±SD	95% CI	NR
Erythrocytes (million/µL)				
Men <4.5	3 (6.6)	4.207±0.1677	3.790–4.623	5.00±0.5
Women <3.8	3 (2.6)	3.537±0.1050	3.276–3.798	4.3±0.5
Hemoglobin (g/dL)				
Men <13	1 (2.2)	12.50		15.0±2.0
Women <12	9	11.48±0.4410	11.14–11.82	13.5±1.5
Hematocrit (%)				
Men <40	4 (8.8)	38.58±0.8261	37.26–39.89	45±5
Women <39	57 (50)	36.90±1.632	36.47–37.33	41±5
MCV <83 (fL)	11 (6.9)	78.84±2.599	77.09–80.58	92±9
MCH <27 (pg)	6 (3.7)	25.70±0.8809	24.78–26.62	29.5±2.5
MCHC (g/dL) <31.5	0	0		33±1.5
RDW <11.6 (fL)	20	11.30±0.2038	11.20–11.39	12.8±1.2
Platelets<150 (mm^3)	4 (2.5)	133.3±7.274	121.7–144.8	150–400
Leukocytes <4.0 (mm^3)	3 (1.8)	133.3±7.274	2.515–4.471	7.0±3.0
Neutrophils <2.0 (mm^3)	0	0		2.0–7.0
Hepatic enzymes				
AST (IU)				
Men >50	2 (4.4)	85.50±40.31	0	10–50
Women >35	8 (7.0)	50.63±6.968	44.80–56.45	7–35
ALT (IU)				
Men>50	3 (6.6)	59.33±6.807	42.42–76.24	10–50 U/L
Women >35	12 (10.5)	56.50±20.87	43.24–69.76	10–35 U/L

ALT, alanine aminotransferase; AST, aspartate aminotransferase; MCH, mean corpuscular hemoglobin; MCHC, mean corpuscular hemoglobin concentration; MCV, mean corpuscular volume; NA, not available; RDW, red cell distribution width; NR, normal range .

In relation to the epidemiological characteristics of the study population, the mean age was 43.50±16.83 years (95% CI, 40.87–46.14; range, 18–89 years). The mean residence

time in the city was 23.86±15.27 years (95% CI, 21.46–26.25; range, 1–68 years). There were a greater number of women 115 of 158 (72.3%) than men because there are more women in the area where the research was carried. The age-related distribution was as follows: >18–20 years, 21(13.2%); 21–40 years, 52 (32.7%); 41–60 years, 55 (34.6%); and 61–80 years, 31 (19.5%).

Discussion

In 2005, HVL reached the municipalities in RNHA11 mesoregion, western São Paulo, and in 2010 the municipalities of Pontal of Paranapanema, a poor and asymmetric region. In Teodoro Sampaio, considered the capital of Pontal of Paranapanema, *Lu. longipalpis* and CVL were found in 2010 and HVL in 2018. In 2018, 19 of 45 (42.2%) municipalities in RNHA11 recorded cases. A complex array of socioeconomic and environmental factors may be fueling the epidemic and sustaining endemic transmission of VL (Fig 7).

~~**Fig 7. Socioeconomic structures and environmental changes related to the transmission of visceral leishmaniasis (VL) in the Regional Network for Health Assistance11 (RNHA11) mesoregion, São Paulo, Brazil.**~~ In the last decades, socioeconomic structures, and environmental changes have been modifying the landscape and the way of life of people in the region. Among the socioeconomic factors are: (1) the extensive number of roads linking small- and middle-sized cities from endemic to non-endemic areas; (2) low HDI due to deficiencies in sanitation, education, and low family income; (3) cities without a Center of Zoonosis Control or ineffective control of canine visceral leishmaniasis. Among the environmental changes are: (1) the presence of big Rivers and the construction of dams; (2) the formation of several artificial lakes in the Paraná and Paranapanema Rivers; extensive areas of flooding and increasing temperature and humidity in surrounding lakes; (3) extensive deforestation in the Tropical Forest and reduction of the forested area to 12%. Taken together, these factors have certainly

contributed to the increasing number of infected dogs, the proliferation of vectors, and the spread of human visceral leishmaniasis in the RNHA11 mesoregion of São Paulo.

The first *Lu. longipalpis* outbreak was found in the microregion of Dracena in 2003 [18]. One year later, vectors were found in Tupi Paulista and other adjacent municipalities, which suggested the existence of a possible route of dispersion by irradiation or by contiguity. However, *Lu. longipalpis* was registered in the region far from the epicenter, suggesting dispersion by jumps and toward the municipalities of the south, and HVL cases reached the municipalities of Pontal of Paranapanema in 2010 (Fig 4A and B). An extensive highway network (2382 km), linking endemic regions to small- and middle-sized cities that may function as potential routes of dispersion of vectors and infected dogs was demonstrated in RNHA11 mesoregion. We suggest that the main axis adding to VL dispersion is the Euclides Figueiredo highway (SP562). Starting in Ilha Solteira outside RNHA11, the highway goes down toward the cities of Andradina and Dracena and toward the municipalities of Pontal of Paranapanema, crossing Teodoro Sampaio at the border of Paraná state. Nevertheless, dissemination may also occur in lower rates by a secondary axis, SPV031 and SPV035, starting near Brasilândia, an endemic region in MS, and following the Paraná River through small-sized cities and ending in Teodoro Sampaio must also be considered. All the municipalities crossed by these highways became endemic areas of VL (Fig 4A). The potential role of highways in the spread of VL in the southeastern and southern states of Brazil has been pinpointed in recent years [5,6]. However, a new route has been described and has gained great importance. Connecting Foz do Iguaçu in the west, on the Brazilian side of the triple border and considered endemic for VL, to the port of Paranaguá in the extreme east, a large number of trucks carrying grain, fertilizers, fuel, and goods, circulate daily on BR 277, the most important highway in Paraná state. It

was suggested that following BR-277 and transects, coming from Argentina and Paraguay, seropositive dogs and *Lu. longipalpis* vector spread to the neighboring cities, becoming a region endemic to VL. Furthermore, potentially it is likely an important route of dispersion of infected dogs, vectors, and parasites to other regions of Paraná state [19,20].

In the western region, the proliferation of vectors could be aggravated by the extensive watershed flowing into the Paraná and Paranapanema Rivers, supporting hydroelectric dams, bridges and artificial lakes [21-25]. Porto Primavera dam in Paraná River has an artificial lake with a flooded area of 2.250 km^2 and divides the municipalities of RNHA11mesoregion and MS. Dividing São Paulo and Paraná state, the Paranapanema River flows on the border of ten municipalities with increasing rates of VL to the VL-free areas of Paraná state. Artificial lakes modify the climate of their surrounding areas as well as the environment within which the lake interacts [21-25]. In Presidente Epitacio, a city bordering the lake of Primavera dam, higher yearly temperatures rates of 23.93±3.06 °C (95% CI 22.54-25.32) were found compared with 22°C in municipalities far from the lakes [22]. The influence of artificial lakes of hydroelectric dams in Paranapanema River on the ecological aspects of the sand fly fauna in 3 different settings of an endemic area of CL in Paraná state was investigated. A wide dispersion of sand flies involved in the transmission of leishmaniasis was found in all the settings [21]. In Panorama, a city located on the border of the lake of the Paraná River, also an endemic area for VL, the high abundance of *Lu. longipalpis* had a significant correlation with temperature and humidity [26]. A relationship with climate and environmental factors with increased incidence rates of VL and annual precipitation, humidity, enhanced vegetation index, and night temperature values were found in the State of Tocantins, located in the northern region of Brazil [27].

In Teodoro Sampaio, although an entomological survey started in 2000, *Lu. longipalpis* sand flies were found only in 2010. It is well known that other phlebotomines, such as *Lu. migonei*, may be permissive vectors of VL in areas where *Lu. longipalpis* has not been recorded, but human or canine cases do occur [28-30]. According to the SUCEN database, in the period from 2000 to 2010, *Lu. cruzi* a VL vector found in Central-West of Brazil [31], and *Lu. migonei* were not identified in Teodoro Sampaio urban area. *Lu. migonei* was found in the wild forest of the Devil’s Hill in research carried out for an LT investigation in 1994, 1995, and 1996.

Although a direct association between HDI and the presence of VL in municipalities of RNHA11 mesoregion was not found, linear regression showed a tendency of a lower number of infected individuals in municipalities with higher HDI. It is well known that poverty induces health care inequalities, poor housing conditions, lower income, less education, or lower occupational skill levels, and the population tend to be less healthy than those who experience higher levels of social conditions in other areas [16,32,33]. Supporting the data, demographic, environmental, and behavioral characteristics of inhabitants of rural settlements, a vulnerable population, have been determined. In Teodoro Sampaio, only 7.1% had a high school education, 11.9% lacked elementary instruction, and 77.3% had completed primary school. The income varied from less than 100 US$ (91.6%) to about 170 US$ (8.4%) per month. Similar rates were found in a neighboring rural settlement in Mirante of Paranapanema [34,35]. Globally and countrywide, VL is considered a neglected disease that affects the poorest of the poor [16,32,33].

In Teodoro Sampaio, on a local scale, the periphery of sector 1 had the higher number of infected dogs, demonstrated by critical point hotspots. The first human case of VL in the city was diagnosed in this sector. From 2010 to 2018, 298 (7.9%) dogs were found

to be positive for CVL and 110 (36.9%) were euthanized. They were first found in sectors 1, 2, and 3 on the periphery, and in subsequent years, the hot spots spread to all urban areas. In the city, low levels of infected dogs were euthanized (36.9%). Similar to other cities of Pontal of Paranapanema, Teodoro Sampaio is surrounded by a high number of rural settlements [34,35]. Throughout the region, it is anecdotal that owners of dogs infected with VL hide their animals in these settings and may be disseminating the disease to rural areas. Although controversial, euthanasia is one of the most important actions supported by Brazilian Visceral Leishmaniasis Surveillance and Control Program in reducing the prevalence of *Leishmania (Leishmania) infantum chagasi* infection countrywide [36]. It has been shown that human epidemics of VL are usually preceded by or concomitant with CVL [9]. However, in Teodoro Sampaio, a diagnosis lag of 8 years between finding vectors, CVL, and HVL was found. Up to December 2020, only one individual had been registered. One possible explanation is that the city is located in the neighborhood of the Devil's Hill State Park, an endemic region for cutaneous leishmaniasis (CL) vectors [37,38], and the municipality is considered endemic for CL. There is some evidence that vectors have been present in the urban area for many years and a superposition CL/VL is occurring and a cross-reaction may be on course, protecting people from VL infection [39]. Supporting these findings, a VL serological survey of 159 individuals living in the urban area was carried out and only 2 (1.26%) individuals were positive by ELISA, with a probable false positive and/or cross-reaction. In addition, the mean residence times in the city was 23.86±15.27 years, increasing the possibility of these individuals being immunized against CL. Although VL-infected individuals do not have hallmark signs and symptoms, severe pancytopenia (thrombocytopenia, leukopenia, and anemia) and increased levels of hepatic enzymes may be considered in the differential diagnosis in

VL endemic areas [40]. In the population analyzed in this study, no laboratory findings symptomatic of chronic VL were found.

Shedding new light on the complex interactions occurring within the spread of VL in the western region and identifying the existence of spatial clusters of municipalities with increased vulnerability, Moran's I and LISA significance maps were created. Moran's I map identified a cluster of 11 municipalities with significant autocorrelation with CVL, and 5 municipalities were classified as high/high: Dracena, Ouro Verde, Tupi Paulista, Junqueirópolis, and Presidente Venceslau. Overall, 4 of these municipalities are considered the epicenter of the disease in which HVL was found in Dracena and Ouro Verde in 2005; Tupi Paulista in 2006, and Junqueirópolis in 2007 (Fig 4B). Furthermore, Dracena has a higher number of cases of HVL, varying from 58 to 176 infected individuals, followed by Ouro Verde, Tupi Paulista, and Junqueirópolis, varying from 17 to 57 infected individuals (Fig 4A). After all these years, despite the efforts made by the public authorities, Dracena remains the main focus of VL in western São Paulo. Taken together, these areas should be considered the target of local public policies and receive priority in surveillance actions within the scope of VL. As far as we know, this is the first effort to identify priority areas for VL in São Paulo State using Moran's I and LISA spatial analysis. In Brazil and worldwide, these tools have been used to identify vulnerable areas of parasitic, vector-borne neglected tropical diseases [13-15,41]. Moran's I and LISA techniques identified 27 priority areas for surveillance and control of VL in Belo Horizonte, an endemic area of Minas Geraes state [13]. In northeast municipalities, a VL Brazilian endemic region, spatial and space-time clusters of VL were identified in sertão and middle-north sub-regions, overlapping with high social vulnerability areas [14]. In Morocco, significant LISA clustering maps of human leishmaniasis have been identified in different provinces [41].

Different shortcomings should be considered in this study. The mean age of the population screened for VL was 43.50 years, and 72.33% were women. It is well known that VL affects mainly children and old people [40]. Thus, the population analyzed is not well representative of the disease. Municipal surveillance control programs of CVL were discontinued and there were difficulties in obtaining current records with a more accurate surveillance and response system to apply geospatial methods.

Conclusion

In RNHA11 mesoregion, in a regional scale VL is spreading with a progressive extension, from the north, the epicenter, to the south, from endemic to non-endemic areas. A cluster of municipalities in the epicenter showed a significant autocorrelation for CVL and should receive priority in surveillance actions within the scope of VL. Socioeconomic and environmental factors including big rivers and artificial lakes, highways hub and low HDI municipalities make actions against CVL and control of sand fly vectors difficult, fueling the epidemic and sustaining the endemic transmission of the disease. These findings further highlight the asymmetry of the western region compared with other regions of São Paulo state and add new knowledge on the spread of VL, contributing to the study of a neglected disease in a region of São Paulo, Brazil.

Author Contributions

Conceptualization: Luiz Euribel Prestes-Carneiro, Elivelton da Silva Fonseca, Regiane Soares Santana.

Data curation: Luiz Euribel Prestes-Carneiro

Formal analysis: Elivelton Silva Fonseca, Lourdes Aparecida Zampieri D'Andrea

Funding acquisition: Luiz Euribel Prestes-Carneiro, Elivelton da Silva Fonseca

Investigation: Regiane Soares Santana, Karina Briguenti Souza, Cristiane Oliveira Andrade, Lourdes Aparecida Zampieri D'Andrea, Ivete Rocha Anjolete

Methodology: Lourdes Aparecida Zampieri D'Andrea, Ivete Rocha Anjolete, Elivelton Silva Fonseca, Francisco Assis Silva, Edilson Ferreira Flores, Marcia Kaihara Meidas

Project principal investigator: Luiz Euribel Prestes-Carneiro

Field Supervision: Regiane Soares Santana

Validation: Luiz Euribel Prestes-Carneiro

Visualization: Elivelton Silva Fonseca, Francisco Assis Silva, Edilson Ferreira Flores, Fernanda Lussari

Writing – original draft: Luiz Euribel Prestes-Carneiro.

Writing – review & editing: Luiz Euribel Prestes-Carneiro, Regiane Soares Santana, Karina Briguenti Souza, Fernanda Lussari, Elivelton Silva Fonseca, Cristiane Oliveira Andrade, Lourdes Aparecida Zampieri D'Andrea, Francisco Assis Silva, Edilson Ferreira Flores, Ivete Rocha Anjolete

References

1. World Health Organization. Epidemiological situation. Cases and distribution of leishmaniasis**.** Available from: https://www.who.int/leishmaniasis/cases/en/..
2. Pan American Health Organization. Leishmaniasis: Epidemiological Report in the Americas, December 2020. Available from: https://iris.paho.org/handle/10665.2/53090
3. Ministério da Saúde (BR). Secretaria de Vigilância em Saúde. Vigilância em saúde no Brasil 2003|2019: da criação da Secretaria de Vigilância em Saúde aos dias atuais. Bol Epidemiol. Available from: http://www.rets.epsjv.fiocruz.br/biblioteca/vigilancia-em-saude-no-brasil-20032019-da-criacao-da-secretaria-de-vigilancia-em-saude
4. Centro de Vigilância Epidemiológica "Prof. Alexandre Vranjac". Leishmaniose visceral. Available from: http://www.saude.sp.gov.br/cve-centro-de-vigilancia-epidemiologica-prof.-alexandre-vranjac/areas-de-vigilancia/doencas-de-transmissao-por-vetores-e-zoonoses/agravos/leishmaniose-visceral/

5. Correa Antonialli SA, Torres TG, Paranhos Filho AC, Tolezano JE. Spatial analysis of American Visceral Leishmaniasis in Mato Grosso do Sul State, Central Brazil. J Infect. 2007; 54(5): 509- 514. doi: 10.1016/j.jinf.2006.08.004. PMID: 16979241

6. Cardim MF, Rodas LA, Dibo MR, Guirado MM, Oliveira AM et al. Introduction and expansion of human American visceral leishmaniasis in the state of Sao Paulo, Brazil, 1999-2011. Rev Saude Pública. 2013; 47(4): 691- 700. doi:10.1590/S0034-8910.2013047004454.

7. Prestes-Carneiro LE, Daniel LAF, Almeida LC, D'Andrea LZ, Vieira AG. Spatiotemporal analysis and environmental risk factors of visceral leishmaniasis in an urban setting in São Paulo State, Brazil. Parasit Vectors. 2019; 12(1): 251. doi:10.1186/s13071-019-3496-6. PMID: 31113445

8. Boton Pereira DH, Primo LS, Pelizari G, Flores E, Moraes-Vasconcelos D et al. Primary Immunodeficiencies in a Mesoregion of São Paulo, Brazil: Epidemiologic, Clinical, and Geospatial Approach. Front Immunol. 2020; 11: 862. doi:10.3389/fimmu.2020.00862. PMID: 32477349

9. D'Andrea LA, Fonseca ES, Prestes-Carneiro LE, Guimarães RB, Yamashita RC et al. The shadows of a ghost: a survey of canine leishmaniasis in Presidente Prudente and its spatial dispersion in the western region of São Paulo state, an emerging focus of visceral leishmaniasis in Brazil. BMC Vet Res. 2015; 11: 273. doi:10.1186/s12917-015-0583-6. PMID: 26503368

10. Alves MCGP, Matos MR, Reichmann ML, Dominguez MH. Estimation of the dog and cat population in the State of São Paulo. Rev. Saúde Pública, 2005; 39(6): 891-897. DOI: *10.1590/s0034-89102005000600004*. PMID: 16341397

11. Centro de Vigilância Epidemiológica (Manual de vigilância e controle da leishmaniose visceral Americana do Estado de São Paulo. 2006. Available from: https://pesquisa.bvsalud.org/portal/resource/pt/lis-LISBR1.1-22088
12. Pereira de Souza C, Ferreira da Silva P, de Carvalho Moreno M, Aparecida Zampieri D' Andrea L. Serviços de zoonoses e o seu papel na vigilância em saúde para leishmaniose visceral. Colloquium Vitae. 2019; 11(1): 24-32.
13. de Arruda RMF, Cardoso DT, Teixeira-Neto RG, Barbosa DS, Ferraz RK, et al. Space-time analysis of the incidence of human visceral leishmaniasis (VL) and prevalence of canine VL in a municipality of southeastern Brazil: Identification of priority areas for surveillance and control. Acta Trop. 2019; 197:105052. doi: 10.1016/j.actatropica.2019.105052. PMID: 31233726.
14. Ribeiro CJN, Dos Santos AD, Lima SVMA, da Silva ER, Ribeiro BVS et al. Space-time risk cluster of visceral leishmaniasis in Brazilian endemic region with high social vulnerability: An ecological time series study. PLoS Negl Trop Dis. 2021 .19;15(1): e0009006. doi: 10.1371/journal.pntd.0009006. PMID: 33465104;
15. Barbosa DS, Belo VS, Rangel ME, Werneck GL. Spatial analysis for identification of priority areas for surveillance and control in a visceral leishmaniasis endemic area in Brazil. Acta Trop. 2014; 131: 56-62. doi: 10.1016/j.actatropica.2013.12.002. PMID: 24342506.
16. Nunes BEBR, Leal TC, Paiva JPS, Silva LFD, Carmo RFD. et al. Social determinants of mortality due to visceral leishmaniasis in Brazil (2001-2015): an ecological study. Rev Soc Bras Med Trop. 2019; 53: e20190262. doi: 10.1590/0037-8682-0262-2019. PMID: 31859950

17. Galati EAB. Morfologia e taxonomia. In: Rangel EF, Lainson R, editors. Flebotomíneos do Brasil. Rio de Janeiro: Fundação Oswaldo Cruz; 2003. p. 23-52
18. Rangel O, Sampaio SM, Ciaravolo RM, Holcman MM. The distribution pattern of *Lutzomyia longipalpis* (*Diptera: Psychodidae*) in the peridomiciles of a sector with canine and human visceral leishmaniasis transmission in the municipality of Dracena, São Paulo, Brazil. Mem Inst Oswaldo Cruz. 2012; 107(2): 163-169. doi: 10.1590/s0074-02762012000200003. PMID: 22415253.
19. Thomaz-Soccol V, Gonçalves AL, Piechnik CA, Baggio RA, Boeger WA et al. Hidden danger: Unexpected scenario in the vector-parasite dynamics of leishmaniases in the Brazil side of triple border (Argentina, Brazil and Paraguay). PLoS Negl Trop Dis. 2018 6; 12(4): e0006336. doi: 10.1371/journal.pntd.0006336. PMID: 29624586
20. Pasquali AKS, Baggio RA, Boeger WA, González-Britez N, Guedes DC et al. Dispersion of *Leishmania (Leishmania) infantum* in central-southern Brazil: Evidence from an integrative approach. PLoS Negl Trop Dis. 2019 29; 13(8): e0007639. doi: 10.1371/journal.pntd.0007639. PMID: 31465422.
21. Cruz MF, Galati EA, Cruz CF. Ecological aspects of the sandfly fauna (*Diptera, Psychodidae*) in an American cutaneous leishmaniasis endemic area under the influence of hydroelectric plants in Paranapanema river, State of Paraná, Brazil. Rev Soc Bras Med Trop. 2012; 45(4): 430- 436. doi:10.1590/s0037-86822012000400003 PMID: 22930040
22. Souza MB. Influência de lagos artificiais no clima local e no clima urbano: estudo de caso em Presidente Epitácio, SP. Available from https://teses.usp.br/teses/disponiveis/8/8135/tde-08112010-162614/publico/2010_MarcosBarrosdeSouza.pdf

23. Guimarães AE, Gentile C, Alencar J, Lopes CM, de Mello RP. Ecology of *Anopheline (Diptera, Culicidae)*, malaria vectors around the Serra da Mesa Reservoir, State of Goiás, Brazil. 1-Frequency and climatic factors. Cad Saude Publica. 2004; 20(1): 291-302. doi: 10.1590/s0102-311x2004000100047. PMID: 15029332.

24. Melandri V, Alencar J, Guimarães AÉ. The influence of the area of the Serra da Mesa Hydroelectric Plant, State of Goiás, on the frequency and diversity of anophelines (*Diptera: Culicidae*): a study on the effect of a reservoir. Rev Soc Bras Med Trop. 2015; 48(1): 33-8. doi: 10.1590/0037-8682-0225-2014. PMID: 25860461.

25. Gomes Ade C, Paula MB, Natal D, Gotlieb SL, Mucci LF. Effects of flooding of the River Paraná on the temporal activity of *Anopheles (Nyssorhynchus) darlingi* Root (*Diptera: Culicidae*), at the border state of Mato Grosso do Sul and São Paulo, Brazil. Rev Soc Bras Med Trop. 2010; 43(5): 516-522. doi: 10.1590/s0037-86822010000500009. PMID: 21085861.

26. Galvis-Ovallos F, Casanova C, Sevá ADP, Galati EAB. Ecological parameters of the (S)-9-methylgermacrene-B population of the *Lutzomyia longipalpis* complex in a visceral leishmaniasis area in São Paulo state, Brazil. Parasit Vectors. 2017 30; 10(1): 269. doi: 10.1186/s13071-017-2211-8. PMID: 28558760

27. Reis LLD, Balieiro AADS, Fonseca FR, Gonçalves MJF. Visceral leishmaniasis and its relationship with climate and environmental factors in the State of Tocantins, Brazil, from 2007 to 2014. Cad Saude Publica. 2019; 35(1): e00047018. doi: 10.1590/0102-311x00047018. PMID: 30652815.

28. Fonseca EDS, Guimarães RB, Prestes-Carneiro LE, Tolezano JE, Rodgers MSM et al. Predicted distribution of sand fly (*Diptera: Psychodidae*) species involved in the

transmission of Leishmaniasis in São Paulo state, Brazil, utilizing maximum entropy ecological niche modeling. Pathog Glob Health. 2021; 1: 1-13. doi: 10.1080/20477724.2020.1870031. PMID: 33427124.

29. Silva RA, Santos FK, Sousa LC, Rangel EF, Bevilaqua CM. Ecology of *Lutzomyia longipalpis* and *Lutzomyia migonei* in an endemic area for visceral leishmaniasis. Rev Bras Parasitol Vet. 2014; 23(3): 320-327. doi: 10.1590/s1984-29612014068. PMID: 25271451.

30. Salomón OD, Quintana MG, Bezzi G, Morán ML, Betbeder E et al. *Lutzomyia migonei* as putative vector of visceral leishmaniasis in La Banda, Argentina. Acta Trop. 2010; 113(1): 84-87. doi: 10.1016/j.actatropica.2009.08.024. PMID: 19716797.

31. Fernandes WS, Borges LM, Casaril AE, Oliveira EF, Infran JOM, et al. Sandfly fauna (*Diptera: Psychodidae*) in an urban area, Central-West of Brazil. Rev Inst Med Trop Sao Paulo. 2017 24; 59: e54. doi: 10.1590/S1678-9946201759054. PMID: 28902295.

32. Alvar J, Yactayo S, Bern C. Leishmaniasis and poverty. Trends Parasitol. 2006; 22(12): 552- 557. doi: 10.1016/j.pt.2006.09.004 PMID: 17023215

33. Brandão E, Romero S, da Silva MAL, Santos FLN. Neglected tropical diseases in Brazilian children and adolescents: data analysis from 2009 to 2013. Infect Dis Poverty. 2017 3; 6(1): 154. doi: 10.1186/s40249-017-0369-0. PMID: 29096720

34. Prestes-Carneiro LE, Rubinsky-Elefant G, Ferreira AW, Araujo PR, Troiani C et al. Seroprevalence of toxoplasmosis, toxocariasis and cysticercosis in a rural settlement, São Paulo State, Brazil. Pathog Glob Health. 2013; 107(2): 88- 95. doi: 10.1179/2047773213Y.0000000079 PMID: 23683335

35. Prestes-Carneiro LE, Santarém V, Zago SC, Miguel NA, Zambelli SF et al. Sero-epidemiology of toxocariasis in a rural settlement in São Paulo state, Brazil. Ann Trop Med Parasitol. 2008; 102(4): 347- 356. doi: 10.1179/136485908X278801 PMID: 18510815

36. da Rocha ICM, Dos Santos LHM, Coura-Vital W, Cunha GMR, Magalhães FC et al. Effectiveness of the Brazilian Visceral Leishmaniasis Surveillance and Control Program in reducing the prevalence and incidence of *Leishmania infantum* infection. Parasit Vectors. 2018; 11(1): 586. doi: 10.1186/s13071-018-3166-0 PMID: 30419944

37. Alessi CA, Galati EA, Alves JR, Corbett CE. American cutaneous leishmaniasis in the Pontal of Paranapanema - SP, Brazil: ecological and entomological aspects. Rev Inst Med Trop Sao Paulo. 2009; 51(5): 277- 282. doi: 10.1590/s0036-46652009000500008 PMID: 19893981

38. Condino ML, Sampaio SM, Henriques LF, Galati EA, Wanderley DM et al. American cutaneous leishmaniasis: sandflies from the transmission area in the town of Teodoro Sampaio, the southeastern region of São Paulo state, Brazil. Rev Soc Bras Med Trop. 1998; 31(4): 355-360. doi:10.1590/s0037-86821998000400004 PMID: 9662962

39. Fonseca ES, D'Andrea LA, Taniguchi HH, Hiramoto RM, Tolezano JE et al. Spatial epidemiology of American cutaneous leishmaniasis in a municipality of west São Paulo State, Brazil. J Vector Borne Dis. 2014; 51(4): 271- 275. PMID: 25540957

40. Prestes-Carneiro LE, Spir PRN, Fontanesi M, Garcia KGP, Assis FA et al. Unusual manifestations of visceral leishmaniasis in children: a case series and its spatial dispersion in the western region of São Paulo state, Brazil. BMC Infect Dis. 2019; 19(1): 70. doi: 10.1186/s12879-018-3652-1 PMID: 30658589

41. Sadeq M. Spatial patterns and secular trends in human leishmaniasis incidence in Morocco between 2003 and 2013. Infect Dis Poverty. 2016 11; 5(1): 48. doi: 10.1186/s40249-016-0135-8. PMID: 27164836.

The revised manuscript was sent to a native speaker: Our ref: Job ID 2094 Language edit Revised Article: Cases and distribution of visceral leishmaniasis in western São Paulo: a neglected disease in this region of Brazil

Figure 1 Road network

Click here to access/download;Figure;Figure 1 Road network.tif

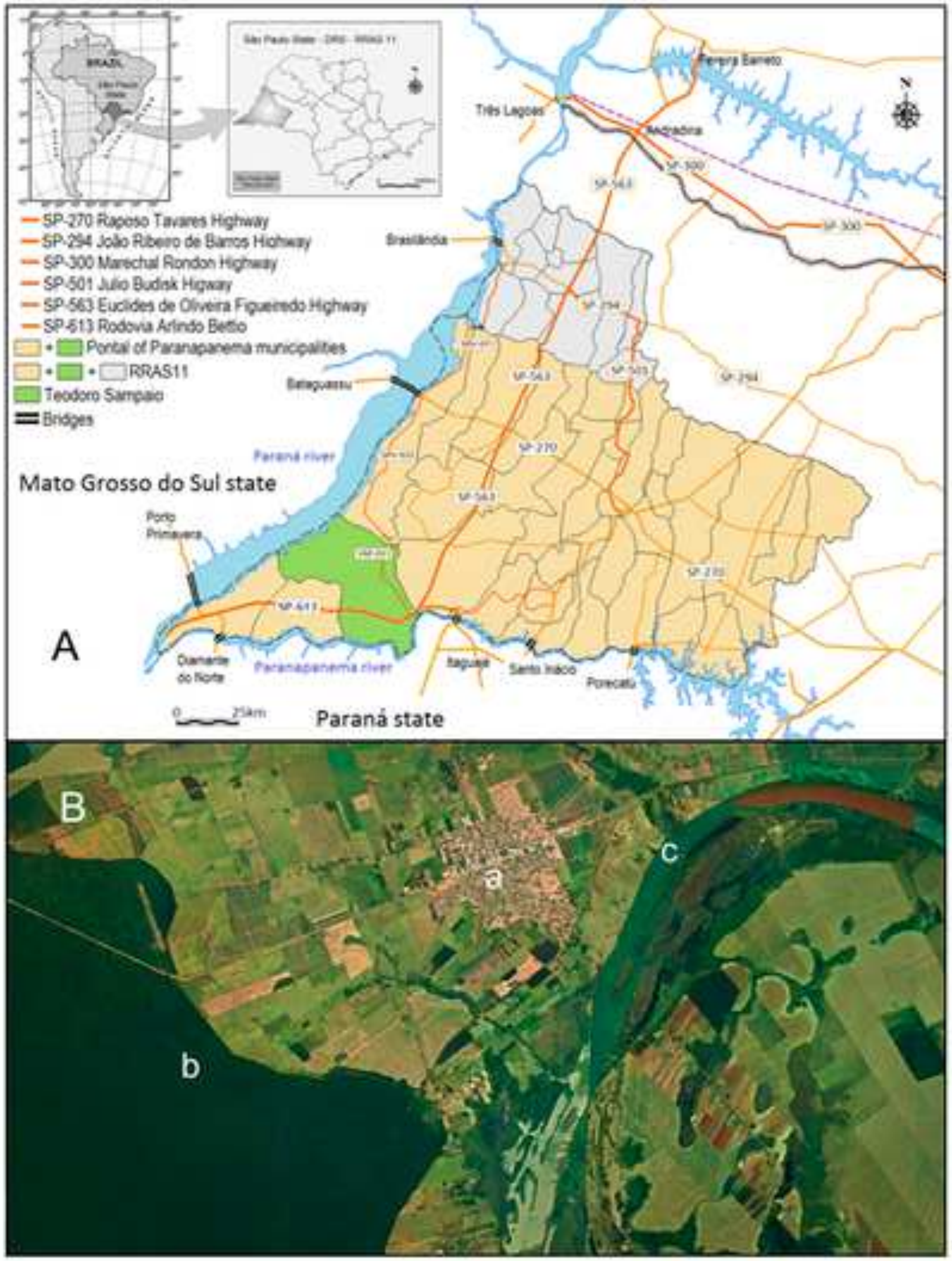

Figure 2 Spatial distribuion of HDI

Click here to access/download;Figure;Figure 2 Spatial distribution HDI.tif

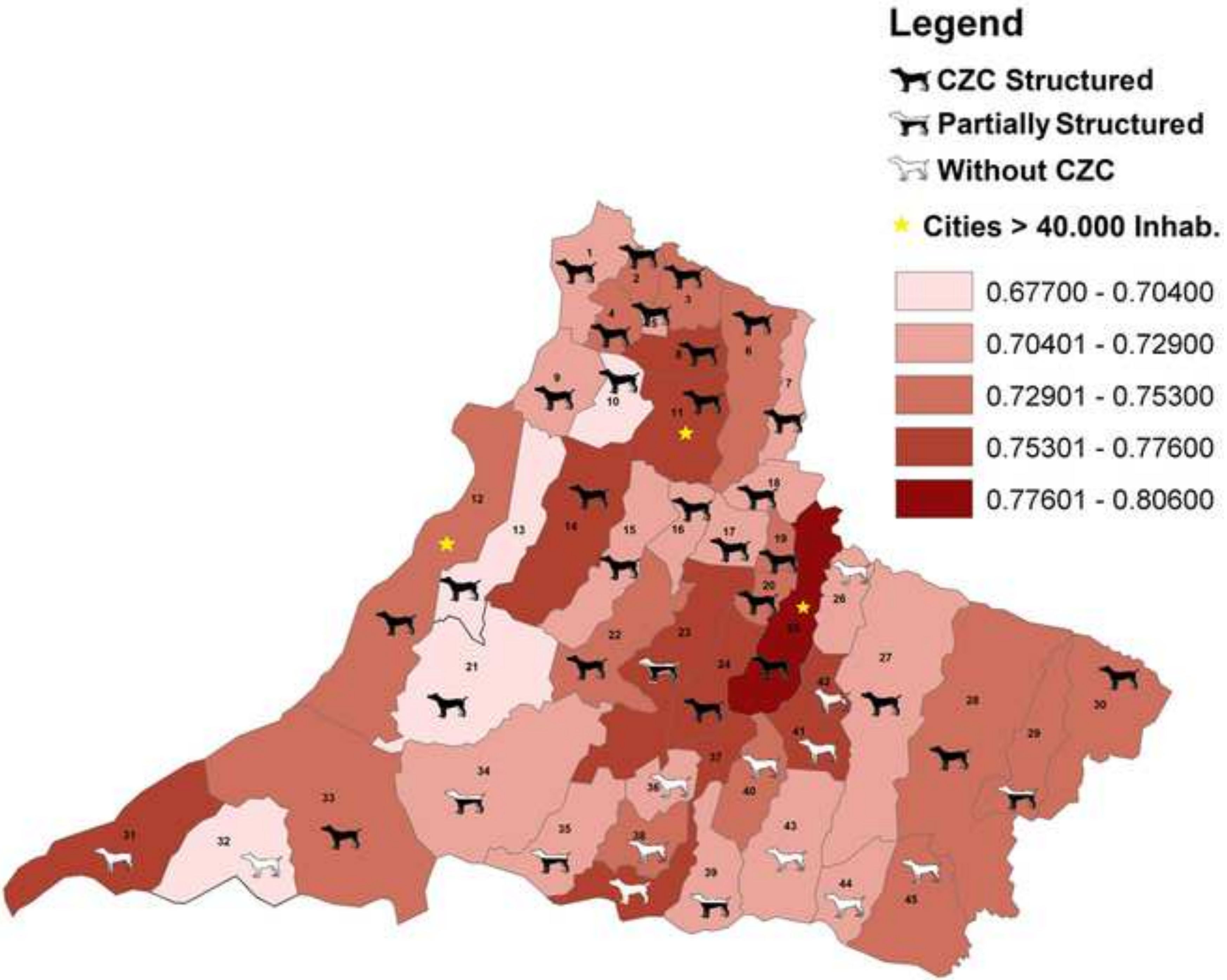

Figure 3 Spatial distribution HDIFigure 3 Spatial distribution of high-risk clusters tiff
Click here to access/download;Figure;Figure 3 Spatial distribution of high-risk clusters tiff.tif

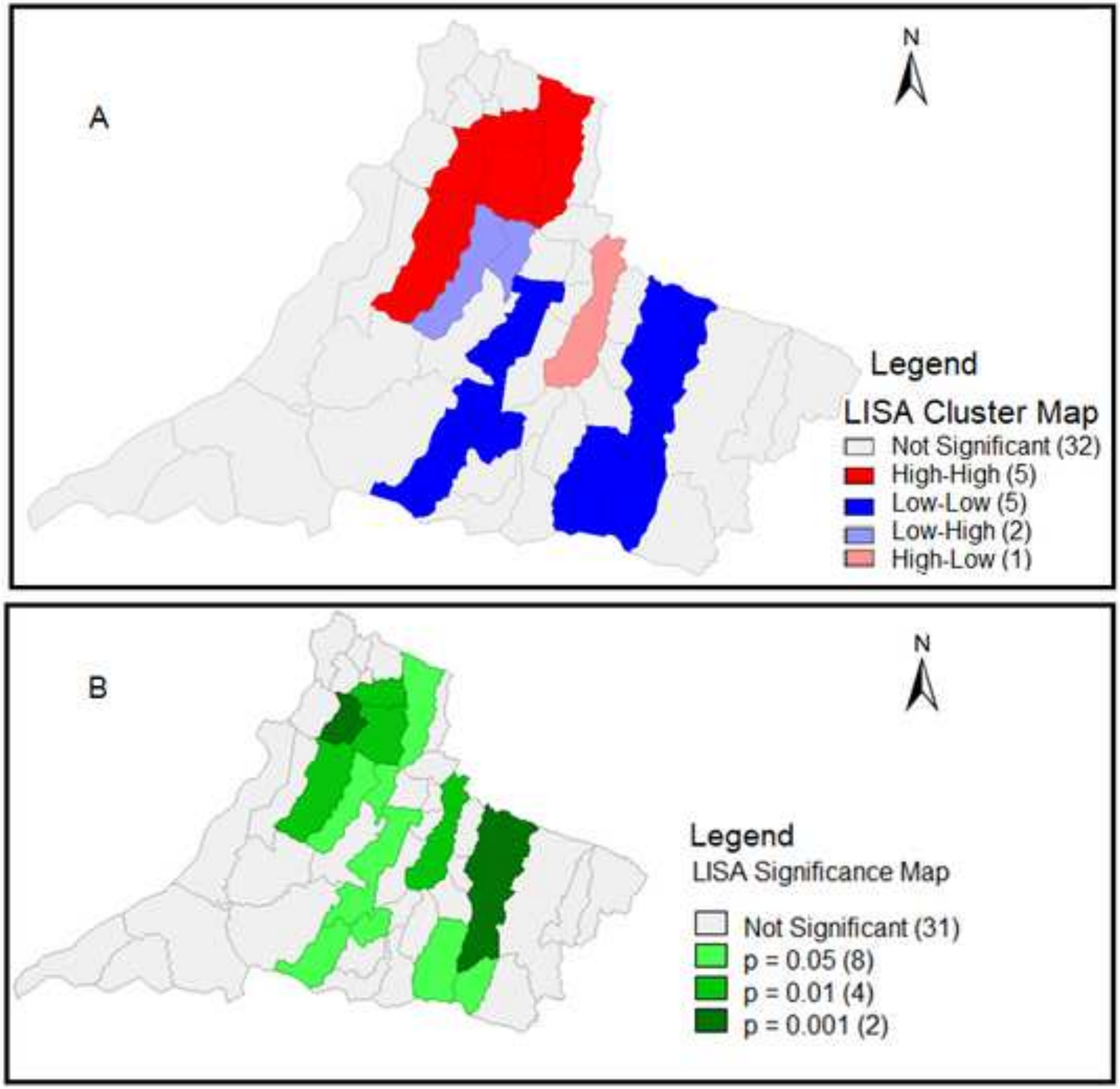

Figure 4 Geographic distribution of HVL 300dpi 10 MB

Click here to access/download;Figure;Figure 4 Geographic distribution of HVL 300dpi 10 MB.tiff

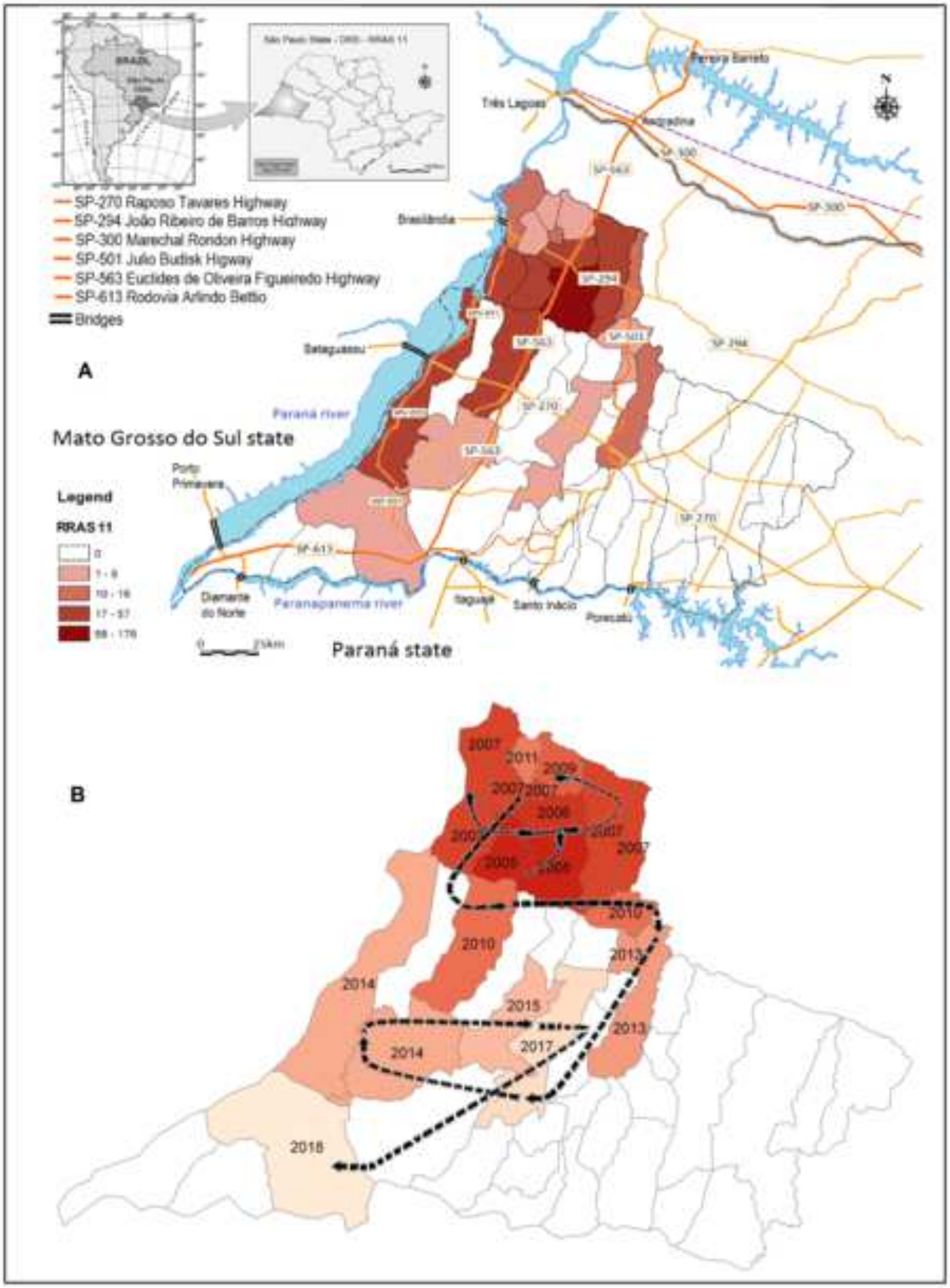

Figure 5 Correlation between HDI and VL

Click here to access/download;Figure;Figure 5 Correlation between HDI and VL .tif

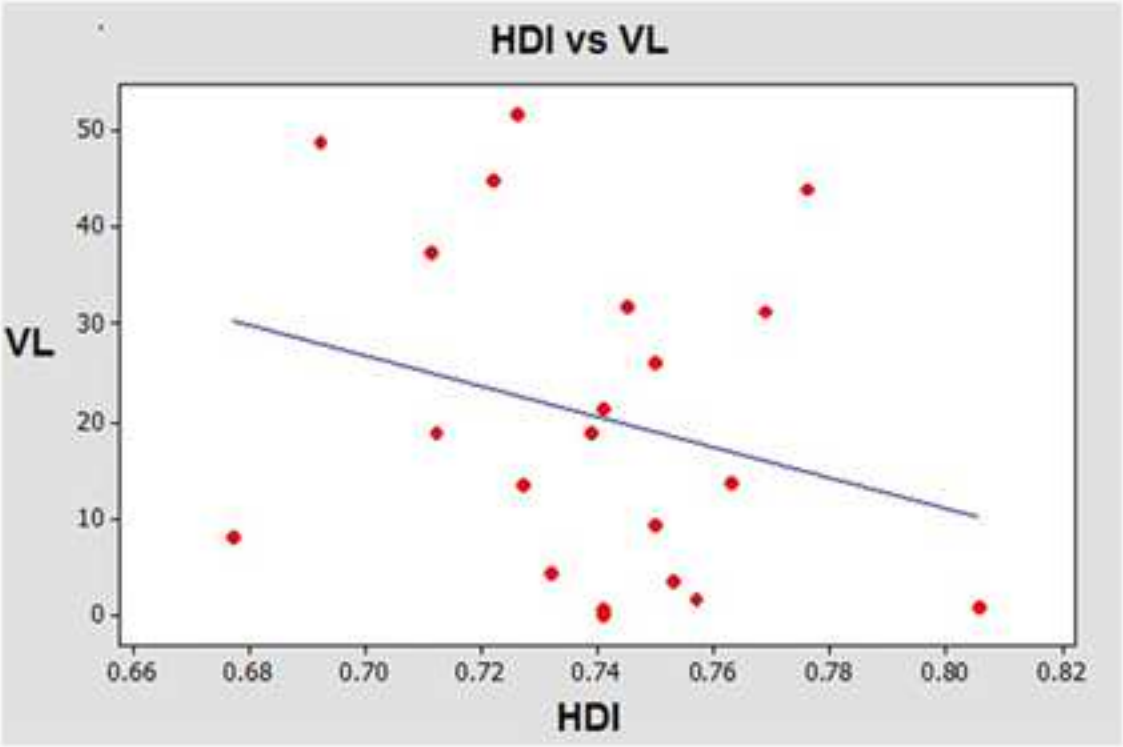

Figure 6 Kernel of spatiotemporal distribution

Click here to access/download;Figure;Figure 6 Kernel of spatiotemporal distribution .tif

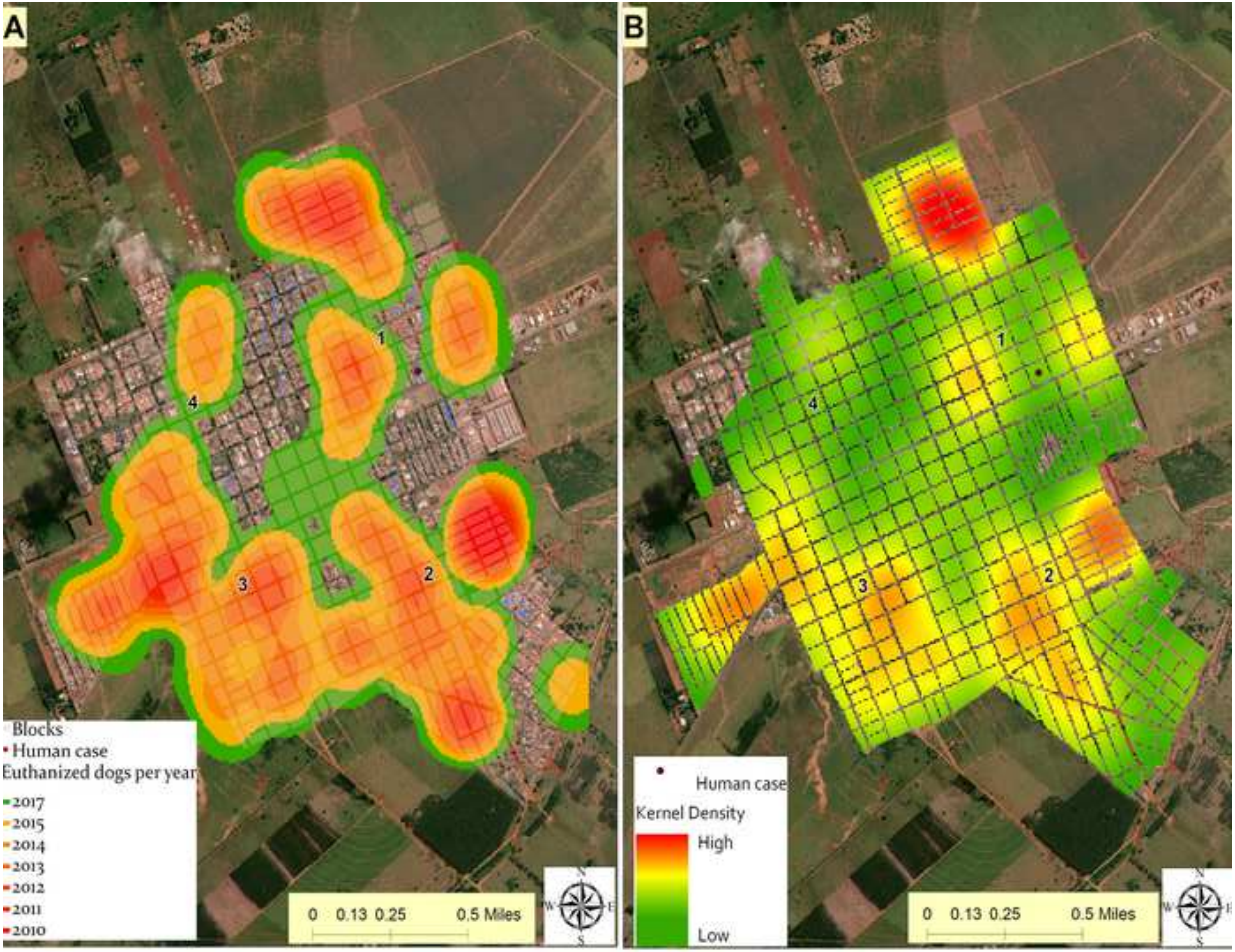

Figure 7 Socioeconomic_Environment changes. tif

Click here to access/download;Figure;Figure 7 Socioeconomic_Environment changes. tif.tif

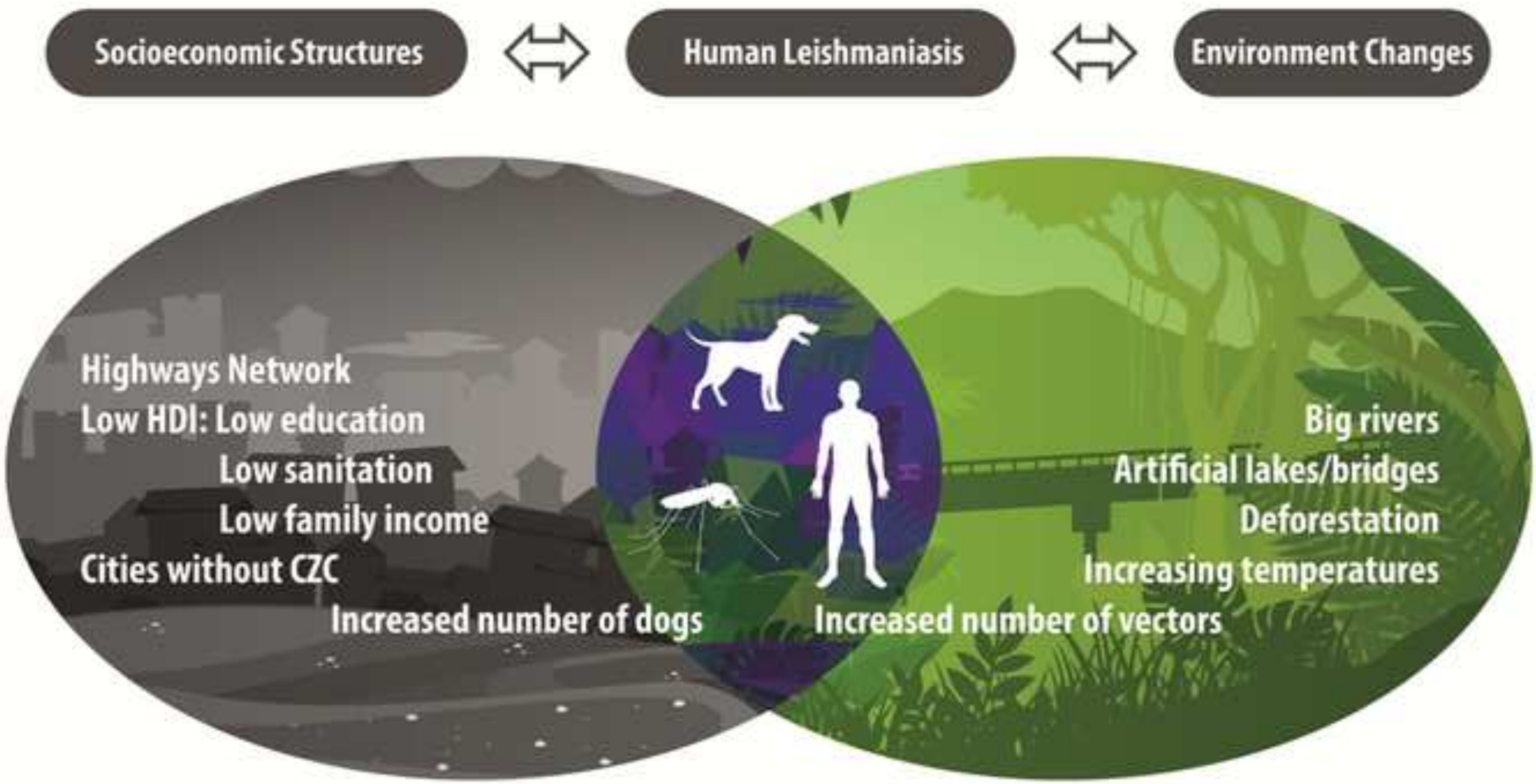

Answers to the PDF comments: Reviewer 2 Pages 8-12

Reviewer's Responses to Questions

The revised manuscript was sent to a native speaker: Language edit Revised Article: Cases and distribution of visceral leishmaniasis in western São Paulo: a neglected disease in this region of Brazil

Key Review Criteria Required for Acceptance?</br></br>
As you describe the new analyses required for acceptance, please consider the following:</br></br>

Methods</br></br>
-Are the objectives of the study clearly articulated with a clear testable hypothesis stated?</br>
-Is the study design appropriate to address the stated objectives?</br>
-Is the population clearly described and appropriate for the hypothesis being tested?</br>
-Is the sample size sufficient to ensure adequate power to address the hypothesis being tested?</br>
-Were correct statistical analysis used to support conclusions?</br>
-Are there concerns about ethical or regulatory requirements being met?</br></br>

Reviewer #1 – Dear Dr. Thanks for your time and valuable contribution to our manuscript. We tried to fill in all the issues raised in your comments, however, if new amendments are necessary, we will try to make them.

The manuscript "The burden of visceral leishmaniasis in western São Paulo: a neglected disease in this region of Brazil " aims to determine the LV burden in 29 municipalities of the Regional Health Assistance Network (RNHA11), west of São Paulo, in a multiscale approach centered on the city of Teodoro Sampaio. However, the authors do not propose any hypothesis in the introduction section. They also do not raise questions about the entry and spread of the disease in the region. In the description it is necessary to be more clear which data were obtained from databases and which were performed by the authors.

Answer. The manuscript was re-structured according to your suggestion and comments.

In the Introduction section*, a description of the entry and dispersion of VL was ruled out. Page 4 - Lines 69-82*

A hypothesis was performed on the role of socioeconomic and environmental risk factors in the spreading of Lu. longipalpis, infected dogs, and human visceral leishmaniasis in the western region of São Paulo state. Page 4 – Lines 83-92.

In addition, following your observation, we divided the methodology and results on a regional scale with data obtained in public databases, and at the local scale, focusing on the city of Teodoro Sampaio, with data performed by the authors. Page 4 – Lines 92-96.

In the Methods section

***First**, an approach on a regional scale, using public databases was focused, as follow: Study design and setting:*

Page 5 – Lines 101-116: Regional characteristics; Page 6 – Lines 117-141: Local characteristics

Data on the presence of Lu. longipalpis in the municipalities of RNHA11 Page 8 – Lines 173-183

Canine and human visceral leishmaniasis and Center of Zoonosis Control in the municipalities of RNHA11mesoregion – Page 8 – Lines 184-209

Mapping the cluster of identification of canine visceral leishmaniasis in RNHA11 mesoregion – Page 9 – Lines 210-228

Mapping the Human Development Index in the RNHA11 mesoregion: Page 10 – Lines 229-242

***Second,** we presented the results at a local scale focusing in the municipality of Teodoro Sampaio, a representative municipality of Pontal of Paranapanema, and of the RNHA-11, which data were performed by the authors, as follow:*

Entomological survey in Teodoro Sampaio: Page 12 – Lines 243-262

Canine visceral leishmaniasis survey in Teodoro Sampaio: Page 12 – Lines 263-287

Human visceral leishmaniasis in Teodoro Sampaio: Page 12-13 – Lines 288-294

Human visceral leishmaniasis serological survey and laboratory diagnosis in Teodoro Sampaio: Page 14 – Lines 295-318

Reviewer #2: *Dear Dr. We are grateful for your correction, comments, and suggestions in the PDF file of the manuscript. We are aware that the time you spent on this work is precious. We hope to fit all the issues raised in your comments. The detailed answers and corrections of your comments are in the PDF file brackets.*

The objectives of the study are clearly articulated. The study design is a little bit confuse for the reader. The population studied (human, vectors and dogs) are described in a disorderly way, in my opinion. It becomes a bit difficult to read the paper. The manuscript contributes with original and useful information related to several aspects of leishmaniasis in a poor area**.**

Answer: According to your suggestion, to be more understandable, the Study Design, Methods, and Results sections were amended as follow:

***In the Introduction section**, a description of the entry and dispersion of VL was ruled out. Page 4 - Lines 69-82*

A hypothesis was performed on the role of socioeconomic and environmental risk factors in the spreading of Lu. longipalpis, infected dogs, and human visceral leishmaniasis in the western region of São Paulo state. Page 4 – Lines 83-92.

In addition, following your observation, we divided the methodology and results on a regional scale with data obtained in public databases, and at the local scale, focusing on the city of Teodoro Sampaio, with data performed by the authors. Page 4 – Lines 92-96.

In the Methods section

***First**, an approach on a regional scale, using public databases was focused, as follow: Study design and setting:*

Page 5 – Lines 101-116: Regional characteristics; Page 6 – Lines 117-141: Local characteristics

Data on the presence of Lu. longipalpis in the municipalities of RNHA11 Page 8 – Lines 173-183

Canine and human visceral leishmaniasis and Center of Zoonosis Control in the municipalities of RNHA11mesoregion – Page 8 – Lines 184-209

Mapping the cluster of identification of canine visceral leishmaniasis in RNHA11 mesoregion – Page 9 – Lines 210-228

Mapping the Human Development Index in the RNHA11 mesoregion: Page 10 – Lines 229-242

***Second,** we presented the results at a local scale focusing in the municipality of Teodoro Sampaio, a representative municipality of Pontal of Paranapanema, and of the RNHA-11, which data were performed by the authors, as follow:*

Entomological survey in Teodoro Sampaio: Page 12 – Lines 243-262

Canine visceral leishmaniasis survey in Teodoro Sampaio: Page 12 – Lines 263-287

Human visceral leishmaniasis in Teodoro Sampaio: Page 12-13 – Lines 288-294

Human visceral leishmaniasis serological survey and laboratory diagnosis in Teodoro Sampaio: Page 14 – Lines 295-318

Reviewer #3: Dear Reviewer: Thanks for considering our manuscript for revision. Your expertise will certainly contribute to the improvement of the article. We tried to fill in

all the issues raised in your comments, however, if new amendments are necessary we will try to make them.

Are the objectives of the study clearly articulated with a clear testable hypothesis stated? No, the authors used data from other public bodies as part of the methodology. For example: Data from the Supervision in Control of Epidemic- (SUCEN), Center of Regional Laboratory of the Adolfo Lutz Institute of Presidente Prudente V (CRL-CBSALI-PPV), National System of Diseases Notification (SINAN), Epidemiological Surveillance Center (CVE-SP). Considering each institution, the methodology used by them to obtain their results is different. If you need to redo this search. It will be necessary to obtain all this data and more that they have and if it is possible to do so.

Answer: To get the manuscript more understandable, we divided the methodology and the results on a regional scale, using databases, obtained from public agencies, focusing on vectors, canine visceral leishmaniasis, human leishmaniasis, and Human Developing Index. Using Teodoro Sampaio, considered the Pontal of Paranapanema and one of the poorest regions of São Paulo state, on a local scale, data obtained by the authors on the entomological survey, canine and human visceral leishmaniasis was discussed. Furthermore, on a regional scale, we added a statistical analysis using Moran's I and LISA methods to identify spatial autocorrelation and to generate cluster maps as priority areas for implementing surveillance and control actions.

At the regional scale, public databases were provided by:

Data of *Lu. longipalpis* was provided by superintendencia-de-controle-de-endemias (SUCEN). SUCEN is a public agency of São Paulo state with the mission of coordinating, monitor and evaluate vector surveillance and control actions, intermediate hosts, and venomous arthropods, as well as producing and disseminate scientific-technical knowledge and share technologies for health surveillance. The data obtained from SUCEN was coordinated by the director of SUCEN in Presidente Prudente: Ivete da Rocha Anjolete.

Data of canine visceral leishmaniasis. Adolfo Lutz Institute (ALI) is the public former laboratory in São Paulo state responsible for the laboratory confirmation of canine visceral leishmaniasis in the whole state. At RNHA11, the ALI coordinates the actions on laboratory diagnosis in all municipalities. Data on CVL and the presence of Zoonosis Center Control was coordinated by Lourdes D'Andrea Zampieri.

Data on human visceral leishmaniasis. HVL data was obtained from the Epidemiological Surveillance Center Alexandre Vranjack (CVE). In São Paulo state, CVE is the public agency responsible by technical guidance on epidemiological investigation and control of diseases of interest to public health; water and foodborne diseases, respiratory diseases, vector-borne diseases and zoonoses, leprosy, tuberculosis, health ophthalmology center, hepatitis, chronic diseases. CVE coordinates the cases of human visceral leishmaniasis that occurred in the state and publishes a monthly bulletin that is publicly available.

Environment data: Data on rivers, damns, artificial lakes and highways were accessed from Instituto Brasileiro de Geografia e Estatística.

Socioeconomic risk factors: Data on Human Developing Index was obtained from Fundação Sistema Estadual de Análises de Dados (SEADE).

On a local scale, in Teodoro Sampaio, data was generated by the authors. The entomological survey was conducted by Ivete da Rocha Anjolete, canine visceral leihsmaniasis was conducted by Lourdes D'Andrea Zampieri, human VL survey was conducted by Regiane Soares, Karina Briguenti Souza, Cristiane Oliveira Andrade, and Luiz Euribel Prestes Carneiro. The ELISA tests were performed by Lourdes D'Andrea Zampieri and laboratory tests performed in the HVL survey individuals were performed by Marcia Kaihara Meidas.
.

-Does the analysis presented match the analysis plan?</br>
-Are the results clearly and completely presented?</br>
-Are the figures (Tables, Images) of sufficient quality for clarity?</br></br>

Reviewer #1: In my opinion, the sections "Results" and "Materials and Methods" need to be improved. The sequence of data and a title are not very didactic for the readers. They must be put in the same order.

Answer: We re-organized the Methods and Results sections putting them in the same sequence. First at a regional scale with data on the 45 municipalities of the RNHA-11 mesoregion and Second, on local scale focusing on the urban area of Teodoro Sampaio, a representative municipality of the western region of São Paulo state.

The figures from 1 to 5 are correct. However, the Figure 6 does not make much sense. It should be deleted or give more meaning to the readers.
In the reference section, many scientific words are not italicized.

Answer: Figure 6 was re-defined and helps the readers to understand the socioeconomic and the environmental changes that is occurring in the western region. Taken together, certainly, they contribute to the spreading of vectors, infected dogs, and humans VL in RNHA11 mesoregion of São Paulo state.

The reference section was updated and corrected according to your observation.

Reviewer #2:

In my opinion, some part of the text that appear on the results must be moved to Material and Methods.

Answer: The subsection Potential dispersion routes of infection: RNHA11 mesoregion and Pontal of Paranapanema hub was resumed and moved to the Methods section: Study design and settings - Page 7 – Lines 141-152

Reviewer #3:

Does the analysis presented match the analysis plan?
Yes

-Are the results clearly and completely presented?

No, there was no entomological research, but a collection of sandflies, the results being incomplete.

Answer: Your comments were addressed to one of the researchers: Ivete da Rocha Anjolete, the director of the regional department of SUCEN in Presidente Prudente, who conducts the entomological surveys in the 45 municipalities of RNHA11. She revised the text and corrected the information. Page 12 – Lines 244-247.

Table 1 - showing the entomologic survey in Teodoro Sampaio conducted by the authors was corrected and completed.

The importance of knowing the phlebotomine fauna in the region was pointed out and data on Lu. migonei and Lu. cruzi in the region were added in the discussion section: Page 29 – Lines 618-625.

-Are the figures (Tables, Images) of sufficient quality for clarity?

The exception of table 1. The other tables and images are of sufficient quality for clarity

Answer: Table 1 was redefined as suggested, and with the objective of improving the resolution, figure B was deleted.

Conclusions</br></br>
-Are the conclusions supported by the data presented?</br>
-Are the limitations of analysis clearly described?</br>
-Do the authors discuss how these data can be helpful to advance our understanding of the topic under study?</br>
-Is public health relevance addressed?</br></br>

Reviewer #1: The manuscript is of interest to public health. However, the authors should improve the discussion section. The authors should be more concise and didactic.
Answer: The discussion was re-written and shortened according to your suggestion. Only issues addressing VL dispersion in RNHA11 and Teodoro Sampaio were highlighted. Furthermore, the more important results on vectors, canine visceral leishmaniasis, socioeconomic and environmental data were compared with the scientific literature.

Reviewer #2: Part of the discussion and conclusions must be supported by more references.

Answer: The discussion and bibliography were updated and corrected according to your observation.

Reviewer #3: Are the conclusions supported by the data presented?

Yes

Are the limitations of analysis clearly described?

Yes

Do the authors discuss how these data can be helpful to advance our understanding of the topic under study?

No, they report that this study may contribute new knowledge to the study of this neglected disease. They cite environmental, social and economic conditions as factors that can hinder actions against CVL and disease vectors, thus sustaining this disease in an endemic area.

Answer: We tried to highlight the contributions of our data to new knowledge to study this neglected disease in the western region at the regional and local scale. Page 4 – Lines 83-96.

Furthermore, Moran's I and LISA cluster maps were created to identify priority areas for implementing surveillance and control actions.

At the regional scale, combining data of VL with environmental and socioeconomic risk factors allows constructing the potential dispersion routes and the municipalities with higher socioeconomic and environmental vulnerability. Knowing dispersion routes is essential to develop preventive measures to control the disease and restrain its future dispersion. Knowing the environmentally/socioeconomic related vulnerable municipalities may transform governmental public health and community education actions more effective in controlling the endemic transmission of the disease in the dominium of RNHA11. In Teodoro Sampaio, most of the infected dogs were found in the periphery and low levels were euthanized. They may be disseminating the disease to rural and neighboring áreas free of VL. Surveys conducted in vectors, reservoirs, and humans can be used by local and regional authorities, state and countrywide for understanding the VL transmission, its fragilities and taking coordinated measures to control the disease at the local and regional scale.

-Is public health relevance addressed?

Yes

Answers to the PDF comments: Reviewer 2

Abstract
It would not be **cases** of visceral leishmaniasis:

Answer: The title was changed according to your suggestion: Cases and distribution of visceral leishmaniasis in western São Paulo: a neglected disease in this region of Brazil

How many municipalities are there? include the number of municipalities.
Answer: 45 municipalities
Answer: The number of municipalities were included as follow: Lines 24-34: To study this neglected disease on a regional scale, we describe the spread of VL in 45 municipalities of the Regional Network for Health Assistance11(RNHA11).

Line 34. For the local study in Teodoro Sampaio, an entomological survey from 2009 to 2015. *Inform, the location of the collection (intradomicile, peridomicile, forest, etc.), the start and end time of the collections, were the catches fortnightly or monthly?*
35. 2015, a canine visceral leishmaniasis (CVL) survey from 2010 to 2018 by enzyme-linked immunosorbent assays ELISA, and a human serum survey in 2018 were carried out. *What was the methodology used to survey CVL cases?*
Answer: Lines 32-34 In Teodoro Sampaio, monthly peridomicile sand flies collection; ELISA, IFAT and Rapid Test serological canine VL (CVL); and an ELISA human VL (HVL) serum surveys were carried out.

Lines 37,38. From 2005 to 2018, *Lutzomyia longipalpis* was found in 38 (77.8%), CVL in 68%, and HVL in 42.2% of the municipalities. *You are confused, please, in the entomological survey carried out from 2005 to 2018, where was found Lu longipalpis, is it included the research done in Teodoro Sampaio from 2009 to 2015, by the authors?*
Answer: The methodology was corrected as follow: Lines 24-34
To study this neglected disease on a regional scale, we describe the spread of VL in 45 municipalities of the Regional Network for Health Assistance11(RNHA11). Environmental, human VL (HVL), and canine VL (CVL) cases, Human Development Index, and Lutzomyia longipalpis databases were obtained from public agencies. Global Moran's I index and local indicators of spatial association (LISA) statistics were used to identify spatial autocorrelation and to generate maps for the identification of VL clusters. On a local scale, we determined the spread of VL in the city of Teodoro Sampaio, part of the Pontal of Paranapanema. In Teodoro Sampaio, monthly peri-domicile sand fly collection; ELISA, IFAT and Rapid Test serological CVL; and ELISA human HVL serum surveys were carried out.

Answer: A detailed information on the entomological survey was added to Methods and Results sections: Lines 245-248: In the urban area of Teodoro Sampaio, the phlebotomine sand fly entomological survey began in 2000, monthly, from February to October during the most favourable periods for detecting the presence of the vector. The survey ended in October 2010 when Lu. longipalpis was found in all areas.

Line185. Four traps were installed for three nights in each sector. *Were these collections fortnightly or monthly?*
Answer: Lines 245-248: In the urban area of Teodoro Sampaio, the phlebotomine sand fly entomological survey began in 2000, ***monthly****, from February to October during the most favourable periods for detecting the presence of the vector. The survey ended in October 2010 when Lu. longipalpis was found in all areas.*

Line 258: Monthly, four traps were installed for three nights in each sector.
Monthly, from February to October during the most favorable periods for detecting the presence of the vector.

Lines 190-192. In 2009 when the survey started in the urban area of Teodoro Sampaio, the traps were installed in four areas concomitantly. In 2010, the entomology survey ended after the vector was found in all areas. *In the summary of the article, it appears that an entomological survey was carried out from 2009 to 2015. However, in this paragraph it is informed that the entomological research, in the urban area of Teodoro Sampaio, took place from 2009 to 2010. In this period, in addition to Lu. longipalpis, what species were found? The literature points out, the meeting of Lu cruzi as an LV vector in Corumbá, Mato Grosso. The encounter of Lu migonei naturally infected with Leishmania infantum chagasis in Pernambuco. The importance of knowing the phlebotomine fauna is supported by the findings mentioned above. From 2011 to 2015, was there a collection? And where was it done?*
Entomological research has to be done at least in two years, in the case in question what happened was a collection of sandflies.

Answer: We separated data from regional scale joining data of the 45 municipalities of RNHA-11 obtained from public databases.

Lines 175-185: Data on the presence of Lu. longipalpis in the municipalities of RNHA11

In RNHA11, data on phlebotomine sand flies identified in entomological collections were obtained from SUCEN in the period from 2000 to 2019. Entomologic surveys are proposed by the Manual of Surveillance and Control of Visceral Leishmaniasis of São Paulo (VLCPSP) [11], aiming to monitor the distribution of Lu. longipalpis in silent non-receptive, non-vulnerable municipalities, and silent non-receptive vulnerable municipalities as defined by the absence of confirmed autochthonous cases of HVL and CVL, without the presence of the chosen vector or not by estimated values of the distance to characterize the vulnerability. In these municipalities, an entomologic survey is recommended for the detection of the presence of Lu. longipalpis and to provide information on its distribution, allowing identification of the risk areas where control measures should be intensified [11].

Lines 244-248 Entomological survey in Teodoro Sampaio

Entomological survey in Teodoro Sampaio

In the urban area of Teodoro Sampaio, the phlebotomine sand fly entomological survey began in 2000, monthly, from February to October during the most favourable periods for detecting the presence of the vector. The survey ended in October 2010 when Lu. longipalpis was found in all areas.

We are in line with your opinion on the importance of knowing the phlebotomine fauna in the region and new data was added to the discussion section. Lines 600-607: In Teodoro Sampaio, although an entomological survey started in 2000, Lu. longipalpis sand flies were found only in 2010. It is well known that other phlebotomines, such as Lu. migonei, may be permissive vectors of VL in areas where Lu. longipalpis has not been recorded, but human or canine cases do occur [28-30]. According to the SUCEN database, in the period from 2000 to 2010, Lu. cruzi a VL vector found in Central-West of Brazil [31], and Lu. migonei were not identified in Teodoro Sampaio urban area. Lu. migonei was found in the wild forest of the Devil's Hill in research carried out for an LT investigation in 1994, 1995, and 1996.

Line 208 - Publication reference

Answer: suspicious for CVL, as previously published [9].

Line 278 was obtained in SEADE, *This acronym is appearing for the first time. Write in full and put the acronym in brackets*
Answer: Fundação Sistema Estadual de Análise de Dados

Line 302 Maps were constructed using a geographic information system. *Which geographic information system?*

Answer: Line 355 Maps were constructed using ArcGIS 10.7 and ArcGis Pro.

Lines 305-325 - *This text describes the geography and highways of the studied site, which are already known public information. Citing endemic VL regions. And they were put as results. What are the results? What is intended proves with this description. I believe that, it would be better if it were placed in the "Material and Method. I did not understand. Please delete or rewrite.*

Answer: The text was shortened and sent to Methods section; Study design and settings. Lines 143-162: **Potential dispersion routes of VL infection in RNHA11 mesoregion**

RNHA11 is connected by two important radial highways: (1) the Marechal Candido Rondon (SP 300) linking two VL endemic regions: Três Lagoas, on the border of MS and Araçatuba-Bauru in the center-northeastern region of São Paulo; (2) the Euclides Figueiredo highway (SP 563) linking the VL endemic regions of Pereira Barreto, Ilha Solteira, and Andradina to Dracena, one of the most important cities of RNHA11, also with the highest prevalences in VL. Secondary highways also link the RNHA11 from north to south in direction to Paraná state, a region free of VL so far. Teodoro Sampaio is connected by a network of radial and secondary highways. One of the most important is the secondary vicinal highway (VSP-031; VSP-035) connecting the endemic regions of Presidente Epitácio and Panorama, bordering the Paraná River to Teodoro Sampaio (Fig1A)

Line 365: **Entomological survey of Lu. longipalpis in the urban area of Teodoro Sampaio.** Entomological research must be carried out in at least two years, with the aim of comparing the results found. Collections were made in the urban area of Teodoro Sampaio. And only Lu. longipalpis was collected.? What is the phlebotominic fauna of the region? It would be interesting to record this data.

Answer: The text was corrected as follow: Lines 245-249: In the urban area of Teodoro Sampaio, the phlebotomine sand fly entomological survey began in 2000, monthly, from February to October during the most favourable periods for detecting the presence of the vector. The survey ended in October 2010 when Lu. longipalpis was found in all areas.

Answer. The phlebotominic fauna of the region was added to the discussion section. Lines 601-608. In Teodoro Sampaio, although an entomological survey started in 2000, Lu. longipalpis sand flies were found only in 2010. It is well known that other phlebotomines, such as Lu. migonei, may be permissive vectors of VL in areas where Lu. longipalpis has not been recorded, but human or canine cases do occur [28-30]. According to the SUCEN database, in the period from 2000 to 2010, Lu. cruzi a VL vector found in Central-West of Brazil [31], and Lu. migonei were not identified in Teodoro Sampaio urban area. Lu. migonei was found in the wild forest of the Devil's Hill in research carried out for an LT investigation in 1994, 1995, and 1996.

Lines 374-375 Spatiotemporal distribution of *Lu. longipalpis* sandflies in the urban area of Teodoro Sampaio, São Paulo, Brazil. The amount of specimens collected, the time between positive collections and without the information that they are male or female. I believe that a longer collection period would be important. What is 1M?

Answer: Table 1 was corrected according to your comments.
Lines 245-249: In the urban area of Teodoro Sampaio, the phlebotomine sand fly entomological survey began in 2000, monthly, from February to October during the most favourable periods for detecting the presence of the vector. The survey ended in October 2010 when Lu. longipalpis was found in all areas.

Line 457 *The Discussion must be rewritten. The relationship of visceral Leishmaniasis. leishmania, vector, host, from this study with other articles on the same theme or subject. It should be discussed.*
The discussion was rewritten and the main results compared to the scientific literature.

Lines 512-514. *Canis familiaris,* which in turn infect humans, the definitive host. The role of the environment and urban ecosystems in the tracks of the spread of vectors of yellow fever virus in Brazil was demonstrated recently [16].
Answer: the text was removed.

Lines 526-530: References: (22,23)
Lines 538-543: The paragraph was deleted as suggested.
Lines 553-558: The paragraph was deleted as suggested.
Lines 589-590: The paragraph was deleted as suggested.
Lines 662-663: The text was removed to conclusion as suggested.

691 - After reviewing the text, references must be updated
Answer: the references were updated.

Click here to access/download
Revised Article with Changes Highlighted
Revised Article with changes highlighted.docx
