# Supplementary material for: Cases and distribution of visceral leishmaniasis in western São Paulo: A neglected disease in this region of Brazil
Source: PLoS Negl Trop Dis. 2021 Jun 15;15(6):e0009411. doi: 10.1371/journal.pntd.0009411 (PMC8232419; doi:10.1371/journal.pntd.0009411)
Supplement: Attachment — Submitted filename: 21-02-2021 RESPONSE TO THE REVIEWERS.docx [file pntd.0009411.s003.docx]

**Answers to the PDF comments: Reviewer 2 Pages 8-12**

**Reviewer's Responses to Questions**

The revised manuscript was sent to a native speaker: Language edit Revised Article: Cases and distribution of visceral leishmaniasis in western São Paulo: a neglected disease in this region of Brazil

**<b>Key Review Criteria Required for Acceptance?</b></br></br>
As you describe the new analyses required for acceptance, please consider the following:</br></br>

<b>Methods</b></br></br>
-Are the objectives of the study clearly articulated with a clear testable hypothesis stated?</br>
-Is the study design appropriate to address the stated objectives?</br>
-Is the population clearly described and appropriate for the hypothesis being tested?</br>
-Is the sample size sufficient to ensure adequate power to address the hypothesis being tested?</br>
-Were correct statistical analysis used to support conclusions?</br>
-Are there concerns about ethical or regulatory requirements being met?</br></br>**

**Reviewer #1** – Dear Dr. Thanks for your time and valuable contribution to our manuscript. We tried to fill in all the issues raised in your comments, however, if new amendments are necessary, we will try to make them.

The manuscript "The burden of visceral leishmaniasis in western São Paulo: a neglected disease in this region of Brazil " aims to determine the LV burden in 29 municipalities of the Regional Health Assistance Network (RNHA11), west of São Paulo, in a multiscale approach centered on the city of Teodoro Sampaio.  However, the authors do not propose any hypothesis in the introduction section. They also do not raise questions about the entry and spread of the disease in the region. In the description it is necessary to be more clear which data were obtained from databases and which were performed by the authors.

*Answer. The manuscript was re-structured according to your suggestion and comments.*

***In the Introduction section****, a description of the entry and dispersion of VL was ruled out. Page 4 - Lines 69-82*

*A hypothesis was performed on the role of socioeconomic and environmental risk factors in the spreading of Lu. longipalpis, infected dogs, and human visceral leishmaniasis in the western region of São Paulo state. Page 4 – Lines 83-92.*

*In addition, following your observation, we divided the methodology and results on a regional scale with data obtained in public databases, and at the local scale, focusing on the city of Teodoro Sampaio, with data performed by the authors. Page 4 – Lines 92-96.*

***In the Methods section***

***First****, an approach on a regional scale, using public databases was focused, as follow:* *Study design and setting:*

*Page 5 – Lines 101-116: Regional characteristics; Page 6 – Lines 117-141: Local characteristics*

*Data on the presence of Lu. longipalpis in the municipalities of RNHA11 Page 8 – Lines 173-183*

## *Canine and human visceral leishmaniasis and Center of Zoonosis Control in the municipalities of RNHA11mesoregion – Page 8 – Lines 184-209*

## *Mapping the cluster of identification of canine visceral leishmaniasis in RNHA11 mesoregion – Page 9 – Lines 210-228*

## *Mapping the Human Development Index in the RNHA11 mesoregion: Page 10 – Lines 229-242*

## *Second, we presented the results at a local scale focusing in the municipality of Teodoro Sampaio, a representative municipality of Pontal of Paranapanema, and of the RNHA-11, which data were performed by the authors, as follow:*

## *Entomological survey in Teodoro Sampaio: Page 12 – Lines 243-262*

## *Canine visceral leishmaniasis survey in Teodoro Sampaio: Page 12 – Lines 263-287*

## *Human visceral leishmaniasis in Teodoro Sampaio: Page 12-13 – Lines 288-294*

## *Human visceral leishmaniasis serological survey and laboratory diagnosis in Teodoro Sampaio: Page 14 – Lines 295-318*

**Reviewer #2:** *Dear Dr. We are grateful for your correction, comments, and suggestions in the PDF file of the manuscript. We are aware that the time you spent on this work is precious. We hope to fit all the issues raised in your comments. The detailed answers and corrections of your comments are in the PDF file brackets.*

The objectives of the study are clearly articulated. The study design is a little bit confuse for the reader. The population studied (human, vectors and dogs) are described in a disorderly way, in my opinion. It becomes a bit difficult to read the paper. The manuscript contributes with original and useful information related to several aspects of leishmaniasis in a poor area**.**

*Answer: According to your suggestion, to be more understandable, the Study Design, Methods, and Results sections were amended as follow:*

***In the Introduction section****, a description of the entry and dispersion of VL was ruled out. Page 4 - Lines 69-82*

*A hypothesis was performed on the role of socioeconomic and environmental risk factors in the spreading of Lu. longipalpis, infected dogs, and human visceral leishmaniasis in the western region of São Paulo state. Page 4 – Lines 83-92.*

*In addition, following your observation, we divided the methodology and results on a regional scale with data obtained in public databases, and at the local scale, focusing on the city of Teodoro Sampaio, with data performed by the authors. Page 4 – Lines 92-96.*

***In the Methods section***

***First****, an approach on a regional scale, using public databases was focused, as follow: Study design and setting:*

*Page 5 – Lines 101-116: Regional characteristics; Page 6 – Lines 117-141: Local characteristics*

*Data on the presence of Lu. longipalpis in the municipalities of RNHA11 Page 8 – Lines 173-183*

## *Canine and human visceral leishmaniasis and Center of Zoonosis Control in the municipalities of RNHA11mesoregion – Page 8 – Lines 184-209*

## *Mapping the cluster of identification of canine visceral leishmaniasis in RNHA11 mesoregion – Page 9 – Lines 210-228*

## *Mapping the Human Development Index in the RNHA11 mesoregion: Page 10 – Lines 229-242*

## *Second, we presented the results at a local scale focusing in the municipality of Teodoro Sampaio, a representative municipality of Pontal of Paranapanema, and of the RNHA-11, which data were performed by the authors, as follow:*

## *Entomological survey in Teodoro Sampaio: Page 12 – Lines 243-262*

## *Canine visceral leishmaniasis survey in Teodoro Sampaio: Page 12 – Lines 263-287*

## *Human visceral leishmaniasis in Teodoro Sampaio: Page 12-13 – Lines 288-294*

## *Human visceral leishmaniasis serological survey and laboratory diagnosis in Teodoro Sampaio: Page 14 – Lines 295-318*

**Reviewer #3:** Dear Reviewer: Thanks for considering our manuscript for revision. Your expertise will certainly contribute to the improvement of the article. We tried to fill in all the issues raised in your comments, however, if new amendments are necessary we will try to make them.

Are the objectives of the study clearly articulated with a clear testable hypothesis stated? No, the authors used data from other public bodies as part of the methodology. For example: Data from the Supervision in Control of Epidemic- (SUCEN), Center of Regional Laboratory of the Adolfo Lutz Institute of Presidente Prudente V (CRL-CBSALI-PPV), National System of Diseases Notification (SINAN), Epidemiological Surveillance Center (CVE-SP). Considering each institution, the methodology used by them to obtain their results is different. If you need to redo this search. It will be necessary to obtain all this data and more that they have and if it is possible to do so.

*Answer: To get the manuscript more understandable, we divided the methodology and the results on a regional scale, using databases, obtained from public agencies, focusing on vectors, canine visceral leishmaniasis, human leishmaniasis, and Human Developing Index. Using Teodoro Sampaio, considered the Pontal of Paranapanema and one of the poorest regions of São Paulo state, on a local scale, data obtained by the authors on the entomological survey, canine and human visceral leishmaniasis was discussed. Furthermore, on a regional scale, we added a statistical analysis using Moran's I and LISA methods to identify spatial autocorrelation and to generate cluster maps as priority areas for implementing surveillance and control actions.*

**At the regional scale**, public databases were provided by:

Data of *Lu. longipalpis* was provided by superintendencia-de-controle-de-endemias (SUCEN). SUCEN is a public agency of São Paulo state with the mission of coordinating, monitor and evaluate vector surveillance and control actions, intermediate hosts, and venomous arthropods, as well as producing and disseminate scientific- technical knowledge and share technologies for health surveillance. The data obtained from SUCEN was coordinated by the director of SUCEN in Presidente Prudente: Ivete da Rocha Anjolete.

Data of canine visceral leishmaniasis. Adolfo Lutz Institute (ALI) is the public former laboratory in São Paulo state responsible for the laboratory confirmation of canine visceral leishmaniasis in the whole state. At RNHA11, the ALI coordinates the actions on laboratory diagnosis in all municipalities. Data on CVL and the presence of Zoonosis Center Control was coordinated by Lourdes D’Andrea Zampieri.

Data on human visceral leishmaniasis. HVL data was obtained from the Epidemiological Surveillance Center Alexandre Vranjack (CVE). In São Paulo state, CVE is the public agency responsible by technical guidance on epidemiological investigation and control of diseases of interest to public health; water and foodborne diseases, respiratory diseases, vector-borne diseases and zoonoses, leprosy, tuberculosis, health ophthalmology center, hepatitis, chronic diseases. CVE coordinates the cases of human visceral leishmaniasis that occurred in the state and publishes a monthly bulletin that is publicly available.

Environment data: Data on rivers, damns, artificial lakes and highways were accessed from Instituto Brasileiro de Geografia e Estatística.

Socioeconomic risk factors: Data on Human Developing Index was obtained from Fundação Sistema Estadual de Análises de Dados (SEADE).

**On a local scale,** in Teodoro Sampaio, data was generated by the authors. The entomological survey was conducted by Ivete da Rocha Anjolete, canine visceral leihsmaniasis was conducted by Lourdes D’Andrea Zampieri, human VL survey was conducted by Regiane Soares, Karina Briguenti Souza, Cristiane Oliveira Andrade, and Luiz Euribel Prestes Carneiro. The ELISA tests were performed by Lourdes D’Andrea Zampieri and laboratory tests performed in the HVL survey individuals were performed by Marcia Kaihara Meidas.

.

**-Does the analysis presented match the analysis plan?</br>
-Are the results clearly and completely presented?</br>
-Are the figures (Tables, Images) of sufficient quality for clarity?</br></br>**

**Reviewer #1:** In my opinion, the sections "Results" and "Materials and Methods" need to be improved. The sequence of data and a title are not very didactic for the readers. They must be put in the same order.

*Answer: We re-organized the Methods and Results sections putting them in the same sequence. First at a regional scale with data on the 45 municipalities of the RNHA-11 mesoregion and Second, on local scale focusing on the urban area of Teodoro Sampaio, a representative municipality of the western region of São Paulo state.*

The figures from 1 to 5 are correct. However, the Figure 6 does not make much sense. It should be deleted or give more meaning to the readers.
In the reference section, many scientific words are not italicized.

*Answer: Figure 6 was re-defined and helps the readers to understand the socioeconomic and the environmental changes that is occurring in the western region. Taken together, certainly, they contribute to the spreading of vectors, infected dogs, and humans VL in RNHA11 mesoregion of São Paulo state.*

*The reference section was updated and corrected according to your observation.*

**Reviewer #2:**

In my opinion, some part of the text that appear on the results must be moved to Material and Methods.

*Answer: The subsection Potential dispersion routes of infection: RNHA11 mesoregion and Pontal of Paranapanema hub was resumed and moved to the Methods section: Study design and settings - Page 7 – Lines 141-152*

**Reviewer #3:**

Does the analysis presented match the analysis plan?
Yes

-Are the results clearly and completely presented?

No, there was no entomological research, but a collection of sandflies, the results being incomplete.

*Answer: Your comments were addressed to one of the researchers: Ivete da Rocha Anjolete, the director of the regional department of SUCEN in Presidente Prudente, who conducts the entomological surveys in the 45 municipalities of RNHA11. She revised the text and corrected the information. Page 12 – Lines 244-247.*

*Table 1 - showing the entomologic survey in Teodoro Sampaio conducted by the authors was corrected and completed.*

*The importance of knowing the phlebotomine fauna in the region was pointed out and data on Lu. migonei and Lu. cruzi in the region were added in the discussion section: Page 29 – Lines 618-625.*

-Are the figures (Tables, Images) of sufficient quality for clarity?

The exception of table 1. The other tables and images are of sufficient quality for clarity

*Answer: Table 1 was redefined as suggested, and with the objective of improving the resolution, figure B was deleted.*

**<b>Conclusions</b></br></br>
-Are the conclusions supported by the data presented?</br>
-Are the limitations of analysis clearly described?</br>
-Do the authors discuss how these data can be helpful to advance our understanding of the topic under study?</br>
-Is public health relevance addressed?</br></br>

Reviewer #1:** The manuscript is of interest to public health. However, the authors should improve the discussion section. The authors should be more concise and didactic.
*Answer: The discussion was re-written and shortened according to your suggestion. Only issues addressing VL dispersion in RNHA11 and Teodoro Sampaio were highlighted. Furthermore, the more important results on vectors, canine visceral leishmaniasis, socioeconomic and environmental data were compared with the scientific literature.*

**Reviewer #2:** Part of the discussion and conclusions must be supported by more references.

*Answer: The discussion and bibliography were updated and corrected according to your observation.*

**Reviewer #3:** Are the conclusions supported by the data presented?

Yes

Are the limitations of analysis clearly described?

Yes

Do the authors discuss how these data can be helpful to advance our understanding of the topic under study?

No, they report that this study may contribute new knowledge to the study of this neglected disease. They cite environmental, social and economic conditions as factors that can hinder actions against CVL and disease vectors, thus sustaining this disease in an endemic area.

*Answer: We tried to highlight the contributions of our data to new knowledge to study this neglected disease in the western region at the regional and local scale. Page 4 – Lines 83-96.*

*Furthermore, Moran’s I and LISA cluster maps were created to identify priority areas for implementing surveillance and control actions.*

*At the regional scale, combining data of VL with environmental and socioeconomic risk factors allows constructing the potential dispersion routes and the municipalities with higher socioeconomic and environmental vulnerability. Knowing dispersion routes is essential to develop preventive measures to control the disease and restrain its future dispersion. Knowing the environmentally/socioeconomic related vulnerable municipalities may transform governmental public health and community education actions more effective in controlling the endemic transmission of the disease in the dominium of RNHA11. In Teodoro Sampaio, most of the infected dogs were found in the periphery and low levels were euthanized. They may be disseminating the disease to rural and neighboring áreas free of VL. Surveys conducted in vectors, reservoirs, and humans can be used by local and regional authorities, state and countrywide for understanding the VL transmission, its fragilities and taking coordinated measures to control the disease at the local and regional scale.*

-Is public health relevance addressed?

Yes

--------------------

Answers to the PDF comments: Reviewer 2

**Abstract**

It would not be **cases** of visceral leishmaniasis:

*Answer: The title was changed according to your suggestion:* *Cases and distribution of visceral leishmaniasis in western São Paulo: a neglected disease in this region of Brazil*

*How many municipalities are there? include the number of municipalities.*

Answer: 45 municipalities

*Answer: The number of municipalities were included as follow: Lines 24-34: To study this neglected disease on a regional scale, we describe the spread of VL in 45 municipalities of the Regional Network for Health Assistance11(RNHA11).*

Line 34. For the local study in Teodoro Sampaio, an entomological survey from 2009 to 2015. *Inform, the location of the collection (intradomicile, peridomicile, forest, etc.), the start and end time of the collections, were the catches fortnightly or monthly?*

35. 2015, a canine visceral leishmaniasis (CVL) survey from 2010 to 2018 by enzyme-linked immunosorbent assays ELISA, and a human serum survey in 2018 were carried out. *What was the methodology used to survey CVL cases?*

*Answer: Lines 32-34 In Teodoro Sampaio, monthly peridomicile sand flies collection; ELISA, IFAT and Rapid Test serological canine VL (CVL); and an ELISA human VL (HVL) serum surveys were carried out.*

Lines 37,38. From 2005 to 2018, *Lutzomyia longipalpis* was found in 38 (77.8%),

CVL in 68%, and HVL in 42.2% of the municipalities. *You are confused, please, in the entomological survey carried out from 2005 to 2018, where was found Lu longipalpis, is it included the research done in Teodoro Sampaio from 2009 to 2015, by the authors?*

*Answer:* *The methodology was corrected as follow: Lines 24-34*

*To study this neglected disease on a regional scale, we describe the spread of VL in 45 municipalities of the Regional Network for Health Assistance11(RNHA11). Environmental, human VL (HVL), and canine VL (CVL) cases, Human Development Index, and Lutzomyia longipalpis databases were obtained from public agencies.* *Global Moran's I index and local indicators of spatial association (LISA) statistics were used to identify spatial autocorrelation and to generate maps for the identification of VL clusters. On a local scale, we determined the spread of VL in the city of Teodoro Sampaio, part of the Pontal of Paranapanema. In Teodoro Sampaio, monthly peri-domicile sand fly collection; ELISA, IFAT and Rapid Test serological CVL; and ELISA human HVL serum surveys were carried out.*

*Answer: A detailed information on the entomological survey was added to Methods and Results sections: Lines 245-248: In the urban area of Teodoro Sampaio, the phlebotomine sand fly entomological survey began in 2000, monthly, from February to October during the most favourable periods for detecting the presence of the vector. The survey ended in October 2010 when Lu. longipalpis was found in all areas.*

Line185. Four traps were installed for three nights in each sector. *Were these collections fortnightly or monthly?*

*Answer: Lines 245-248: In the urban area of Teodoro Sampaio, the phlebotomine sand fly entomological survey began in 2000,* ***monthly****, from February to October during the most favourable periods for detecting the presence of the vector. The survey ended in October 2010 when Lu. longipalpis was found in all areas.*

*Line 258:* *Monthly, four traps were installed for three nights in each sector.*

*Monthly, from February to October during the most favorable periods for detecting the presence of the vector.*

Lines 190-192. In 2009 when the survey started in the urban area of

Teodoro Sampaio, the traps were installed in four areas concomitantly. In 2010, the

entomology survey ended after the vector was found in all areas. *In the summary of the article, it appears that an entomological survey was carried out from 2009 to 2015. However, in this paragraph it is informed that the entomological research, in the urban area of Teodoro Sampaio, took place from 2009 to 2010. In this period, in addition to Lu. longipalpis, what species were found? The literature points out, the meeting of Lu cruzi as an LV vector in Corumbá, Mato Grosso. The encounter of Lu migonei naturally infected with Leishmania infantum chagasis in Pernambuco. The importance of knowing the phlebotomine fauna is supported by the findings mentioned above. From 2011 to 2015, was there a collection? And where was it done?*

*Entomological research has to be done at least in two years, in the case in question what happened was a collection of sandflies.*

*Answer: We separated data from regional scale joining data of the 45 municipalities of RNHA-11 obtained from public databases.*

## *Lines 175-185:* *Data on the presence of Lu. longipalpis in the municipalities of RNHA11*

*In RNHA11, data on phlebotomine sand flies identified in entomological collections were obtained from SUCEN in the period from 2000 to 2019.* *Entomologic surveys are proposed by the Manual of Surveillance and Control of Visceral Leishmaniasis of São Paulo (VLCPSP) [11], aiming to monitor the distribution of Lu. longipalpis in silent non-receptive, non-vulnerable municipalities, and silent non-receptive vulnerable municipalities as defined by the absence of confirmed autochthonous cases of HVL and CVL, without the presence of the chosen vector or not by estimated values of the distance to characterize the vulnerability. In these municipalities, an entomologic survey is recommended for the detection of the presence of Lu. longipalpis and to provide information on its distribution, allowing identification of the risk areas where control measures should be intensified [11].*

## *Lines 244-248 Entomological survey in Teodoro Sampaio*

## *Entomological survey in Teodoro Sampaio*

*In the urban area of Teodoro Sampaio, the phlebotomine sand fly entomological survey began in 2000, monthly, from February to October during the most favourable periods for detecting the presence of the vector. The survey ended in October 2010 when Lu. longipalpis was found in all areas.*

*We are in line with your opinion on the importance of knowing the phlebotomine fauna in the region and new data was added to the discussion section. Lines 600-607:* *In Teodoro Sampaio, although an entomological survey started in 2000, Lu. longipalpis sand flies were found only in 2010.* *It is well known that other phlebotomines, such as Lu. migonei, may be permissive vectors of VL in areas where Lu. longipalpis has not been recorded, but human or canine cases do occur [28-30]. According to the SUCEN database, in the period from 2000 to 2010, Lu. cruzi a VL vector found in Central-West of Brazil [31], and Lu. migonei were not identified in Teodoro Sampaio urban area. Lu. migonei was found in the wild forest of the Devil’s Hill in research carried out for an LT investigation in 1994, 1995, and 1996.*

Line 208 - Publication reference

*Answer: suspicious for CVL, as previously published [9].*

Line 278 was obtained in SEADE, *This acronym is appearing for the first time. Write in full and put the acronym in brackets*

*Answer: Fundação Sistema Estadual de Análise de Dados*

Line 302 Maps were constructed using a geographic information system. *Which geographic information system?*

*Answer: Line 355 Maps were constructed using ArcGIS 10.7 and ArcGis Pro.*

Lines 305-325 - *This text describes the geography and highways of the studied site, which are already known public information. Citing endemic VL regions. And they were put as results. What are the results? What is intended proves with this description. I believe that, it would be better if it were placed in the “Material and Method. I did not understand. Please delete or rewrite.*

## *Answer: The text was shortened and sent to Methods section; Study design and settings. Lines 143-162:* Potential dispersion routes of VL infection in RNHA11 mesoregion

*RNHA11 is connected by two important radial highways: (1) the Marechal Candido Rondon (SP 300) linking two VL endemic regions: Três Lagoas, on the border of MS and Araçatuba-Bauru in the center-northeastern region of São Paulo; (2) the Euclides Figueiredo highway (SP 563) linking the VL endemic regions of Pereira Barreto, Ilha Solteira, and Andradina to Dracena, one of the most important cities of RNHA11, also with the highest prevalences in VL. Secondary highways also link the RNHA11 from north to south in direction to Paraná state, a region free of VL so far. Teodoro Sampaio is connected by a network of radial and secondary highways. One of the most important is the secondary vicinal highway (VSP-031; VSP-035) connecting the endemic regions of Presidente Epitácio and Panorama, bordering the Paraná River to Teodoro Sampaio (Fig1A)*

Line 365: **Entomological survey of Lu. longipalpis in the urban area of Teodoro Sampaio.** Entomological research must be carried out in at least two years, with the aim of comparing the results found. Collections were made in the urban area of Teodoro Sampaio. And only Lu. longipalpis was collected.? What is the phlebotominic fauna of the region? It would be interesting to record this data.

*Answer: The text was corrected as follow: Lines 245-249: In the urban area of Teodoro Sampaio, the phlebotomine sand fly entomological survey began in 2000, monthly, from February to October during the most favourable periods for detecting the presence of the vector. The survey ended in October 2010 when Lu. longipalpis was found in all areas.*

*Answer. The phlebotominic fauna of the region was added to the discussion section. Lines 601-608. In Teodoro Sampaio, although an entomological survey started in 2000, Lu. longipalpis sand flies were found only in 2010. It is well known that other phlebotomines, such as Lu. migonei, may be permissive vectors of VL in areas where Lu. longipalpis has not been recorded, but human or canine cases do occur [28-30]. According to the SUCEN database, in the period from 2000 to 2010, Lu. cruzi a VL vector found in Central-West of Brazil [31], and Lu. migonei were not identified in Teodoro Sampaio urban area. Lu. migonei was found in the wild forest of the Devil’s Hill in research carried out for an LT investigation in 1994, 1995, and 1996.*

Lines 374-375 Spatiotemporal distribution of *Lu. longipalpis* sandflies in the urban area of Teodoro Sampaio, São Paulo, Brazil. The amount of specimens collected, the time between positive collections and without the information that they are male or female. I believe that a longer collection period would be important. What is 1M?

*Answer: Table 1 was corrected according to your comments.*

*Lines 245-249: In the urban area of Teodoro Sampaio, the phlebotomine sand fly entomological survey began in 2000, monthly, from February to October during the most favourable periods for detecting the presence of the vector. The survey ended in October 2010 when Lu. longipalpis was found in all areas.*

Line 457 *The Discussion must be rewritten. The relationship of visceral Leishmaniasis. leishmania, vector, host, from this study with other articles on the same theme or subject. It should be discussed.*

*The discussion was rewritten and the main results compared to the scientific literature.*

*Lines* 512-514. *Canis familiaris,* which in turn infect humans, the definitive host. The role of the environment and urban ecosystems in the tracks of the spread of vectors of yellow fever virus in Brazil was demonstrated recently [16].

Answer: the text was removed.

Lines 526-530: References: (22,23)

Lines 538-543: The paragraph was deleted as suggested.

Lines 553-558: The paragraph was deleted as suggested.

Lines 589-590: The paragraph was deleted as suggested.

Lines 662-663: The text was removed to conclusion as suggested.

691 - After reviewing the text, references must be updated

Answer: the references were updated.
