# Supplementary material for: Cases and distribution of visceral leishmaniasis in western São Paulo: A neglected disease in this region of Brazil
Source: PLoS Negl Trop Dis. 2021 Jun 15;15(6):e0009411. doi: 10.1371/journal.pntd.0009411 (PMC8232419; doi:10.1371/journal.pntd.0009411)
Supplement: Attachment — Submitted filename: RESPONSE TO REVIEWER.docx [file pntd.0009411.s005.docx]

RESPONSE TO REVIEWER 3.

Dear Dr. João Ricardo Carreira Alves,

Thank you again for your comments on the revised manuscript. Certainly, your expertise in the epidemiological investigation of Leishmaniasis cases, particularly in Phlebotominae vectors, brought a great contribution to the manuscript. We accept all your observations and the amendments carried out as follows.

1 - Lines 105, 112, 116, 123 and 375. The reference related to IBGE 2010 was added to the text as well as in the bibliography (reference [8]) and the bibliography adjusted.

Lines 720-722 - 8. Instituto Brasileiro de Geografia e Estatística (IBGE). Portal do IBGE. Cidades e Estados/Portal de Mapas. Available from: https://www.cidades.ibge.gov.br/brasil/sp/panorama

2 -Lines 541-550: If this text is part of the discussion, it should be inserted into it. Otherwise, delete

Following your suggestion, the legend of Figure 7 was deleted. All the socioeconomic and environmental aspects involved in the spreading of VL in the western region were highlighted in the discussion section. So, the legend makes no sense.

3 -Lines 539-540: The authors cite Fig. 7 at the conclusion, but it is necessary that this figure is at work. For it to be analyzed at the conclusion. Where is Fig. 7? The figure should not be inserted at the conclusion, just write the reference.

The text was corrected and the reference was cited in the conclusion as follows:

Lines 672-673: Socioeconomic and environmental factors may be fueling the epidemic and sustaining the endemic transmission of the disease (Fig 7).

Line 756 - The reference was corrected
